# Comparative genomic analysis identifies structural features of CRISPR-Cas systems in *Riemerella anatipestifer*

**DOI:** 10.1186/s12864-016-3040-4

**Published:** 2016-08-30

**Authors:** De-Kang Zhu, Xue-Qin Yang, Yang He, Wang-Shu Zhou, Xiao-Heng Song, Jiang-Bo Wang, Yu Zhang, Ma-Feng Liu, Ming-Shu Wang, Ren-Yong Jia, Shun Chen, Kun-Feng Sun, Qiao Yang, Ying Wu, Xiao-Yue Chen, An-Chun Cheng

**Affiliations:** 1 Research Center of Avian Diseases, College of Veterinary Medicine of Sichuan Agricultural University, Chengdu, Sichuan China; 2 Key Laboratory of Animal Disease and Human Health of Sichuan Province, Chengdu, Sichuan China; 3 Institute of Preventive Veterinary Medicine of Sichuan Agricultural University, Chengdu, Sichuan China

**Keywords:** *Riemerella anatipestifer*, CRISPR-Cas system, *cas* gene, repeat sequence, spacer sequence, phylogenetic analysis

## Abstract

**Background:**

*Riemerella anatipestifer* infection is a contagious disease that has resulted in major economic losses in the duck industry worldwide. This study attempted to characterize CRISPR-Cas systems in the disease-causing agent, *Riemerella anatipestifer* (*R. anatipestifer*). The CRISPR-Cas system provides adaptive immunity against foreign genetic elements in prokaryotes and CRISPR-*cas* loci extensively exist in the genomes of archaea and bacteria. However, the structure characteristics of *R. anatipestifer* CRISPR-Cas systems remains to be elucidated due to the limited availability of genomic data.

**Results:**

To identify the structure and components associated with CRISPR-Cas systems in *R. anatipestifer*, we performed comparative genomic analysis of CRISPR-Cas systems in 25 *R. anatipestifer* strains using high-throughput sequencing. The results showed that most of the *R. anatipestifer* strains (20/25) that were analyzed have two CRISPR loci (CRISPR1 and CRISPR2). CRISPR1 was shown to be flanked on one side by *cas* genes, while CRISPR2 was designated as an orphan. The other analyzed strains harbored only one locus, either CRISPR1 or CRISPR2. The length and content of consensus direct repeat sequences, as well as the length of spacer sequences associated with the two loci, differed from each other. Only three *cas* genes (*cas1*, *cas2* and *cas9*) were located upstream of CRISPR1. CRISPR1 was also shown to be flanked by a 107 bp-long putative leader sequence and a 16 nt-long anti-repeat sequence. Combined with analysis of spacer organization similarity and phylogenetic tree of the *R. anatipestifer* strains, CRISPR arrays can be divided into different subgroups. The diversity of spacer organization was observed in the same subgroup. In general, spacer organization in CRISPR1 was more divergent than that in CRISPR2. Additionally, only 8 % of spacers (13/153) were homologous with phage or plasmid sequences. The *cas* operon flanking CRISPR1 was observed to be relatively conserved based on multiple sequence alignments of Cas amino acid sequences. The phylogenetic analysis associated with Cas9 showed Cas9 sequence from *R. anatipestifer* was closely related to that of *Bacteroides fragilis* and formed part of the subtype II-C subcluster.

**Conclusions:**

Our data revealed for the first time the structural features of *R. anatipestifer* CRISPR-Cas systems. The illumination of structural features of CRISPR-Cas system may assist in studying the specific mechanism associated with CRISPR-mediated adaptive immunity and other biological functions in *R. anatipestifer*.

**Electronic supplementary material:**

The online version of this article (doi:10.1186/s12864-016-3040-4) contains supplementary material, which is available to authorized users.

## Background

*Riemerella anatipestifer* (*R. anatipestifer*) is a Gram-negative bacterium belonging to the *Flavobacteriaceae* family. This bacterium is the causative agent of *R. anatipestifer* infection, which result in septicemic disease in ducks, geese, turkeys, and other birds. The associated disease represents a major problem for the duck industry worldwide, and is the cause of significant economic losses [1].

Clustered regularly interspaced short palindromic repeats (CRISPR) and the CRISPR-associated (Cas) proteins constitute the CRISPR-Cas system. This system provides a novel adaptive immunologic mechanism against exogenous nucleic acid invasion in archaea and bacteria [2–4]. The CRISPR-*cas* locus is found to be widespread in prokaryotes, being present in ~50 % of sequenced bacterial genomes and ~87 % of sequenced archaea genomes [5, 6]. The CRISPR-*cas* locus consists of a CRISPR array and a set of *cas* genes [2–4]. Moreover, a leader sequence is involved occasionally [7–11]. The CRISPR array is composed of direct repeat sequences (repeats) and spacer sequences (spacers) that are derived from phages, plasmids or other mobile genetic elements [2–4]. The corresponding sequences in foreign genetic elements are called protospacers [2–4, 12, 13]. The leader sequence is AT-rich, and is located upstream of CRISPR array [7–9]. This sequence is considered the promoter for the CRISPR locus in some strains [9–11]. The immunologic mechanism employed by the CRISPR-Cas system commonly consists of three steps. In the acquisition step, a fragment of exogenous genetic elements integrates into the CRISPR array, generating a new repeat-spacer unit [14–17]. During the expression step, the CRISPR array is transcribed and processed into CRISPR RNA (crRNA), which is bound to a single Cas protein or to a multi-subunit complex comprised of multiple Cas proteins [18–20]. Then the crRNA guides the Cas protein(s) to target and cleave the protospacer of cognate exogenous nucleic acids in the interference step [21–24]. However, the specific molecular mechanisms vary depending on the system used. Additionally, *trans*-encoded CRISPR RNAs (tracrRNA) have been identified and are considered an additional factor of type II systems [20, 25]. The tracrRNAs are encoded by sequences found close to the *cas* operon and CRISPR array and are distinguished by the presence of an anti-repeat sequence complement with cognate repeats [20, 25]. During interference, in addition to complementarity between protospacer and the spacer portion of crRNA, a defined sequence (known as protospacer adjacent motif or PAM) musts flank on one side of the protospacer to avoid self-targeting. Moreover, PAM is involved in spacer acquisition [26–29].

Nowadays, the CRISPR-Cas systems can be divided into two classes, five types and 16 subtypes [30]. Class 1 systems, with multi-subunit effector complexes made up of multiple Cas proteins, include type I, type III and putative type IV. Class 2 systems, with a single Cas protein effector, encompass type II and putative type V. Based on distinct Cas protein composition and architecture of *cas* operon, the five main types can be further classified into sixteen subtypes. Each of the five types has its own signature *cas* gene, which is *cas3* (or its variant *cas3’*), *cas9*, *cas10*, *csf1* and *cpf1* respectively [30]. The latest research has discovered that there are three novel Class 2 systems, namely, two subtypes of putative type V (characterized by C2c1 and C2c3 effector proteins respectively) and putative type VI (C2c2 is its effector protein) [31]. Each grouping harnesses specific molecular mechanism although these systems share main functional modules and play a role in immunity [30–32]. To date, because of limitations pertaining to genomic sequence data for *R. anatipestifer*, structural analyses of *R. anatipestifer* CRISPR-Cas systems have not been performed. In this study, we performed sequence analysis of the CRISPR-Cas systems from 25 different strains, including 18 clinical strains that had previously been isolated by our research group.

## Methods

### *R. anatipestifer* strains

Eighteen clinical strains were isolated from the livers of infected ducks from seven different provinces in China. The information of bacterial strains utilized is listed in Additional file 1: Table S1.

Six complete genomes of *R. anatipestifer* strains (DSM 15868, CP002346.1; RA-GD, CP002562.1; CH3, CP006649.1; strain 153, CP007504.1; Yb2, CP007204.1; and strain 17, CP007503.1) were retrieved from National Center for Biotechnology Information (NCBI, http://www.ncbi.nlm.nih.gov/genome) [33]. The accession numbers of these complete genomes were listed in Additional file 1: Table S1.

### Genomic DNA extraction and sequencing

Genomic DNA was extracted with a TIANamp Bacteria DNA Kit (Tiangen Biotech Co, Ltd., Beijing, China). Then the sequences of these strains analyzed were determined using a high-throughput sequencing platform (Illumina HiSeq 2500) with an average genome coverage of 100X. Velvet version 1.2.09 [34] was used for *de novo* assembly. NCBI Prokaryotic Genome Annotation Pipeline (PGAP) (http://www.ncbi.nlm.nih.gov/genome/annotation_prok) was utilized to facilitate genome annotation. All of the genome sequences were submitted to NCBI, with the most relevant information listed in Additional file 1: Table S1.

### Phylogenic analysis of *R. anatipestifer* strains

A phylogenetic tree (Neighbor Joining Tree) of the nineteen sequenced genomes and six NCBI deposited complete genomes was constructed using a pangenome analysis tool-BPGA [35] based on pangenome of *R. anatipestifer*. This analysis utilized all default parameters.

### CRISPR-*cas* locus sequence analysis and prediction

The information pertaining to the CRISPR locus including position, length and content were acquired from CRISPRfinder (http://crispr.i2bc.paris-saclay.fr/) [36] and CRISPRI (http://crispi.genouest.org/) [37]. Loci with less than five repeat numbers were termed “questionable CRISPR loci”, and those which were not termed “confirmed CRISPR loci”, the former were not used as part of this analysis. Additionally, each of the loci obtained was manually checked as previously reported. Briefly, CRISPR loci located in coding regions should be discarded. Likewise, the loci which possesses repeats larger than 48 nt ought to be abandoned. The online tool Weblogo (http://weblogo.berkeley.edu/logo.cgi) [38] was used to analyze the conservation of the associated repeats, and secondary structure prediction of the repeats was performed using Mfold (http://unafold.rna.albany.edu/?q=mfold/RNA-Folding-Form) [39]. The spacers for each locus were manually identified and compared. CRISPRTarget (http://bioanalysis.otago.ac.nz/CRISPRTarget/crispr_analysis.html) [40] was utilized to predict the presence of possible protospacers. Protospacers were identified if the associated sequences contained four or fewer SNPs (≥87 % identity or a minimum of 26/30 matching nucleotides) between spacers and phage or plasmid sequences. We aligned the protospacers with the flanking sequences comprising 20 bp on each side to find PAM by using Weblogo. The non-coding sequences upstream of the first repeat were selected as the putative leader sequences and aligned using ClustalX software (version 1.83) [41]. Subsequently, the putative leader sequences were screened for promoters using the BDGP Neural Network Promoter Prediction tool (http://www.fruitfly.org/seq_tools/promoter.html) [42]. The 10 kb-long sequences flanking the CRISPR array were extracted and analyzed using the BLAST program (http://blast.ncbi.nlm.nih.gov/Blast.cgi) [43] to identify *cas* genes. The composition of *cas* genes and Cas proteins in *R. anatipestifer* was analyzed using SnapGene software (version 2.3.2, from GSL Biotech; available at snapgene.com). Cas protein sequences of other bacteria were retrieved from the NCBI protein database. MEGA software (version 6) [44] was utilized to conduct the phylogenetic analysis of Cas proteins, and the phylogenetic tree associated with Cas proteins was constructed by neighbor-joining method. The default parameter values were selected when computed.

## Results

### Architecture of CRISPR-*cas* loci in *R. anatipestifer* strains

**Fig. 1:**
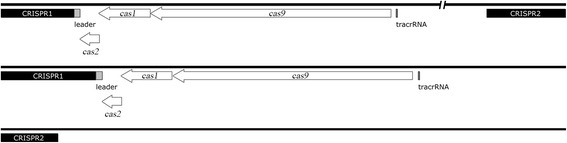
Graphic representation of CRISPR-*cas* loci in *R. anatipestifer* strains. The black blocks represent CRISPR loci. AT-rich leader sequences (light grey blocks) are located upstream of CRISPR1. There are three *cas* genes (*cas1*, *cas2* and *cas9*) which are closed to CRISPR1, shown as white boxed arrows. Upstream of *cas*9, there is a 16 nt-long anti-repeat sequence, which might be considered as a portion of the sequence that encodes for a putative tracrRNA (dark grey block). The distance between CRISPR1 and CRISPR2 is long and varies from strain to strain, thus the sequence between the two loci is omitted and is represented by a double-slash

In the 25 strains that were analyzed, 45 CRISPR loci were identified. These loci were divided into two groups, CRISPR1 and CRISPR2. CRISPR1 was flanked by *cas* genes which encode for Cas proteins. However, no *cas* genes were in the vicinity of CRISPR2. Twenty-four and 21 different arrays were observed for CRISPR1 and CRISPR2, respectively. Twenty of the analyzed strains contained both loci (CRISPR1 and CRISPR2), while the other strains contained only one locus, ie, CRISPR1 or CRISPR2 (RA-CH-1, CH3, RCAD0131 and RCAD0111 did not contain CRISPR2, and RCAD0133 harbored only CRISPR2) (Additional file 1: Table S2, Fig. 1). The smallest CRISPR1 locus observed in a single strain (RCAD0111) encompassed four spacers and five direct repeats, while the largest CRISPR1 locus occurred in RCAD0188 and contained 24 spacers and 25 repeats. On average, CRISPR1 locus was comprised of 14 spacers and 15 repeats. The smallest CRISPR2 locus contained eight spacers and nine repeats (Yb2), while the largest CRISPR2 locus, which was observed in five strains (RA-GD, DSM 15868, ATCC 11845, RCAD0122 and RCAD0124), contained 18 spacers and 19 repeats. CRISPR2 locus consisted of 15 spacers and 16 repeats averagely. The CRISPR locus information is displayed in Additional file 1: Table S2. The distance between CRISPR1 and CRISPR2 varied from strain to strain, approximately from 200 to 800 kb. There were only three *cas* genes (*cas1*, *cas2* and *cas9*) located upstream of the CRISPR1 locus (Fig. 1). The region between the *cas*2 gene and the first direct repeat was a 107 bp-long non-coding sequence and was defined as the putative leader sequence of CRISPR1 (Fig. 1). We found a 16 nt-long anti-repeat sequence, ‘TGAAAGCAATTCACAA’, which was located upstream of *cas9* gene in all CRISPR1-containg strains and was considered to be a part of the sequence that encodes for a putative tracrRNA (Fig. 1).

### The prediction of leader sequences

**Fig. 2:**
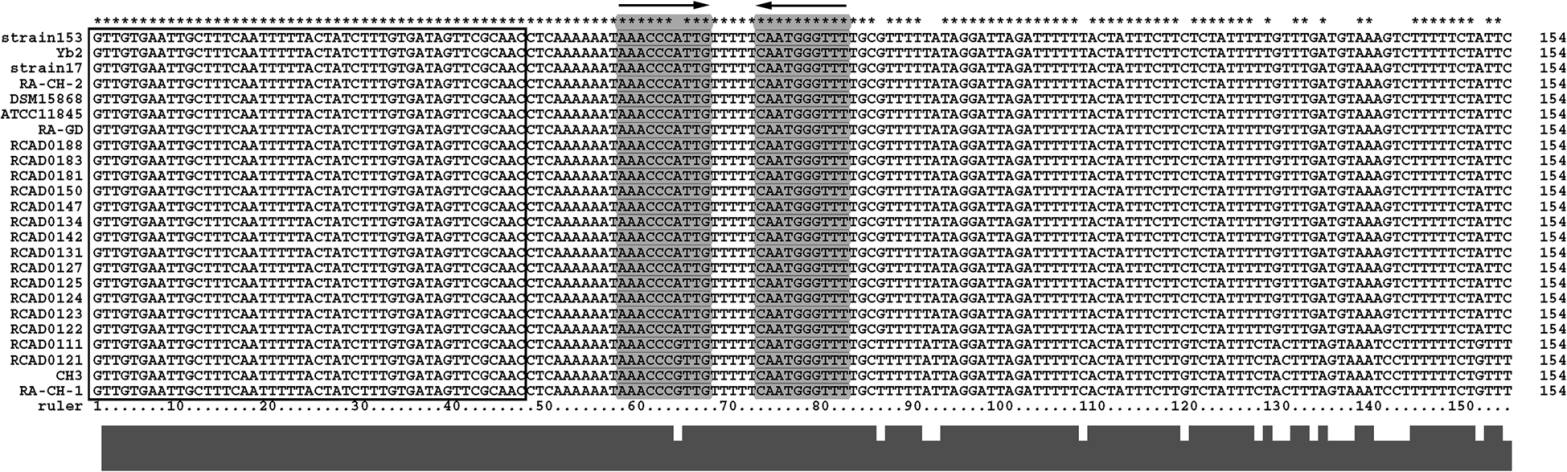
Multiple sequence alignment of putative leader sequences and the first repeats. The putative leader sequence (107 bp) and the first repeat (47 bp) of each CRISPR1 locus in analyzed strains were selected for multiple sequence alignment. The nucleotide sequences of the first repeats are framed by black box. The grey shadow and arrow indicate the palindromic sequence in leaders. The asterisk represents the conserved base in all of the sequences for alignment

According to previous reports, the leader sequence located upstream of the first CRISPR repeat is rich in A/T bases and is up to 650 bp long [7–9]. In addition, the leader sequence lacks an ORF and contains a palindromic sequence. The leader sequence has also been shown to be conserved in the same species, and is a probable recognition sequence for the insertion of new spacers [7, 8]. It may also act as a promoter for CRISPR locus [9–11]. We defined the 107 bp-long non-coding sequence between the *cas*2 gene and the first repeat as the putative leader sequence of CRISPR1. All of the predicted leader sequences were compared using ClustalX (Fig. 2). The putative leader sequences showed significant similarities between each of the analyzed strains, apart from RA-CH-1, CH3, RCAD0111, and RCAD0121. The latter strains harbored 19 SNPs in each of the putative leaders, and the SNPs that were hosted in these strains were identical. The putative leaders contained an obviously long palindromic sequence, ‘AAACCCATTGCAATGGGTTT’, separated by 5 bp (Fig. 2). The GC content of the putative leaders was 25 %, while that of *R. anatipestifer* genomes was observed to be 35 % (on average). Several promotors were predicted, and the promoter that was predicted to have the highest score (1.00, the range was 0–1) was located between 17 and 62 bp in the 107 bp putative leader sequence.

### Characteristics of direct repeat sequences

**Fig. 3:**
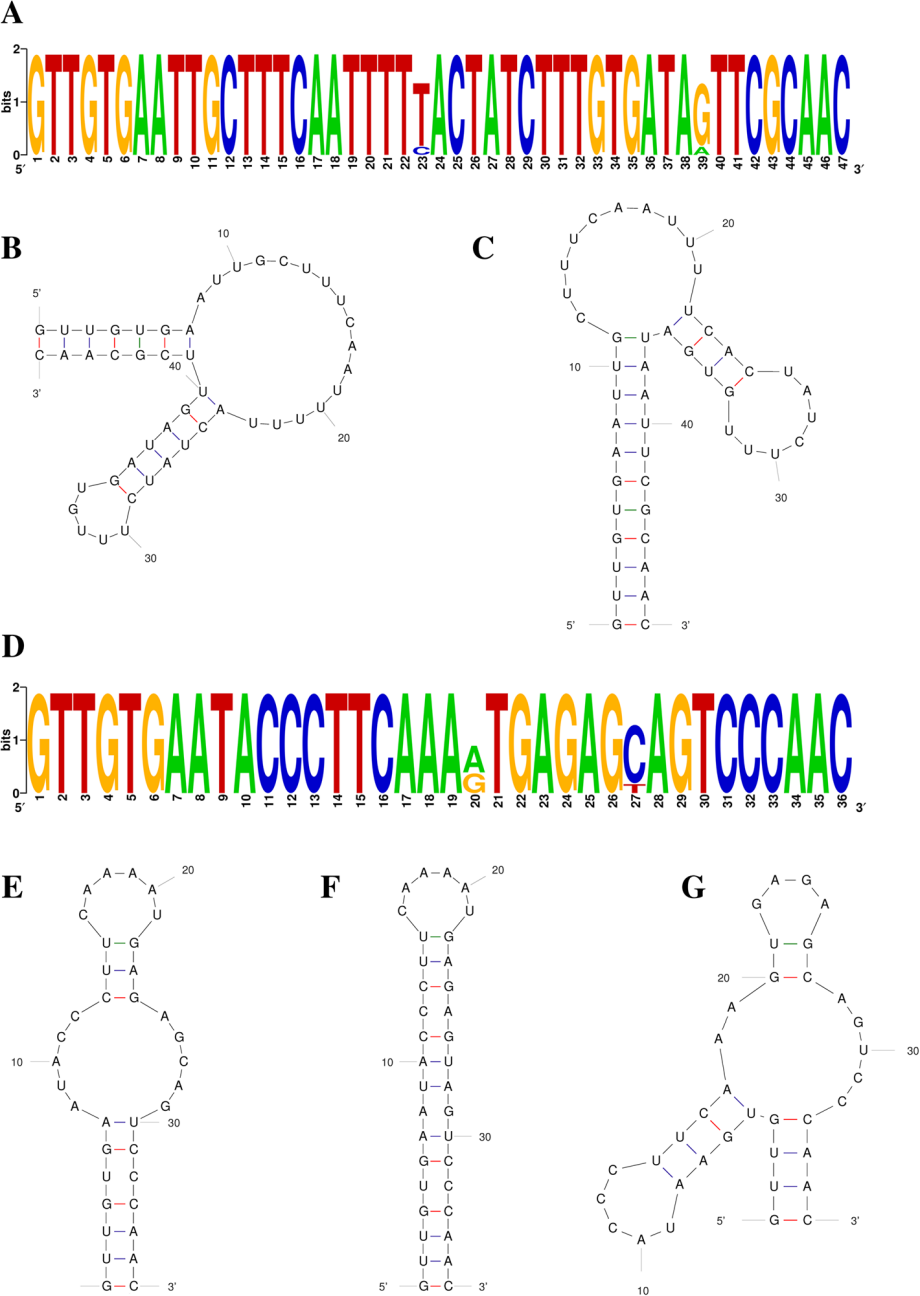
Features of repeats in *R. anatipestifer* CRISPR loci. **a** and **d** show sequence logo for consensus direct repeats associated with CRISPR1 in 20 *R. anatipestifer* strains and sequence logo for consensus direct repeats associated with CRISPR2 in five *R. anatipestifer* strains, respectively. The height of the letters indicates the relative frequency of the corresponding base at that position. **b** and **c** display predicted secondary structure of the CRISPR1 consensus direct repeats. The putative secondary structure of consensus direct repeats containing the T23C and G39A variants is shown in **c**, **e**, **f** and **g** display predicted secondary structure of the CRISPR2 consensus direct repeats. The putative secondary structures of consensus direct repeats containing the C27T variant and A20G variant are shown in (**f**) and (**g**), respectively

It has previously been reported that the length of conserved repeats varies from 21 to 48 nt [5]. These sequences have also been shown to contain palindromes. The 5’ terminal portion of a repeat is normally composed of the sequence GTTT (G) and the 3’ terminus contains the sequence GAAA (C/G) [45, 46]. Generally, repeats associated with the type II system are weakly palindromic, and are typically 36 nt in length [25, 47]. The two CRISPR loci in *R. anatipestifer* strains had different consensus direct repeat sequences and varied with respect to both length and content (Additional file 1: Table S2, Fig. 3). The length of the CRISPR1 consensus direct repeats was 47 nt, while the length of the CRISPR2 repeats was 36 nt. The consensus direct repeats associated with CRISPR1 contained a conserved 5’-GTTG terminus and a 3’-GCAAC terminus. A conserved 5’-GTTG terminus and a 3’-CCAAC terminus were present in CRISPR2. The direct repeat variants (DRVs) were observed as part of this analysis. Two cases of DRVs (ie T23C and G39A) occurred in the consensus direct repeats of CRISPR1. Both variants were observed in the CRISPR1 repeats of Yb2, RA-GD, and RCAD0147. Similarly, A20G and C27T DRVs were observed in CRISPR2. A20G appeared in the CRISPR2 repeats of strain 153, strain 17, RA-CH-2, RCAD0122, RCAD0125, RCAD0134, RCAD0188, RCAD0124, and RCAD0183, while C27T occurred in RCAD0133, RCAD0121, and RCAD0181.

The predicted secondary structures of the consensus direct repeats of CRISPR1 and CRISPR2 are shown in Fig. 3. A large number of the repeats in CRISPR1 were involved in a long stem-loop structure that was interrupted by a shorter stem-loop. Repeats containing the T23C and G39A variants produced similar secondary structures with only minimal size deviations noted for the two previously mentioned stem-loop structures. The majority of repeats in CRISPR2 had a large loop next to a small loop, while repeats containing the C27T DRV produced a small loop with a long stem only. Repeats in CRISPR2 that had been shown to contain the A20G DRV, showed more complicated stem-loop structures, which were characterized by two stem-loops occurring at a larger loop (containing a short stem).

### Spacer organization

The main differences of CRISPR loci occurred due to spacer organization diversity. Spacer sequences derived from foreign nucleic acid sequences (ie bacteriophages and plasmids), have been shown to insert into CRISPR locus in the acquisition step. Moreover, it has been confirmed that new spacers generally integrate into the leader-proximal terminus [7, 48, 49]. Thus, the organization of spacers is likely to be reflective of the degree of evolution associated with CRISPR-Cas immune defense systems.

**Fig. 4:**
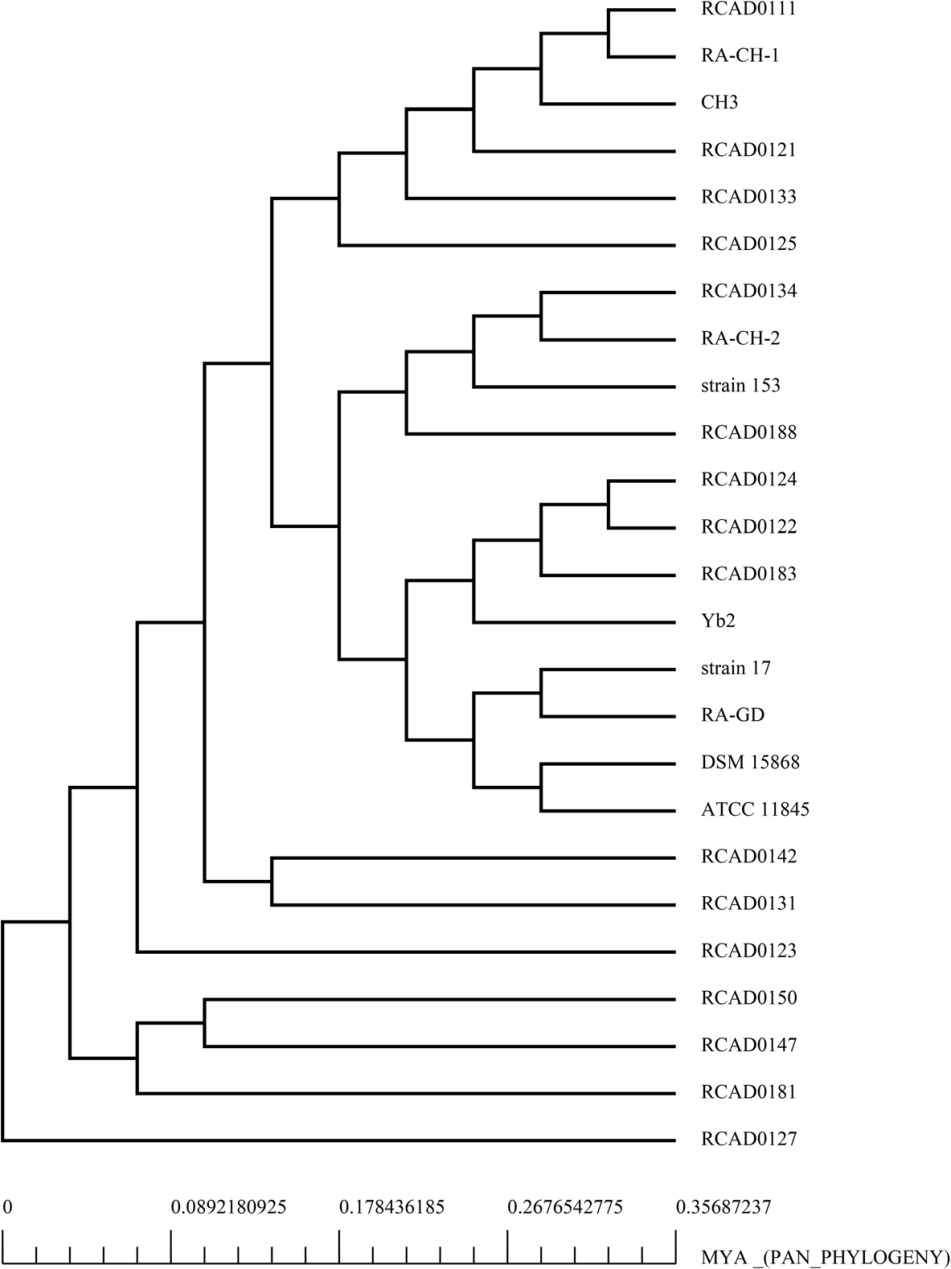
Phylogenetic analysis by BPGA based on pangenome for 25 *R. anatipestifer* strains

**Fig. 5:**
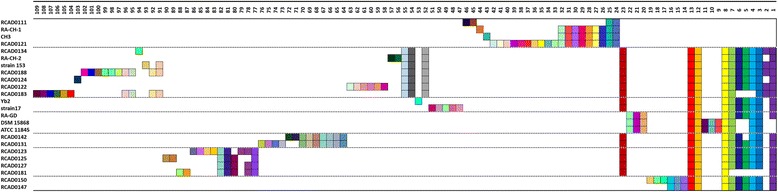
Spacer organization in CRISPR1. The CRISPR1 arrays from 24 *R. anatipestifer* strains are graphic represented. In order to analyze conveniently, the repeats have been eliminated and only the spacers are shown. The different subgroups are separated by the dotted lines. All arrays in the same subgroup are aligned manually. The abscissa indicates serial numbers of spacers, and ordinate displays names of analyzed *R. anatipestifer* strains. The direction of spacers is consistent with serial numbers (ie spacer 1 is located in 5’ terminal). Identical spacers are displayed by the same color, and are aligned such that they have the same number (apart from duplicate spacer). Additionally, unique spacer is displayed by a unique combination of color and number

**Fig. 6:**
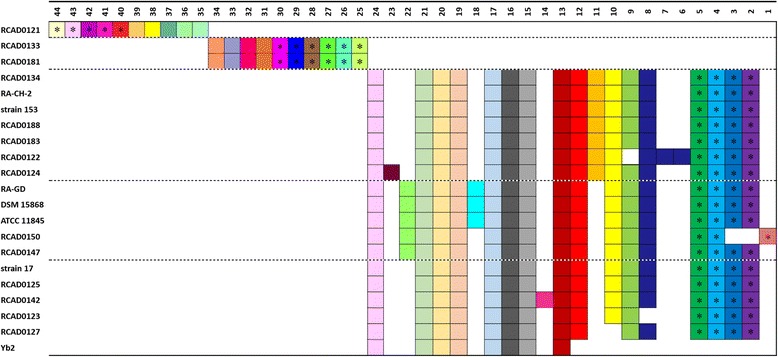
Spacer organization in CRISPR2. The CRISPR2 arrays from 21 *R. anatipestifer* strains are graphic represented as in Fig. 5. The asterisk represents the spacer with large size

Synthetically considering the similarity of the CRISPR arrays and the phylogeny of the strains (Fig. 4), CRISPR1 arrays can be divided into seven subgroups, and CRISPR2 arrays into five subgroups. And then we analyzed spacer organization in the same subgroup. The CRISPR1 and CRISPR2 loci with repeats eliminated are indicated in Figs. 5 and 6.

Of the CRISPR loci that were analyzed as part of this study, 153 different spacers were observed. On average, the number of spacers observed in a locus was 14 and 15 for CRISPR1 and CRISPR2, respectively. The CRISPR1 spacers were observed to be 29–30 nt in length, while the CRISPR2 spacers were 29–82 nt (Additional file 1: Table S2). Apart from Yb2, the CRISPR2 arrays possessed spacers with extreme sizes occurred in all CRISPR2-containing strains. Interestingly, distinct 3–6 CRISPR2 spacers with large size usually localized in terminal of the array in succession (Fig. 6).

Spacer loss occurred in both CRISPR1 and CRISPR2 (Figs. 5 and 6). Moreover, the majority of disparities associated with CRISPR2 arrays resulted from the loss of one or more spacers. Spacer loss was observed to occur in two different formats: loss of two or more successive spacers (eg in CRISPR1 of RCAD0111, RCAD0183 and RCAD0181; in CRISPR2 of RCAD0150 and Yb2), and discontinuous loss (eg in CRISPR1 of RCAD0142, RCAD0123 and RCAD0125; in CRISPR2 of RCAD0122 and RCAD0123).

Given the mechanisms that underpin spacer acquisition, the spacers in a CRISPR array differ from each other. However, there is an exception to this occurrence. On occasion, two or more tandem spacers are identical because of duplication events. Duplication of spacers was observed in both CRISPR1 and CRISPR2 (Figs. 5 and 6). Spacer duplication resulted in either a singularly copied unit (CRISPR1 spacer 2 of RCAD0134, strain 153, RCAD0188, RCAD0122, and RCAD0183; CRISPR1 spacer 78 of RCAD0123), or a single spacer duplicated multiple times (CRISPR2 spacer 8 of RCAD0122).

As part of this analysis, we observed the presence of unique spacers that only occur in individual strains in the same subgroup. Sixty-three unique spacers were found in the CRISPR loci that were analyzed in total, 60 and 3 for CRISPR1 and CRISPR2, respectively (Figs. 5 and 6). Unique CRISPR2 spacers were present in RCAD0124 (spacer 23), RCAD0150 (spacer 1) and RCAD0142 (spacer 14). Each of these spacers was composed of a unique nucleotide sequence. In CRISPR1, we observed 60 unique spacers, characterized by the presence of either two or more contiguous spacers (eg spacer 45–46 of RCAD0111 and spacer 33–42 of RCAD0121; spacer 56–57 of RA-CH-2, spacer 97–102 of RCAD0188, spacer 58–63 of RCAD0122 and spacer 104–109 of RCAD0183; spacer 47–51 of strain 17; spacer 71–72 of RCAD0142 and spacer 73–76 of RCAD0131; spacer 83–86 of RCAD0123, spacer 89–90 of RCAD0125 and spacer 87–88 of RCAD0181; spacer 17–19 of RCAD0150), or a single sequence (eg spacer 44 of RA-CH-1 and spacer 43 of CH3; spacer 94 of RCAD0134, spacer 93 of strain 153 and spacer 103 of RCAD0124; spacer 53 of Yb2). The majority of the unique spacers were located at the leader-proximal terminus. This would suggest that these spacers were derived following spacer acquisition.

Interestingly, the spacer organization of RCAD0133 CRISPR2 was identical to RCAD0181, Additionally, the same case was observed in RCAD0134, RA-CH-2, strain 153, RCAD0188 and RCAD0183; RA-GD, DSM15868 and ATCC11845; strain 17 and RCAD0125 (Fig. 6). Conversely, this phenomenon was rare in CRISPR1, ie, only the spacer organization of DSM15868 CRISPR1 and that of ATCC11845 were identical (Fig. 5). This finding was in agreement with the number of unique spacers of the two loci. Overall, the spacer organization of CRISPR1 was more diverse than that of CRISPR2.

Furthermore, the spacer organization was varied from subgroup to subgroup. Combined with analysis of the phylogenetic tree of these strains (Fig. 4), we found the subgroups of CRISPR1 spacer organization could be correlated roughly with the clusters of the phylogenetic tree (Figs. 4 and 5), while this connection was weak in CRISPR2 (Figs. 4 and 6). The subgroups of spacer organization in CRISPR1 was not completely consistent with phylogeny on account of some exceptions as follow: first, from the phylogenetic tree, it was clear to see RCAD0133 was closely related to RCAD0121 which possessed CRISPR1 locus, but it did not harbor the locus; second, the spacer organization was similar in RCAD0125, RCAD0123, RCAD0181 and RCAD0127, but the four strains were distributed in distinct clades; third, RCAD0124, RCAD0122, RCAD0183 and Yb2 were clustered in the phylogenetic tree, while their spacer organization were not grouped in the same subgroup, the same observation was made for strain 17 and RA-GD.

### Identification of phage/plasmid protospacers

Given that spacers play a significant role in defense mechanisms that are elicited against cognate foreign nucleic acids (ie bacteriophages and plasmids), we next analyzed whether *R. anatipestifer* spacers contained homology with phage or plasmid sequences. CRISPRTarget was used to identify putative protospacers [40], and hits that showed ≥87 % identity (≥26/30 nt) were deemed to be significant. As mentioned above, 153 spacers were identified in the 45 loci that were analyzed. Of these, only 8 % (13/153) of the spacer were similar to known phage or plasmid nucleotide sequences. Twelve of these thirteen spacers were located in CRISPR1 (Table 1). The putative protospacers were present in the genomes of *Enterobacteria* phage phi92, *Riemerella* phage RAP44 and plasmids of *Azospirillum brasilense*, *Deferribacter desulfuricans* and *Pantoea sp*. At-9b. The sequences that showed homology with *Riemerella* phage RAP44 usually displayed a perfect match, with more than one match being observed in one locus (eg spacer 80 and 89 of RCAD0125 CRISPR1; spacer 34, 36 and 38 of RCAD0121 CRISPR1; spacer 80 and 88 of RCAD0181 CRISPR1) (Table 1 and Fig. 5). There were two putative protospacers with identical identity for spacer 20. The same observation was made for spacer 48.

**Table 1:** Identification of phage/plasmid protospacers

Spacer Number	Description of protospacer	Position of protospacer	Similarity
^1^6	*Enterobacteria* phage phi92	146275-146304	87 %
^1^16	*Riemerella* phage RAP44	42519–42548	100 %
^1^20	*Riemerella* phage RAP44/*Deferribacter desulfuricans* SSM1 plasmid megaplasmid pDF308 DNA	42842–42871/80620–80649	87 %/87 %
^1^34	*Riemerella* phage RAP44	8954–8983	100 %
^1^36	*Riemerella* phage RAP44	27500–27529	90 %
^1^38	*Riemerella* phage RAP44	15935–15964	100 %
^1^45	*Riemerella* phage RAP44	13831–13860	100 %
^1^48	*Azospirillum brasilense* strain Az39 plasmid AbAZ39_p4/*Azospirillum brasilense* Sp245 plasmid AZOBR_p4	213466–213495/224417–224446	87 %/87 %
^1^55	*Riemerella* phage RAP44	24539–24568	100 %
^1^80	*Riemerella* phage RAP44	28468–28497	97 %
^1^88	*Riemerella* phage RAP44	1300–1329	100 %
^1^89	*Riemerella* phage RAP44	2509–2538	97 %
^1^95	*Pantoea* sp. At-9b plasmid pPAT9B01	317614–317642	93 %
^2^38	*Riemerella* phage RAP44	32595–32623	97 %

The superscript “1” represents CRISPR1, and “2” represents CRISPR2. The spacer number is corresponding to that in Figs. 5 and 6

**Fig. 7:**
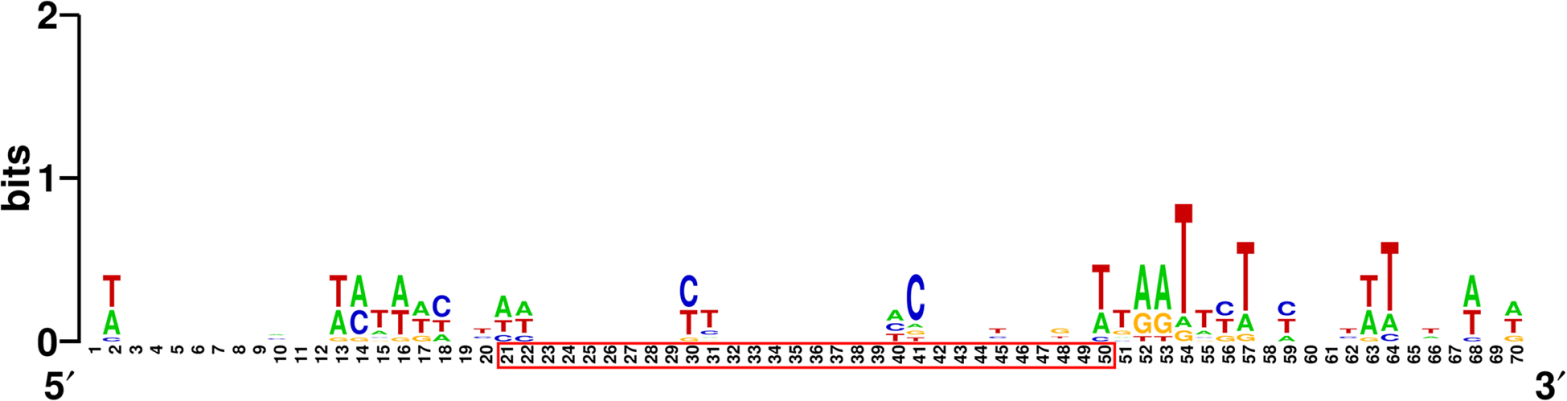
Prediction of PAM consensus sequence. The numbers associated with protospacer sequence are framed by red box. The height of the letters indicates the relative frequency of the corresponding base at that position

We also analyzed whether the putative protospacers displayed conserved PAM sequences. The protospacers with flanking sequence (20 bp on each side) were extracted and aligned. Unfortunately, whether in 5’ terminal or 3’ terminal, there was no obviously conserved motif. The T at 54th position was relatively conserved (Fig. 7).

### Characteristics of the *cas* operon

As previously mentioned, only three *cas* genes were observed upstream of CRISPR1, and no *cas* genes were found proximal to CRISPR2. Therefore, this analysis focused on the *cas1*, *cas2,* and *cas9* genes that adjacent to CRISPR1. Previous reports have published analysis of the *cas1* [50] and *cas2* [51] genes in the *R. anatipestifer* reference strain, ATCC 11845. In this study, we performed an extensive analysis on the *cas* operon of all of the analyzed *R. anatipestifer* strains.

The *cas* operon was composed of *cas9*, *cas1* and *cas2*. This was consistent with a type-II system (subtype C) [47] (Fig. 1). There was a 12 bp-long interval between *cas9* and *cas1*, while *cas1* and *cas2* had an 11 bp-long overlap. The length of the *cas1* gene was observed to be identical in all of the strains analyzed. The same observation was made for *cas2.* On average, the size of predicted ORFs for *cas1* genes was 897 bp and the GC content was 36 %. The *cas1* gene was predicted to translate a protein composed of 299 amino acids. The predicted ORF for *cas2* was 336 bp in length and the GC content was 36 %. The putative protein encoded by *cas2* constituted a protein of 112 amino acids. Cas1 and Cas2 are the core proteins of the CRISPR-Cas system, and both are conserved in one species [5]. To determine whether the two Cas proteins are conserved in the *R. anatipestifer* strains, we performed multiple sequence alignments with the predicted amino acid sequences for Cas1 and Cas2, respectively. The results indicated that Cas2 was identical in the *R. anatipestifer* strains that were analyzed (Additional file 1: Figure S1). Cas1 proteins can be divided into two groups with identical sequences. The Cas1 of the four strains (RA-CH-1, CH3, RCAD0111 and RCAD0121) were the same, and the rest of the strains had identical amino acid sequence for Cas1. But in general, Cas1 was still remarkably similar in these analyzed strains (Additional file 1: Figure S2).

Differences in the *cas* operon predominantly occurred in the *cas9* gene. The majority of *cas9* genes were 4200 bp in length, and encoded proteins composed of 1400 amino acids. However, exceptions existed. For example, the *cas9* gene of RA-CH-1, CH3, and RCAD0121 was 4215 bp. This gene translated into a protein of 1405 amino acids. Additionally, the RCAD0111 *cas9* gene was 4227 bp and the associated translation product was 1409 amino acids sequence. Similar to the observations pertaining to Cas1, diversity in Cas9 predicted amino acid sequences occurred in the four strains, ie RA-CH-1, CH3, RCAD0111 and RCAD0121 (Additional file 1: Figure S3). Although multiple sequence alignments associated with this protein indicated that there was so much variation in the four strains that were mentioned above, intact HNH and RuvC-like domains with conserved catalytic sites were present in all of the analyzed Cas9 protein sequences (Additional file 1: Figure S3).

**Fig. 8:**
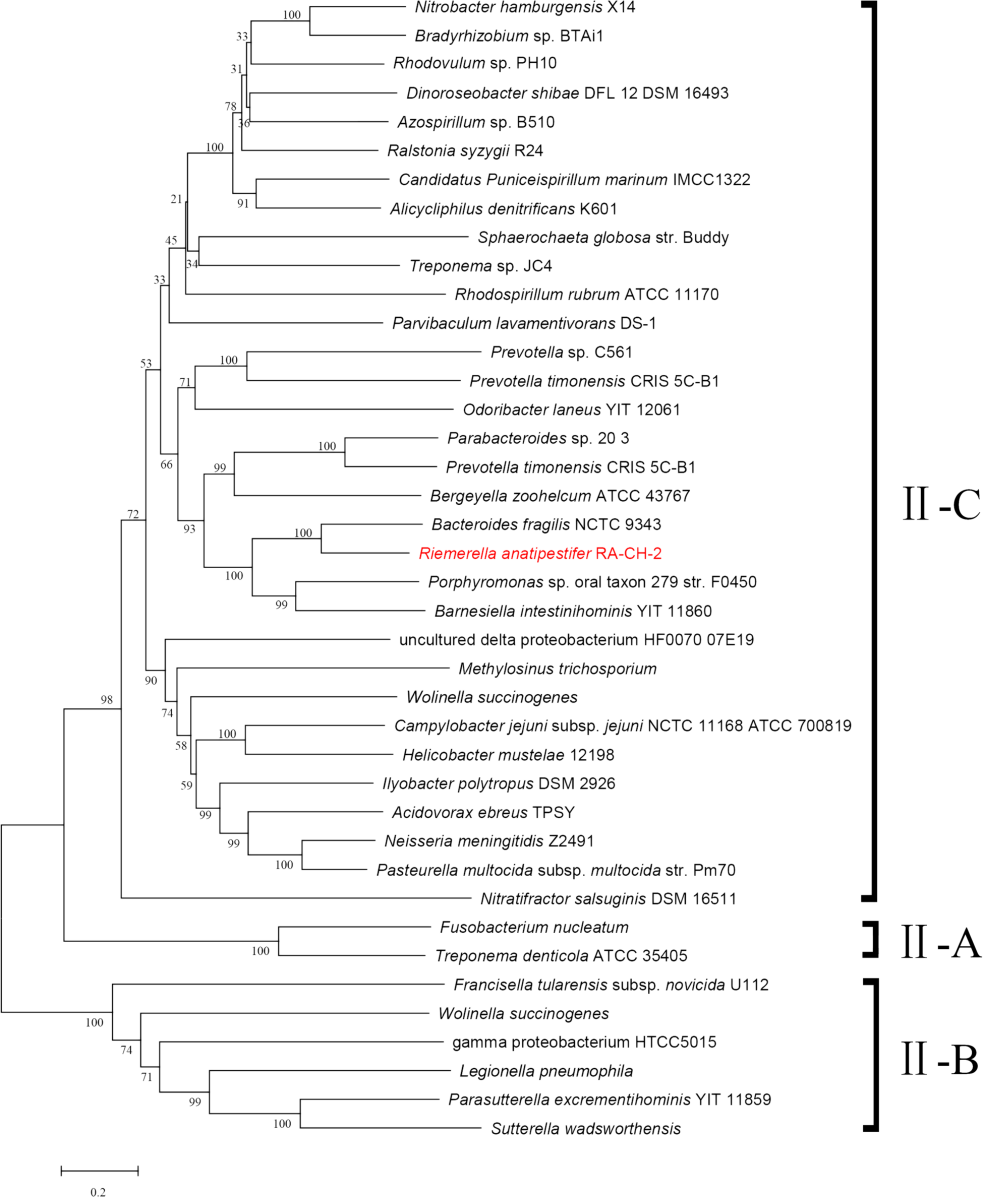
Phylogenetic tree for Cas9 from 40 Gram-negative type II system-containing bacteria. Cas9 proteins from 39 Gram-negative type II system-containing bacteria were selected for analysis (see Table S3). A phylogenetic tree based on Cas9 proteins from RA-CH-2 and 39 Gram-negative type II system-containing bacteria was constructed by neighbor-joining method using MEGA software (version 6). The bootstrap values of each node are shown. Different subclusters are enclosed by distinct brackets, and is in accordance with subtypes of type II system. The *R. anatipestifer* strain, RA-CH-2, is in red

It has been reported that a phylogenetic analysis of Cas9 in 75 representative type II systems is consistent with the classification of type II systems characterized to date [25]. In order to observe the evolutionary relationship of Cas9 protein sequences between *R. anatipestifer* and other type II system-containing strains and determine the type of *R. anatipestifer* CRISPR-Cas system, we selected 39 Cas9 amino acid sequences from Gram-negative type II system-containing bacteria and RA-CH-2 for analysis. Next, we constructed a multiple sequence alignment and a phylogenetic tree for Cas9 (Additional file 1: Table S3, Figure S4, and Fig. 8). The phylogenetic tree (Fig. 8) showed that the Cas9 sequence from *R. anatipestifer* was closely related to that of *Bacteroides fragilis* NCTC 9343 and formed part of the subtype II-C subcluster.

## Discussion

In this study, the structural characteristics and components associated with *R. anatipestifer* CRISPR-Cas systems were analyzed. Based on previous studies, we observed that the CRISPR1-*cas* locus in *R. anatipestifer* is highly similar to subtype II-C. Firstly, the CRISPR1-*cas* locus possessed the minimal number of *cas* genes required to formulate the *cas* operon, which is a characteristic of subtype II-C [30]. Secondly, repeats of type II systems are predominantly 36 nt in length, while the repeats of *R. anatipestifer* CRISPR1 were 47 nt. This is in agreement with the finding that unusually long repeats (up to 48 nt) are exclusively present in type II-C systems, especially in the *Bacteroidetes* phylum [25, 47]. Additionally, the *R. anatipestifer* Cas9 protein was obviously a member of the subtype II-C subcluster (as borne out by the phylogenetic tree). These observations suggest that the CRISPR1-Cas system of *R. anatipestifer* is a type II-C system.

Compared with the relatively conserved repeats and *cas* genes, the spacer organization in *R. anatipestifer* was more diverse as a consequence of spacer acquisition. In CRISPR1, new spacers were typically inserted into the putative leader sequence-proximal end. This occurrence resulted in older and more conserved spacers being located in the leader-distal end, and numerous unique spacers were located in the leader-proximal end. By contrast, the spacer organization in CRISPR2 appears to be more stable. By inference, the CRISPR2 loci of *R. anatipestifer* are likely to have lost adaptive abilities during evolution. Although the reasons that have led to the occurrence of this phenomenon are currently unknown, similar observations have been made in *Salmonella* [52]. Intriguingly, the spacer organization of CRISPR arrays were not completely in agreement with the phylogenetic analysis of analyzed *R. anatipestifer* strains, suggesting that horizontal gene transfer (HGT) associated with CRISPR loci among *R. anatipestifer* strains had occurred.

Interestingly, the four strains (RCAD0111, RA-CH-1, CH3 and RCAD0121) were distinguished from other analyzed strains in putative leader sequence and amino acid sequences of Cas1 and Cas9. This finding is consistent with the phylogeny of *R. anatipestifer* strains, namely, the four strains were clustered in the phylogenetic tree of *R. anatipestifer* strains. However, we cannot explain why there were minor variations and whether these variations impact biological activity of these elements at present. Nevertheless, two distinct endonuclease domains – HNH and RuvC-like, which both are essential for the cleavage function of Cas9 [21, 53] were present in each analyzed Cas9 protein sequence, though there was so much variation in the sequences.

The tracrRNA is critical for processing and interference activities associated with type II systems [25, 54]. The repeat portion of pre-crRNA and the anti-repeat portion of tracrRNA pair up to form tracrRNA:pre-crRNA duplex in the presence of Cas9, and then the duplex is co-processed by the host factor RNase III. The mature crRNA (maintains in a complex with tracrRNA) guides Cas9 to cleavage target DNA during the interference step [20, 21, 54]. However, the tracrRNA is difficult to identify due to the diversity of sequence length, content, structure and localization [20, 25]. Therefore, we can only predict the anti-repeat sequence of tracrRNA-encoding DNA by analyzing the conservative form of base-pairing that is utilized by these repeat sequences. Upstream of the *cas* operon, where CRISPR1 was located, there was a 16 nt-long anti-repeat sequence, which might be regarded as a portion of the sequence that encodes for a putative tracrRNA. As mentioned above, the repeat sequence was comprised of 47 nucleotides, so the mismatches in repeat sequence may be combined with other factors or the 16 nt-long sequence is just sufficient for following activities. Likewise, the mismatches in tracrRNA-encoding sequence may be bound to other factors or form secondary structure to maintain structural stability. Given the complementarity between pre-crRNA and tracrRNA, the repeat sequences of the type II system are weakly palindromic. In other words, the repeat itself is not likely to form stem-loop structure that is deemed essential to pre-crRNA maturation in type I and III systems [20, 47]. Intriguingly, the repeat sequences in *R. anatipestifer* showed ordinary stem-loop structures, though there were some differences in the secondary structures resulting from DRVs.

However, the molecular mechanisms elicited by subtype II-C are likely to be different from those that underpin type II systems. It has been confirmed that a streamlined type II-C system occurred in *Neisseria meningitides* [55]. In this system, the CRISPR locus was devoid of identifiable leader sequences containing external promoters, but possessed functional promoters embedded within each repeat. Moreover, RNase III-mediated processing was dispensable due to immediate transcription of short crRNAs from internal promoters in the repeats. Conversely, potential promoters were not found in the repeats of *R. anatipestifer* CRISPR1. Additionally, we predicted (with high scores) the occurrence of 107 bp-long leader sequences containing several putative promoters upstream of the CRISPR1 arrays. Palindromic sequences were also found in the putative leaders. Taken together, we speculate that the pre-crRNA processing pathway of the *R. anatipestifer* type II-C system is distinguishable from that of *N. meningitides.* Further analysis of RNA-seq is required to provide a more detailed insight into the actual molecular mechanism that underpins the type II-C system in *R. anatipestifer*. The CRISPR-Cas system is an adaptive immune system in prokaryotes that promotes the protection of bacteria and archaea from invasion by phages and plasmids [14–16]. Thus, we identified putative protospacers homologous with the spacers of *R. anatipestifer*. The only phage to share complete identity with the analyzed spacers was the phage of *R. anatipestifer* (RAP44) [56]. This suggests that the *R. anatipestifer* strains which contain the spacers homologous to RAP44 were likely infected by a phage related to RAP44, or a common ancestor have been attacked by the phage and transferred the spacer to its descendants. Therefore, these strains are likely resistant to the phage related to RAP44, and we need to identify the biological activity by experiment. It is possible that the spacers harbor identity with other *R. anatipestifer* bacteriophages, but many of these bacteriophages have not yet been characterized. This is likely because CRISPR-Cas system-mediated immunity leads to difficult isolation of *R. anatipestifer* phages. Other analyzed spacers matched foreign fragments derived from plasmids of *Azospirillum brasilense*, *Deferribacter desulfuricans* and *Pantoea sp.* At-9b, suggesting that *R. anatipestifer* might has hosted to these genetic elements or similar elements. Unfortunately, we found no consensus sequence associated with PAM in silico, which perhaps resulted from insufficient putative protospacers, and we may be able to discover functional PAM via experimental measures [57].

## Conclusions

We have identified the structural characteristics and components of *R. anatipestifer* CRISPR-Cas system with comparative genomics analysis. The CRISPR1-Cas system of *R. anatipestifer* can be classified as subtype II-C, while the neighborless CRISPR2 with smaller variance could be inactive. The polymorphism of spacer organization may reflect the process of adaptation in *R. anatipestifer*. The features of the components associated with CRISPR1-Cas system suggest a distinct molecular mechanism utilized by *R. anatipestifer*. This research may assist in studying the specific mechanism associated with CRISPR-mediated adaptive immunity and other biological functions in *R. anatipestifer*.

## Additional files

Additional file 1: Table S1. The origin and accession number of 25 *R. anatipestifer* genomes. Table S2. The CRISPR loci in *R. anatipestifer* genomes. Table S3. Cas9-related information for 39 Gram-negative type II system-containing bacteria. Figure S1. Multiple sequence alignment of Cas2 amino acid sequences. The asterisk represents the conserved base in all of the sequences for alignment. Figure S2. Multiple sequence alignment of Cas1 amino acid sequences. The asterisk represents the conserved base in all of the sequences for alignment. Figure S3. Multiple sequence alignment of Cas9 amino acid sequences. The asterisk represents the conserved base in all of the sequences for alignment. Based on previous report (Chylinski K, et al., [47]), we defined that the amino acid sequence located between 1 and 60 aa, 718–775 aa and 910–1131 aa are RuvC-like domains (black boxes). The amino acid sequence located between 776 and 909 aa is considered as HNH domain (red box). The yellow shadows indicate catalytic sites. Figure S4. Multiple sequence alignment for Cas9 from 40 Gram-negative type II system-containing bacteria. ClustalW (in MEGA software) was utilized to perform multiple sequence alignment for Cas9 from 40 Gram-negative type II system-containing bacteria. The default parameter values were chosen when computed. (DOCX 2633 kb)

## Abbreviations

A/T: Adenine/thymine
bp: Base pair
*cas* genes: CRISPR-associated genes
Cas proteins: CRISPR-associated proteins
CRISPR: Clustered regularly interspaced short palindromic repeats
crRNA: CRISPR RNA
DRV: Direct repeat variant
GC content: Guanine-cytosine content
HGT: Horizontal gene transfer
NCBI: National Center for Biotechnology Information
nt: Nucleotide
ORF: Open reading frame
PAM: Protospacer adjacent motif
PGAP: Prokaryotic genome annotation pipeline
SNP: Single-nucleotide polymorphism
tracrRNA: *trans*-encoded CRISPR RNA

## References

[1] Ruiz JA, Sandhu TS, Swayne DE, Glisson JR, McDougald LR, Nolan LK, Suarez DL, Nair VL (2013). *Riemerella anatipestifer* Infection. Diseases of poultry.

[2] Marraffini LA, Sontheimer EJ (2010). CRISPR interference: RNA-directed adaptive immunity in bacteria and archaea. Nat Rev Genet.

[3] Barrangou R, Marraffini LA (2014). CRISPR-Cas systems: Prokaryotes upgrade to adaptive immunity. Mol Cell.

[4] Barrangou R (2015). The roles of CRISPR–Cas systems in adaptive immunity and beyond. Curr Opin Immunol.

[5] Makarova KS, Haft DH, Barrangou R, Brouns SJ, Charpentier E, Horvath P, Moineau S, Mojica FJ, Wolf YI, Yakunin AF (2011). Evolution and classification of the CRISPR-Cas systems. Nat Rev Microbiol.

[6] Makarova KS, Wolf YI, Koonin EV (2013). Comparative genomics of defense systems in archaea and bacteria. Nucleic Acids Res.

[7] Barrangou R, Fremaux C, Deveau H, Richards M, Boyaval P, Moineau S, Romero DA, Horvath P (2007). CRISPR provides acquired resistance against viruses in prokaryotes. Science.

[8] Wei Y, Chesne MT, Terns RM, Terns MP (2015). Sequences spanning the leader-repeat junction mediate CRISPR adaptation to phage in *Streptococcus thermophilus*. Nucleic Acids Res.

[9] He L, Fan X, Xie J (2012). Comparative genomic structures of *Mycobacterium* CRISPR-Cas. J Cell Biochem.

[10] Peng L, Pei J, Hao P, Yuan G, Lin L, Huang R (2014). Whole genome sequencing reveals a novel CRISPR system in industrial *Clostridium acetobutylicum*. J Ind Microbiol Biotechnol.

[11] Pul U, Wurm RZ, Geissen R, Hofmann N, Wagner R (2010). Identification and characterization of *E. coli* CRISPR-cas promoters and their silencing by H-NS. Mol Microbiol.

[12] Bolotin A, Quinquis B, Sorokin A, Ehrlich SD (2005). Clustered regularly interspaced short palindrome repeats (CRISPRs) have spacers of extrachromosomal origin. Microbiology.

[13] Mojica FJM, Díez-Villaseñor C, García-Martínez J, Soria E (2005). Intervening sequences of regularly spaced prokaryotic repeats derive from foreign genetic elements. J Mol Evol.

[14] Hooton SP, Brathwaite KJ, Connerton IF (2016). The bacteriophage carrier state of *campylobacter jejuni* features changes in host Non-coding RNAs and the acquisition of New host-derived CRISPR spacer sequences. Front Microbiol.

[15] Patterson AG, Chang JT, Taylor C, Fineran PC (2015). Regulation of the Type I-F CRISPR-Cas system by CRP-cAMP and GalM controls spacer acquisition and interference. Nucleic Acids Res.

[16] Hooton SP, Connerton IF (2014). Campylobacter jejuni acquire new host-derived CRISPR spacers when in association with bacteriophages harboring a CRISPR-like Cas4 protein. Front Microbiol.

[17] Yosef I, Goren MG, Qimron U (2012). Proteins and DNA elements essential for the CRISPR adaptation process in *Escherichia coli*. Nucleic Acids Res.

[18] Jason C, Christopher RT, Smith JT, Sara O, Rodolphe B, Sylvain M, Glover CVC, Graveley BR, Terns RM, Terns MP (2014). The three major types of CRISPR-Cas systems function independently in CRISPR RNA biogenesis in *Streptococcus thermophilus*. Mol Microbiol.

[19] Wang R, Preamplume G, Terns MP, Terns RM, Li H (2011). Interaction of the Cas6 riboendonuclease with CRISPR RNAs: recognition and cleavage. Structure.

[20] Deltcheva E, Chylinski K, Sharma CM, Gonzales K, Chao Y, Pirzada ZA, Eckert MR, Vogel J, Charpentier E (2011). CRISPR RNA maturation by trans-encoded small RNA and host factor RNase III. Nature.

[21] Garneau JE, Dupuis ME, Villion M, Romero DA, Barrangou R, Boyaval P, Fremaux C, Horvath P, Magadan AH, Moineau S (2010). The CRISPR/Cas bacterial immune system cleaves bacteriophage and plasmid DNA. Nature.

[22] Magadán AH, Dupuis M, Villion M, Moineau S (2012). Cleavage of Phage DNA by the Streptococcus thermophilus CRISPR3-Cas System. PLoS One.

[23] Jinek M, Chylinski K, Fonfara I, Hauer M, Doudna JA, Charpentier E (2012). A programmable dual-RNA-guided DNA endonuclease in adaptive bacterial immunity. Science.

[24] Samai P, Pyenson N, Jiang W, Goldberg GW, Hatoum-Aslan A, Marraffini LA (2015). Co-transcriptional DNA and RNA Cleavage during Type III CRISPR-Cas Immunity. Cell.

[25] Chylinski K, Le Rhun A, Charpentier E (2013). The tracrRNA and Cas9 families of type II CRISPR-Cas immunity systems. RNA Biol.

[26] Deveau H, Barrangou R, Garneau JE, Labonte J, Fremaux C, Boyaval P, Romero DA, Horvath P, Moineau S (2008). Phage response to CRISPR-encoded resistance in *Streptococcus thermophilus*. J Bacteriol.

[27] Mojica FJ, Diez-Villasenor C, Garcia-Martinez J, Almendros C (2009). Short motif sequences determine the targets of the prokaryotic CRISPR defence system. Microbiology.

[28] Shah SA, Erdmann S, Mojica FJ, Garrett RA (2013). Protospacer recognition motifs: mixed identities and functional diversity. RNA Biol.

[29] Marraffini LA, Sontheimer EJ (2010). Self versus non-self discrimination during CRISPR RNA-directed immunity. Nature.

[30] Makarova KS, Wolf YI, Alkhnbashi OS, Costa F, Shah SA, Saunders SJ, Barrangou R, Brouns SJ, Charpentier E, Haft DH (2015). An updated evolutionary classification of CRISPR-Cas systems. Nat Rev Microbiol.

[31] Shmakov S, Abudayyeh O, Makarova K, Wolf Y, Gootenberg J, Semenova E, Minakhin L, Joung J, Konermann S, Severinov K (2015). Discovery and Functional Characterization of Diverse Class 2 CRISPR-Cas Systems. Mol Cell.

[32] Makarova KS, Wolf YI, Koonin EV (2013). The basic building blocks and evolution of CRISPR-CAS systems. Biochem Soc Trans.

[33] Benson D, Boguski M, Lipman D, Ostell J (1990). The National Center for Biotechnology Information. Genomics.

[34] Zerbino DR, Birney E (2008). Velvet: algorithms for *de novo* short read assembly using de Bruijn graphs. Genome Res.

[35] Chaudhari NM, Gupta VK, Dutta C. BPGA- an ultra-fast pan-genome analysis pipeline. Sci Rep. 2016;6. 10.1038/srep24373 PMC4829868 27071527

[36] Grissa I, Vergnaud G, Pourcel C (2007). The CRISPRdb database and tools to display CRISPRs and to generate dictionaries of spacers and repeats. BMC Bioinformatics.

[37] Rousseau C, Gonnet M, Le Romancer M, Nicolas J (2009). CRISPI: a CRISPR interactive database. Bioinformatics.

[38] Crooks GE, Hon G, Chandonia JM, Brenner SE (2004). WebLogo: a sequence logo generator. Genome Res.

[39] Zuker M (2003). Mfold web server for nucleic acid folding and hybridization prediction. Nucleic Acids Res.

[40] Biswas A, Gagnon JN, Brouns SJ, Fineran PC, Brown CM (2013). CRISPRTarget: bioinformatic prediction and analysis of crRNA targets. RNA Biol.

[41] Thompson JD, Gibson TJ, Higgins DG: Multiple sequence alignment using ClustalW and ClustalX. Curr Protoc Bioinformatics 2002, Chapter 2:Unit 2.3. 10.1002/0471250953.bi0203s00 18792934

[42] Reese MG (2001). Application of a time-delay neural network to promoter annotation in the *Drosophila melanogaster* genome. Comput Chem.

[43] Altschul SF, Gish W, Miller W, Myers EW, Lipman DJ (1990). Basic local alignment search tool. J Mol Biol.

[44] Kumar S, Nei M, Dudley J, Tamura K (2008). MEGA: a biologist-centric software for evolutionary analysis of DNA and protein sequences. Brief Bioinform.

[45] Kunin V, Sorek R, Hugenholtz P (2007). Evolutionary conservation of sequence and secondary structures in CRISPR repeats. Genome Biol.

[46] Godde JS, Bickerton A (2006). The repetitive DNA elements called CRISPRs and their associated genes: evidence of horizontal transfer among prokaryotes. J Mol Evol.

[47] Chylinski K, Makarova KS, Charpentier E, Koonin EV (2014). Classification and evolution of type II CRISPR-Cas systems. Nucleic Acids Res.

[48] Datsenko KA, Pougach K, Tikhonov A, Wanner BL, Severinov K, Semenova E (2012). Molecular memory of prior infections activates the CRISPR/Cas adaptive bacterial immunity system. Nat Commun.

[49] Diez-Villasenor C, Guzman NM, Almendros C, Garcia-Martinez J, Mojica FJ (2013). CRISPR-spacer integration reporter plasmids reveal distinct genuine acquisition specificities among CRISPR-Cas I-E variants of *Escherichia coli*. RNA Biol.

[50] He Y, Cheng AC, Wang MS, Zhu DK, Wang XJ, Zhang X (2013). Sequence Analysis of the Cas1 Gene in *Riemerella anatipestifer*. Adv Mater Res.

[51] He Y, Cheng AC, Wang MS, Zhu DK, Wang XJ, Zhang X (2013). Sequence Analysis of the Cas2 Gene in *Riemerella anatipestifer*. Adv Mater Res.

[52] Shariat N, Timme RE, Pettengill JB, Barrangou R, Dudley EG (2015). Characterization and evolution of *Salmonella* CRISPR-Cas systems. Microbiology.

[53] Nishimasu H, Ran FA, Hsu PD, Konermann S, Shehata SI, Dohmae N, Ishitani R, Zhang F, Nureki O (2014). Crystal structure of Cas9 in complex with guide RNA and target DNA. Cell.

[54] Karvelis T, Gasiunas G, Miksys A, Barrangou R, Horvath P, Siksnys V (2013). crRNA and tracrRNA guide Cas9-mediated DNA interference in *Streptococcus thermophilus*. RNA Biol.

[55] Zhang Y, Heidrich N, Ampattu BJ, Gunderson CW, Seifert HS, Schoen C, Vogel J, Sontheimer EJ (2013). Processing-independent CRISPR RNAs limit natural transformation in *Neisseria meningitidis*. Mol Cell.

[56] Cheng LF, Chen HM, Zheng T, Fu GH, Shi SH, Wan CH, Huang Y (2012). Complete genomic sequence of the virulent bacteriophage RAP44 of *Riemerella anatipestifer*. Avian Dis.

[57] Leenay RT, Maksimchuk KR, Slotkowski RA, Agrawal RN, Gomaa AA, Briner AE, Barrangou R, Beisel CL (2016). Identifying and Visualizing Functional PAM Diversity across CRISPR-Cas Systems. Mol Cell.

