# Supplementary material for: Comparative genomic analysis identifies structural features of CRISPR-Cas systems in Riemerella anatipestifer
Source: BMC Genomics. 2016 Aug 30;17(1):689. doi: 10.1186/s12864-016-3040-4 (PMC5006608; doi:10.1186/s12864-016-3040-4)
Supplement: Additional file 1: Table S1. — The origin and accession number of 25 R. anatipestifer genomes. Table S2. The CRISPR loci in R. anatipestifer genomes. Table S3. Cas9-related information for 39 Gram-negative type II system-containing bacteria. Figure S1. Multiple sequence alignment of Cas2 amino acid sequences. The asterisk represents the conserved base in all of the sequences for alignment. Figure S2. Multiple sequence alignment of Cas1 amino acid sequences. The asterisk represents the conserved base in all of the sequences for alignment. Figure S3. Multiple sequence alignment of Cas9 amino acid sequences. The asterisk represents the conserved base in all of the sequences for alignment. Based on previous report (Chylinski K, et al., [47]), we defined that the amino acid sequence located between 1 and 60 aa, 718–775 aa and 910–1131 aa are RuvC-like domains (black boxes). The amino acid sequence located between 776 and 909 aa is considered as HNH domain (red box). The yellow shadows indicate catalytic sites. Figure S4. Multiple sequence alignment for Cas9 from 40 Gram-negative type II system-containing bacteria. ClustalW (in MEGA software) was utilized to perform multiple sequence alignment for Cas9 from 40 Gram-negative type II system-containing bacteria. The default parameter values were chosen when computed. (DOCX 2633 kb) [file 12864_2016_3040_MOESM1_ESM.docx]

**Additional file 1: Table S1. The origin and accession number of 25 *R. anatipestifer* genomes**

| Name | Isolated date | Isolated location | Host | Length (bp) | Accession number |
| --- | --- | --- | --- | --- | --- |
| Yb2^[1]^ | ND | China | Duck | 2184066 | CP007204.1 |
| strain 153 | ND | China | Duck | 2160281 | CP007504.1 |
| strain 17 | ND | China | Duck | 2152519 | CP007503.1 |
| RA-GD^[2]^ | ND | China | Duck | 2166384 | CP002562.1 |
| RA-CH-2^[3]^ | 1996 | China | Duck | 2166321 | CP004020.1 |
| RA-CH-1^[3]^ | 1993 | China | Duck | 2309519 | CP003787.1 |
| DSM 15868^[4]^ | 1932 | USA | Duck | 2155121 | CP002346.1 |
| ATCC 11845 ^[5]^ | 1932 | USA | Duck | 2164087 | CP003388.1 |
| CH3^[6]^ | ND | China | Duck | 2234477 | CP006649.1 |
| RCAD0122 | 2012 | China | Duck | 2205797 | LUDU00000000 |
| RCAD0131 | 2011 | China | Duck | 2110744 | LUDS00000000 |
| RCAD0111 | 2012 | China | Duck | 2362938 | LUDR00000000 |
| RCAD0125 | 2012 | China | Duck | 2143994 | LUDJ00000000 |
| RCAD0127 | 2013 | China | Duck | 2093347 | LUDV00000000 |
| RCAD0134 | 2011 | China | Duck | 2158756 | LUDO00000000 |
| RCAD0142 | 2011 | China | Duck | 2109496 | LUDG00000000 |
| RCAD0188 | 2014 | China | Duck | 2162414 | LUDH00000000 |
| RCAD0133 | 2011 | China | Duck | 2437484 | LUDT00000000 |
| RCAD0124 | 2011 | China | Duck | 2198894 | LUDQ00000000 |
| RCAD0121 | 2011 | China | Duck | 2216702 | LUDI00000000 |
| RCAD0183 | 2014 | China | Duck | 2204034 | LUDK00000000 |
| RCAD0150 | 2011 | China | Duck | 2098894 | LUDM00000000 |
| RCAD0181 | 2014 | China | Duck | 2115418 | LUDL00000000 |
| RCAD0147 | 2011 | China | Duck | 2098201 | LUDN00000000 |
| RCAD0123 | 2011 | China | Duck | 2086211 | LUDP00000000 |

ND: Not Determined

**Additional file 2: Table S2. The CRISPR loci in *R. anatipestifer* genomes**

| Name | Number of  CRISPR loci | Confirmed CRISPR1 loci | | | | | | Confirmed CRISPR2 loci | | | | | |
| --- | --- | --- | --- | --- | --- | --- | --- | --- | --- | --- | --- | --- | --- |
|  |  | position in genome | Number of repeats | Repeat size (bp) | Number of spacers | Spacer size (bp) | Consensus direct repeat sequence (5’→3’) | position in genome | Number of repeats | Repeat size (bp) | Number of spacers | Spacer size (bp) | Consensus direct repeat sequence (5’→3’) |
| Yb2 | 2 | 879403-880296 | 12 | 47 | 11 | 30-30 | GTTGTGAATTGCTTTCAATTTTCACTATCTTTGTGATAATTCGCAAC | 1712504-1713058 | 9 | 36 | 8 | 29-30 | GTTGTGAATACCCTTCAAAATGAGAGCAGTCCCAAC |
| strain 153 | 2 | 858289-859566 | 17 | 47 | 16 | 29-30 | GTTGTGAATTGCTTTCAATTTTTACTATCTTTGTGATAGTTCGCAAC | 1731774-1733136 | 18 | 36 | 17 | 29-82 | GTTGTGAATACCCTTCAAAGTGAGAGCAGTCCCAAC |
| strain 17 | 2 | 846370-847571 | 16 | 47 | 15 | 30-30 | GTTGTGAATTGCTTTCAATTTTTACTATCTTTGTGATAGTTCGCAAC | 1685137-1686433 | 17 | 36 | 16 | 29-82 | GTTGTGAATACCCTTCAAAGTGAGAGCAGTCCCAAC |
| RA-GD | 2 | 1449546-1450516 | 13 | 47 | 12 | 30-30 | GTTGTGAATTGCTTTCAATTTTCACTATCTTTGTGATAATTCGCAAC | 623802-625231 | 19 | 36 | 18 | 29-82 | GTTGTGAATACCCTTCAAAATGAGAGCAGTCCCAAC |
| RA-CH-2 | 2 | 871223-872424 | 16 | 47 | 15 | 30-30 | GTTGTGAATTGCTTTCAATTTTTACTATCTTTGTGATAGTTCGCAAC | 2162272-2163634 | 18 | 36 | 17 | 29-82 | GTTGTGAATACCCTTCAAAGTGAGAGCAGTCCCAAC |
| RA-CH-1 | 1 | 1164086-1164901 | 11 | 47 | 10 | 29-30 | GTTGTGAATTGCTTTCAATTTTTACTATCTTTGTGATAGTTCGCAAC | - | - | - | - | - | - |
| DSM 15868 | 2 | 859687-860657 | 13 | 47 | 12 | 30-30 | GTTGTGAATTGCTTTCAATTTTTACTATCTTTGTGATAGTTCGCAAC | 1695512-1696941 | 19 | 36 | 18 | 29-82 | GTTGTGAATACCCTTCAAAATGAGAGCAGTCCCAAC |
| ATCC 11845 | 2 | 1020412-1021382 | 13 | 47 | 12 | 30-30 | GTTGTGAATTGCTTTCAATTTTTACTATCTTTGTGATAGTTCGCAAC | 1855428-1856857 | 19 | 36 | 18 | 29-82 | GTTGTGAATACCCTTCAAAATGAGAGCAGTCCCAAC |
| CH3 | 1 | 1594898-1595713 | 11 | 47 | 10 | 29-30 | GTTGTGAATTGCTTTCAATTTTTACTATCTTTGTGATAGTTCGCAAC | - | - | - | - | - | - |
| RCAD0122 | 2 | 651274-652860 | 21 | 47 | 20 | 30-30 | GTTGTGAATTGCTTTCAATTTTTACTATCTTTGTGATAGTTCGCAAC | 2132833-2134261 | 19 | 36 | 18 | 29-82 | GTTGTGAATACCCTTCAAAGTGAGAGCAGTCCCAAC |
| RCAD0131 | 1 | 1126197-1127859 | 22 | 47 | 21 | 29-30 | GTTGTGAATTGCTTTCAATTTTTACTATCTTTGTGATAGTTCGCAAC | - | - | - | - | - | - |
| RCAD0111 | 1 | 2166277-2166630 | 5 | 47 | 4 | 29-30 | GTTGTGAATTGCTTTCAATTTTTACTATCTTTGTGATAGTTCGCAAC | - | - | - | - | - | - |
| RCAD0125 | 2 | 315698-316976 | 17 | 47 | 16 | 30-30 | GTTGTGAATTGCTTTCAATTTTTACTATCTTTGTGATAGTTCGCAAC | 1214317-1215613 | 17 | 36 | 16 | 29-82 | GTTGTGAATACCCTTCAAAGTGAGAGCAGTCCCAAC |
| RCAD0127 | 2 | 1445275-1446476 | 16 | 47 | 15 | 30-30 | GTTGTGAATTGCTTTCAATTTTTACTATCTTTGTGATAGTTCGCAAC | 1072425-1073655 | 16 | 36 | 15 | 29-82 | GTTGTGAATACCCTTCAAAATGAGAGCAGTCCCAAC |
| RCAD0134 | 2 | 648863-650064 | 16 | 47 | 15 | 30-30 | GTTGTGAATTGCTTTCAATTTTTACTATCTTTGTGATAGTTCGCAAC | 2090397-2091759 | 18 | 36 | 17 | 29-82 | GTTGTGAATACCCTTCAAAGTGAGAGCAGTCCCAAC |
| RCAD0142 | 2 | 1197539-1198971 | 19 | 47 | 18 | 30-30 | GTTGTGAATTGCTTTCAATTTTTACTATCTTTGTGATAGTTCGCAAC | 1434199-1435561 | 18 | 36 | 17 | 29-82 | GTTGTGAATACCCTTCAAAATGAGAGCAGTCCCAAC |
| RCAD0188 | 2 | 1917463-1919356 | 25 | 47 | 24 | 29-30 | GTTGTGAATTGCTTTCAATTTTTACTATCTTTGTGATAGTTCGCAAC | 2089980-2091342 | 18 | 36 | 17 | 29-82 | GTTGTGAATACCCTTCAAAGTGAGAGCAGTCCCAAC |
| RCAD0133 | 1 | - | - | - | - | - | - | 2199729-2200729 | 11 | 36 | 10 | 30-81 | GTTGTGAATACCCTTCAAAATGAGAGTAGTCCCAAC |
| RCAD0124 | 2 | 648303-649427 | 15 | 47 | 14 | 30-30 | GTTGTGAATTGCTTTCAATTTTTACTATCTTTGTGATAGTTCGCAAC | 2138920-2140348 | 19 | 36 | 18 | 29-82 | GTTGTGAATACCCTTCAAAGTGAGAGCAGTCCCAAC |
| RCAD0121 | 2 | 2105112-2106620 | 20 | 47 | 19 | 29-30 | GTTGTGAATTGCTTTCAATTTTTACTATCTTTGTGATAGTTCGCAAC | 2050738-2051688 | 11 | 36 | 10 | 30-81 | GTTGTGAATACCCTTCAAAATGAGAGTAGTCCCAAC |
| RCAD0183 | 2 | 826436-828098 | 22 | 47 | 21 | 29-30 | GTTGTGAATTGCTTTCAATTTTTACTATCTTTGTGATAGTTCGCAAC | 2131579-2132941 | 18 | 36 | 17 | 29-82 | GTTGTGAATACCCTTCAAAGTGAGAGCAGTCCCAAC |
| RCAD0150 | 2 | 678262-679463 | 16 | 47 | 15 | 30-30 | GTTGTGAATTGCTTTCAATTTTTACTATCTTTGTGATAGTTCGCAAC | 1141754-1142935 | 17 | 36 | 16 | 29-81 | GTTGTGAATACCCTTCAAAATGAGAGCAGTCCCAAC |
| RCAD0181 | 2 | 826305-827275 | 13 | 47 | 12 | 30-30 | GTTGTGAATTGCTTTCAATTTTTACTATCTTTGTGATAGTTCGCAAC | 2027374-2028374 | 11 | 36 | 10 | 30-81 | GTTGTGAATACCCTTCAAAATGAGAGTAGTCCCAAC |
| RCAD0147 | 2 | 676890-677860 | 13 | 47 | 12 | 30-30 | GTTGTGAATTGCTTTCAATTTTCACTATCTTTGTGATAATTCGCAAC | 1138499-1139862 | 18 | 36 | 17 | 30-82 | GTTGTGAATACCCTTCAAAATGAGAGCAGTCCCAAC |
| RCAD0123 | 2 | 837145-838653 | 20 | 47 | 19 | 29-30 | GTTGTGAATTGCTTTCAATTTTTACTATCTTTGTGATAGTTCGCAAC | 1240275-1241505 | 16 | 36 | 15 | 29-82 | GTTGTGAATACCCTTCAAAATGAGAGCAGTCCCAAC |

**Additional file 3: Table S3. Cas9-related information for 39 Gram-negative type II system-containing bacteria**

| No. | Name of sequence | Bacteria | Length (aa) | Accession number | type |
| --- | --- | --- | --- | --- | --- |
| 1 | CRISPR-associated protein, Csx12 family | *Parasutterella excrementihominis* YIT 11859 | 1428 | EGG51390.1 | II-B |
| 2 | type II-B CRISPR-associated RNA-guided endonuclease Cas9/Csx12 | *Wolinella succinogenes* | 1409 | WP_011139431.1 | II-B |
| 3 | type II-B CRISPR-associated RNA-guided endonuclease Cas9/Csx12 | *Legionella pneumophila* | 1372 | WP_011212792.1 | II-B |
| 4 | type II-B CRISPR-associated RNA-guided endonuclease Cas9/Csx12 | *Sutterella wadsworthensis* | 1422 | WP_005430658.1 | II-B |
| 5 | type II-B CRISPR-associated RNA-guided endonuclease Cas9/Csx12 | *gamma proteobacterium* HTCC5015 | 1397 | WP_008284239.1 | II-B |
| 6 | HNH endonuclease family protein | *Francisella tularensis subsp. novicida* U112 | 1629 | AJI60231.1 | II-B |
| 7 | type II CRISPR RNA-guided endonuclease Cas9 | *Fusobacterium nucleatum* | 1374 | WP_005888649.1 | II-A |
| 8 | CRISPR-associated protein, SAG0894 family | *Treponema denticola* ATCC 35405 | 1395 | AAS10822.1 | II-A |
| 9 | CRISPR-associated protein, Csn1 family (plasmid) | *Ilyobacter polytropus* DSM 2926 | 1092 | ADO84368.1 | II-C |
| 10 | CRISPR-associated protein, Csn1 family | *Parabacteroides* sp. 20_3 | 1517 | EFK60619.1 | II-C |
| 11 | conserved hypothetical protein | *Bacteroides fragilis* NCTC 9343 | 1436 | CAH09630.1 | II-C |
| 12 | CRISPR-associated protein, Csn1 family | *Nitratifractor salsuginis* DSM 16511 | 1132 | ADV46720.1 | II-C |
| 13 | CRISPR-associated protein, Csn1 family | *Sphaerochaeta globosa* str. Buddy | 1179 | ADY14000.1 | II-C |
| 14 | type II CRISPR RNA-guided endonuclease Cas9 | *Methylosinus trichosporium* | 968 | WP_051418747.1 | II-C |
| 15 | hypothetical protein | *Prevotella* sp. C561 | 1424 | WP_009013303.1 | II-C |
| 16 | CRISPR-associated protein, Csn1 family | *Prevotella timonensis* CRIS 5C-B1 | 1218 | EFA98204.1 | II-C |
| 17 | CRISPR-associated protein, Csn1 family | *Prevotella timonensis* CRIS 5C-B1 | 1487 | EFA96738.1 | II-C |
| 18 | CRISPR-associated protein, Csn1 family | *Candidatus Puniceispirillum marinum* IMCC1322 | 1035 | ADE40787.1 | II-C |
| 19 | CRISPR-associated protein, Csn1 family | *Alicycliphilus denitrificans* K601 | 1029 | AEB82632.1 | II-C |
| 20 | conserved hypothetical protein | *Ralstonia syzygii* R24 | 1062 | CCA84553.1 | II-C |
| 21 | CRISPR-associated protein | *Dinoroseobacter shibae* DFL 12 = DSM 16493 | 1079 | ABV92149.1 | II-C |
| 22 | CRISPR-associated endonuclease Csn1 family protein | *Rhodospirillum rubrum* ATCC 11170 | 1173 | YP_425545.1 | II-C |
| 23 | CRISPR-associated protein, Csn1 family | *Azospirillum* sp. B510 | 1168 | BAI71538.1 | II-C |
| 24 | CRISPR-associated endonuclease, Csn1 family (plasmid) | *Nitrobacter hamburgensis* X14 | 1166 | ABE64718.1 | II-C |
| 25 | type II CRISPR RNA-guided endonuclease Cas9 | *Bradyrhizobium* sp. BTAi1 | 1052 | WP_049795381.1 | II-C |
| 26 | type II CRISPR RNA-guided endonuclease Cas9 | *Wolinella succinogenes* | 1059 | WP_011139289.1 | II-C |
| 27 | CRISPR-associated protein | *Campylobacter jejuni subsp. jejuni* NCTC 11168 = ATCC 700819 | 984 | YP_002344900.1 | II-C |
| 28 | Putative CRISPR associated protein | *Helicobacter mustelae* 12198 | 1024 | CBG39289.1 | II-C |
| 29 | CRISPR-associated protein, Csn1 family | *Acidovorax ebreus* TPSY | 1131 | ACM31549.1 | II-C |
| 30 | uncharacterized protein conserved in bacteria | *uncultured delta proteobacterium* HF0070_07E19 | 1011 | ADI19058.1 | II-C |
| 31 | CRISPR-associated protein, Csn1 family | *Parvibaculum lavamentivorans* DS-1 | 1037 | ABS61722.1 | II-C |
| 32 | hypothetical protein NMA0631 | *Neisseria meningitidis* Z2491 | 1082 | CAM07896.1 | II-C |
| 33 | unknown | *Pasteurella multocida subsp. multocida* str. Pm70 | 1056 | AAK03211.1 | II-C |
| 34 | CRISPR-associated protein cas9/csn1, subtype II/nmemi | *Bergeyella zoohelcum* ATCC 43767 | 1415 | EKB55676.1 | II-C |
| 35 | CRISPR-associated protein Cas9/Csn1, subtype II/NMEMI | *Porphyromonas* sp. oral taxon 279 str. F0450 | 1197 | EJU16049.1 | II-C |
| 36 | CRISPR-associated protein cas9/csn1, subtype II/nmemi | *Barnesiella intestinihominis* YIT 11860 | 1153 | EJZ61745.1 | II-C |
| 37 | CRISPR-associated protein cas9/csn1, subtype II/nmemi | *Odoribacter laneus* YIT 12061 | 1498 | EHP49880.1 | II-C |
| 38 | type II CRISPR RNA-guided endonuclease Cas9 | *Treponema* sp. JC4 | 1062 | WP_009105777.1 | II-C |
| 39 | type II CRISPR RNA-guided endonuclease Cas9 | *Rhodovulum* sp. PH10 | 1059 | WP_008386983.1 | II-C |

**Additional file 4: Fig. S1. Multiple sequence alignment of Cas2 amino acid sequences**

**

**

**Additional file 5: Fig. S2. Multiple sequence alignment of Cas1 amino acid sequences**

**

**

**Additional file 6: Fig. S3. Multiple sequence alignment of Cas9 amino acid sequences**

**
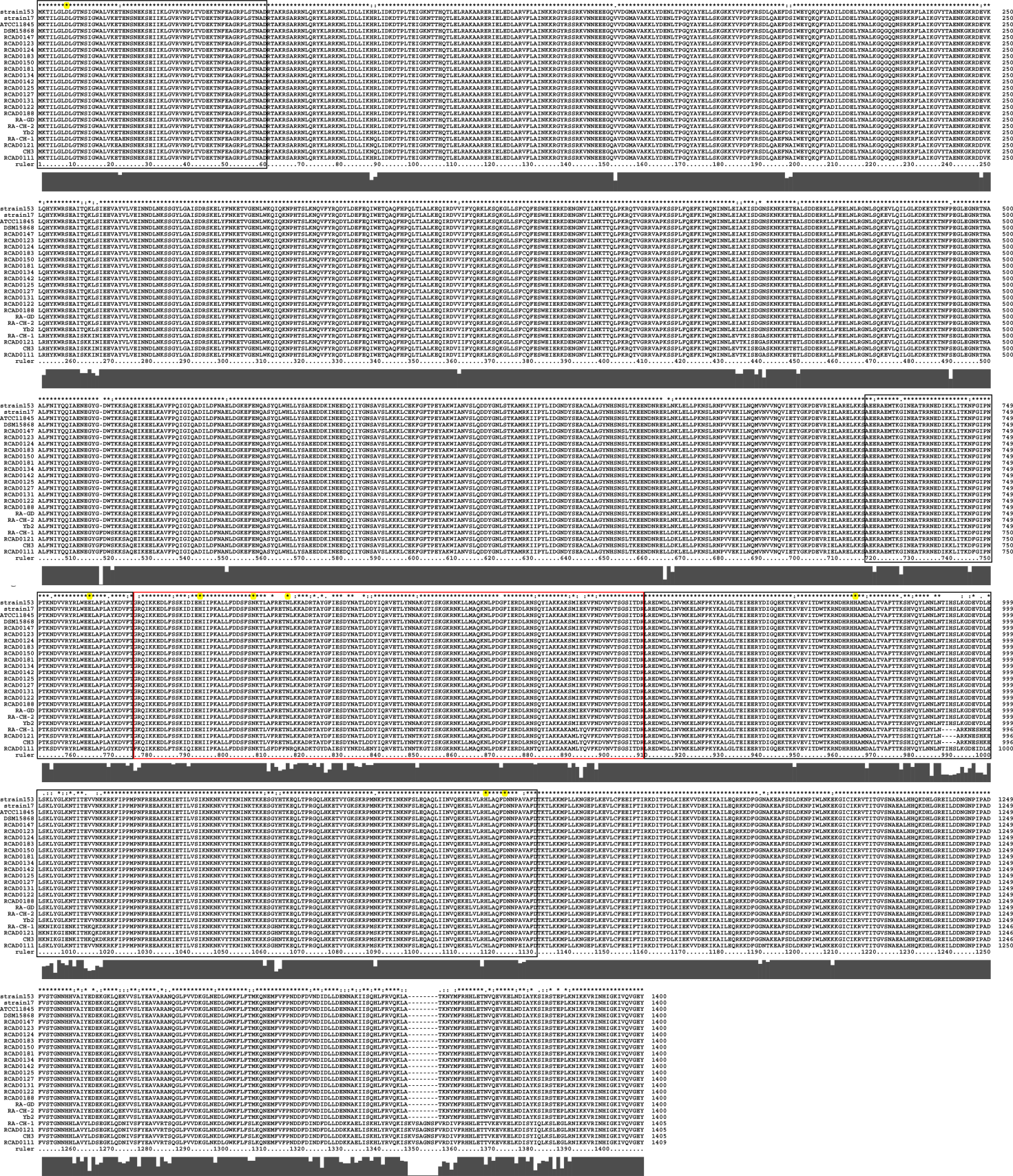
**

**Additional file 7: Fig. S4. Multiple sequence alignment for Cas9 from 40 Gram-negative type II system-containing bacteria**

>gi|329569623|gb|EGG51390.1|_CRISPR-associated_protein_Csx12_family_Parasutterella_excrementihominis_YIT_11859

M---------------------------------------------------------------GKTHIIGVGLDLGGTYTGTFITSHP----------SDEAEHRDHSSAFTVVN--------SEK-LSFSSKSRTAVRHRVRSYKGFDLRRRLLLLVAEYQLLQKKQTLAPE----------------------------------------------------ERENLRIALSGYLKRRGYARTEAET-DTSVLE------SLDPSVFSSAPSFTNFFNDSEPLNIQWEAIANSP------------------------------ETTKALNKELSGQKEADFKKYIKTSFPEYSAKEILANYVEG------------------------------------------------------------------------------RRAILDASKYIAN-LQSLGHKHRSKYLSDILQDMKRDSRITRLSEAFGSTDN-----LWRIIGNISNLQERAVRWYFNDAKFEQGQEQLDAVKLKNVLVRALKYLRSD-------------------DKEWSASQKQIIQSLEQSGDVLDVLAGLDPDRTIPP----------------YEDQNNRRPPEDQTLYLNPKALSSEYGEKWKSWANK-------------------------------------------FAGAYPLLTEDLTEILKN---TDRKSRIKIR-SDVLPDSDYRLAYILQRAFDRSIALDECSIRRTAEDFENG--VVIKNEKLEDVLSGHQLEEFLEFANRYYQETAKAKNG--------------------------------------------LWFP-ENALLERADLHPPMKNKILNVIVGQALGVS---PAEGTDFIEEIWNSKVKG--------------RSTVRSICNAIENERKTYGPYFSEDYKFVKTALKEGKTEKELSKKFAAVIKVLKMVSEVVPF-------IGKELRLSDEAQSKFDNLYSLAQLYNLIETERNGFSKVSLAAHLENAWRMTMTDG-------------SAQCCRLPADCVRPFDGFIRKAIDRN------SWEVAKRIAEEVKKSVDFTNGTVKIPVAIEANSFNFTASLTDLKYIQLKEQ------------------------------------KLKKKLEDIQRNEENQEKRWLSKEERIRADSHGICAYTGRPLDDVGEIDH---IIPRSLTLKKSESIYNSEVNLIFVSAQGNQEKKNNIY-----------LLSNLAKNYLAAVFGTSDLSQITNEIESTVLQLKAAG-------------------------------RLGYFDLLSEKERACARHALFLNSDSEA-----RRAVIDVLGSRRKASVNGTQAWFVRS---------IFSKVRQALAAWTQETGNELIFDAISVPAADSSEMRKRFAEYRPEFR-------------------------------------------------KPKVQPVASHSIDAMCI-----YLAACSDPFKTKRMGSQLAIYEPINFDN---------------------LFTGSCQVIQNTPRNFSDKTNIANSPIFKETIYAERFLDIIVS----------------------------------RGEIFIGYPSNMPFEEKPNRISIGGK----------DPFSILSVLGAYLDKAPSSEKEKLTIYRVVKNKAFELFSKVAGSKFTAEEDKAAKILEALHFVTVKQDVAATVSDLIKSKKELSKDSIENLAKQK-GCLKKVEYSS-KEFKFKGSLIIPAAVEWGKVLWNVFKENTAEELKDENALRKALEAAWPSSFGTRNLHSKAKRVFSLPVVATQSGAVRIR----------------RKTAFGDFVYQSQDTNN-LYSSFPVK----------NGKLDWSSPIIHPALQNRNLTAYGYRFVDHDRSISMSEFREVYNKDDLMR---------------IELAQGTSSRRYLRVEMPGEKFLAWFGE------------------NSISLGSSFKFSVSEVFDNKIYTENAEFTKFLPKPREDNKHN----------GTIFFELVG--PRVIFNYIVGGAASSLKEIFSEAGKERS--------------

>gi|499451967|ref|WP_011139431.1|_type_II-B_CRISPR-associated_RNA-guided_endonuclease_Cas9/Csx12_Wolinella_succinogenes

M------------------------------------------------------------------LVSPISVDLGGKNTGFFSFT------------DSLDNSQSGTVIYDES-------------FVLSQVGRRSKRHSKRNNLRNKLVKRLFLLILQEHHGLSIDVLP------------------------------------------------------------DEIRGLFNKRGYTYAGFELDEKKKDA------LESDTLKEFLSEKLQSIDRDSDVEDFLN-------------------------------------QIASNAESFKDYKKG-------------------------------------------------------------------------------------------------------FEAVFASATHSPNKKLELKDELKSEYGENAKELLAGLRVTKEILDEFD--------------KQENQGNLPRAKYFEELGEYIATNEKVKSFFDSNSLKLTDMTKLIGN----------------------ISNYQLKELRRYFNDKEMEKGDIWIPNKLHKITERFVRSWHPKNDADRQRRAELMKDLKSKEIMELLTTTEPVMTIPPYDDMNNRG-----------------------------------------AVKCQTLRLNEEYLDKHLPNWRDIAKRLNHGKFNDDLADSTVKGYSEDSTLLHRLLDTSKEIDIYELRGKKP------NELLVKTLGQSDANRLYGFAQNYYELIRQKVRAGIWV--------------------------------PVKNKDDSLNLEDNSNMLKRCNHNPPHKKNQIHNLVAGILGVK--LDEAKFAEFEKELWSAKVG--------------NKKLSAYCKNIEELRKTHGNTFKID---IEELRKKDPAELSKEEKAKLRLTDDVILNEWSQK-------IANFFDIDDKHRQRFNNLFSMAQLHTVIDTPRSGFSSTCKRCTAENRFRSETAFYNDETG--EFHKKATATCQRLPADTQRPFSGKIERYIDKL------GYELAKIKAKELEGMEAKEIKVPIILEQNAFEYEESLRKSKTGSNDRVINS------------------------------------KKDRDGKKLAKAKENAEDRLKDKDKRIKAFSSGICPYCGDTIGDDGEIDH---ILPRSHTLKIYGTVFNPEGNLIYVHQKCNQAKADS-I-----------YKLSDIKAGVSAQWIEEQVANIKGYKTFSVLSAEQQK-------------------------------AFRYALFLQNDNEAYKKVVDWLRTDQSARVNGTQKYLAKKIQEKLTKMLPNKHLS---------------FEFILADATEVSELRRQYARQNPLLAKAEKQAPSSHAIDAVMAFVARYQKVFKDG--------------------------------TPPNADEVAKLAMLDSWNPASNEP-----LTKGLSTNQKIEKMIKSGDYGQKNMREVFGKSIFGENAIG--ERYKPIVVQEGGYYIGYPATVKKGYELKNCKVVTSKNDIAKLEKIIK--------------------------------------NQDLISLKENQYIKIFSINKQTISELSN-------RYFNMNYKNLVERDKEIVGLLEFIVENCRYYTKKVDVKFAPKYIHETKYPFYDDWRRFDEAWRYLQ--------------------------------------ENQNKTSSKDRFVIDKSSLNEYYQPDKNEYKLDVDTQPIWDDFCRWYFLDRYKTANDKKSIRIKARKTFSLLAESGVQGKVFR-------------AKRKIPTGYAYQALPMDNNVIAGDYANILLEAN------------------SKTLSLVPKSGISIEKQLDKKLDVIKKTDVRGLAIDN---------------NSFFNADFDTHGIRLIVENTSVKVGNFPISAIDKS----------AKRMIFRALFEKEKGKRKKKTTISFKESGPVQDYLKVFLK----------------KIVKIQLRTDGSISNIVVRKNAADFTLSFRSEHIQKLLK-----------

>gi|499526152|ref|WP_011212792.1|_type_II-B_CRISPR-associated_RNA-guided_endonuclease_Cas9/Csx12_Legionella_pneumophila

M--------------------------------------------------------------ESSQILSPIGIDLGGKFTGVCLSH-------------LEAFAELPNHANTKYS----VILIDHNNFQLSQAQRRATRHRVRNKKRNQFVKRVALQLFQHILSRDLNAK-----------------------------------------------------------EETALCHYLNNRGYTYVDTDLDEYIKDE------TTINLLKELLPSESEHNFIDWFLQKMQSSEFR-------------------------------KILVSKVEEKKDDKELKN------------------------------------------------------------------------------------------------------AVKNIKNFITGFEKNSVEGHRHRKVYFENIKSDITKDNQLDSIKKKIPS-----------VCLSNLLGHLSNLQWKNLHRYLAKNPKQFDEQTFGNEFLRMLKNFR----------------------HLKGSQESLAVRNLIQQLEQSQDYISILEKTPPEITIPP------------YEARTNTGMEKDQSLLLNPEKLNNLYPNWRNLIPG--------------------------------------------IIDAHPFLEKDLEHTKLR-----DRKRIISPSKQDEKRDSYILQRYLDLNKKIDKFKIKKQLSFLGQGKQLPANLIETQKEMETHFNSSLVSVLIQIASAYNKEREDAAQG--------------------------------------------IWFDNAFSLCELSNINPPRKQKILPLLVGAILSEDFINNKDKWAKFKIFWNTHKIG--------------RTSLKSKCKEIEEARKNSGNAFK--------IDYEEALNHPEHSNNKALIKIIQTIPDIIQA-------IQSHLGHNDSQALIYHNPFSLSQLYTILETKRDGFHKNCVAVTCENYWRSQKTEIDPEIS----------YASRLPADSVRPFDGVLARMMQRL------AYEIAMAKWEQIKHIPDNSSLLIPIYLEQNRFEFEESFKKIKGSSSD----------------------------------------------KTLEQAIEKQNIQWEEKFQRIINASMNICPYKGASIGGQGEIDH---IYPRSLSKKHFGVIFNSEVNLIYCSSQGNREKKEEHY-----------LLEHLSPLYLKHQFGTDNVSDIKNFISQNVANIKK----------------------------------YISFHLLTPEQQKAARHALFLDYDDEA-----FKTITKFLMSQQKARVNGTQKFLG-------------KQIMEFLSTLADSKQLQLEFSIKQITAEEVHDHRELLSKQEP----------------------------------------------------KLVKSRQQSFPSHAIDA-----TLTMSIGLKEFPQFSQELDNSWFIN-----------------------HLMPDEVHLNPVRSKEKYNKPNISSTPLFKDSLYAERFIPVWVKG--------------------------ETFAIGFSEKDLFEIKPSNKEKLFTLLKTYSTK-----------------NPGESLQELQAKSKAKWLYFPINKTLALEFLHHYFHKEIVTPDDTTVCHFIN--------------SLRYYTKKESITVKILKEPMPV-LSVKFESSKKNVLGSFKHTIALPATKDWERLFNHPNFLALKANPAPNPKEFNEFIRKYFLSDNNPNSDIPNNGHNIKPQKHKAVRKVFSLP---------------VIPGNAGTMMRIRRKDNKGQPLYQLQT---------IDDTPSMGIQINEDRLVKQEVLMDAYKTRNLSTIDGINNSEGQAYATFDN--------------WLTLPVSTFKPEIIKLEMKPHSKTRRYIRITQSLAD----------FIKTIDEALMIKPSDSIDDPLNMPNEIVCKNKLFGNELKPR-------------DGKMKIVSTGKIVTYEFESDSTPQWIQTLYVTQLKKQP--------------

>gi|491573077|ref|WP_005430658.1|_type_II-B_CRISPR-associated_RNA-guided_endonuclease_Cas9/Csx12_Sutterella_wadsworthensis

M------------------------------------------------------------TQSERRFSCSIGIDMGAKYTGVFYALFD----------REELPTNLNSKAMTLVM--------PETGPRYVQAQRTAVRHRLRGQKRYTLARKLAFLVVDDMIKKQEKRLTDE----------------------------------------------------EWKRGREALSGLLKRRGYSRPNADGEDLTPLE------NVRADVFAAHPAFSTYFSEVRSLAEQWEEFTAN---------------------------------ISNVEKFLGDPNIPADKEFIEFAVAEGLIDKTEKKAYQS------------------------------------------------------------------------------ALSTLRANANVLTGLRQMGHKPRSEYFKAIEADLKKDSRLAKINEAFGGAER-----LARLLGNLSNLQLRAERWYFNAPDIMKDR-GWEPDRFKKTLVRAFKFFHP--------------------AKDQNKQHLELIKQIENSEDIIETLCTLDPNRTIPP----------------YEDQNNRRPPLDQTLLLSPEKLTRQYGEIWKTWSAR-------------------------------------------LTSAEPTLAPAAEILERS---TDRKSRVAVNGHEPLPTLAYQLSYALQRAFDRSKALDPYALRALAAGSKSNK-LTSARTALENCIGGQNVKTFLDCARRYYREADDAKVG--------------------------------------------LWFDNADGLLERSDLHPPMKKKILPLLVANILQTD---ETTGQKFLDEIWRKQIKG--------------RETVASRCARIETVRKSFGGGFNIAYN---TAQYREVNKLPRNAQDKELLTIRDRVAETADF-------IAANLGLSDEQKRKFANPFSLAQFYTLIETEVSGFSATTLAVHLENAWRMTIKDAVINGE-----TVRAAQCSRLPAETARPFDGLVRRLVDRQ------AWEIAKRVSTDIQSKVDFSNGIVDVSIFVEENKFEFSASVADLK-------------------------------------------KNKRVKDKMLSEAEKLETRWLIKNERIKKASRGTCPYTGDRLAEGGEIDH---ILPRSLIKDARGIVFNAEPNLIYASSRGNQLKKNQRY-----------SLSDLKANYRNEIFKTSNIAAITAEIEDVVTKLQQTH-------------------------------RLKFFDLLNEHEQDCVRHALFLDDGSEA-----RDAVLELLATQRRTRVNGTQIWMIKN---------LANKIREELQNWCKTTNNRLHFQAAATNVSDAKNLRLKLAQNQPDFE-------------------------------------------------KPDIQPIASHSIDALCS-----FAVGSADAERDQ---NGFDYLDGKTVLG---------------------LYPQSCEVIHLQAKPQEEKSHFDSVAIFKEGIYAEQFLPIFTL----------------------------------NEKIWIGYETLNAKGERCGAIEVSGK----------QPKELLEMLAPFFNKPVG-DLSAHATYRILKKPAYEFLAKAALQPLSAEEKRLAALLDALRYCTSRKSLMS----LFMAANGKSLKKREDVLKPK-LFQLKVELKGEKSFKLNGSLTLPVKQDWLRICDSPELADAFGKPCSADELTSKLARIWKRPVMRDLAHAPVRREFSLPAIDNPSGGFRIR----------------RTNLFGNELYQVHAINAKKYRGFASA----------GSNVDWSKGILFNELQHENLTECGGRFITSADVTPMSEWRKVVAEDNLS----------------IWIAPGTEGRRYVRVETTFIQASHWFEQSVENWAI----------TSPLSLPASFKVDKPAEFQKAVG---TELSELLGQPRSE----------------IFIENVGNAKHIRFWYIVVSSNKKMNESYNNVSKS----------------

>gi|495559660|ref|WP_008284239.1|_type_II-B_CRISPR-associated_RNA-guided_endonuclease_Cas9/Csx12_gamma_proteobacterium_HTCC5015

M---------------------------------------------------------------TKNYISPIAIDLGAKFTGVALYQY---------LEGADCTQEVAKGLLVDDR----------GNVTWSQEGRRGKRHQVRGYKRRKMAKRLLWLILDSEYGIKREEVTEP--------------------------------------------------------LLKFINGLLNRRGYTYISEEVDEESMNV------SPLPFSEMMPDYFNSSAPLLEQLAKLLS-------------------------------------DKNKLVRFRAEGKIPS---------------------------------------------------------------------------------------------------------NKNEFKKLLDTALDGKYKDEKKELSEAWGNILIASEN--------------------VLKSTVDGHKSRSEYLANIKEDIKSNEELEKQISSKEIDGFYNLVG------------------------HLSNFQLRLLRKYFNDPNMSGVSYWDEKRLEKYFYQWVQGWHTKGGTDEAEKKNIILKTKGAPLLKTLKSLSADLTIPPYEDQNNRR-----------------------------------------PPKCQSVLLSDEKLTMHYPKWKEWVGQLVKQNDNAYLNENVTLANALHRIVERSRSIDPYQLRLLISITDAEKRNDLAGYKRLKLSLGSEVDEFLLLVKNIVDETKEAREG--------------------------------------------LWFETENKLFFKCGKTPPRKEKLKSTLLSAVLGKN--LSDDEQSSFIEEFWKSGTP--------------KIERRNVRGWCRLASQVQKTYGVYLKEYGLQQLHKLEAGKKLDDKPLALLYKNSGLIASKIG---------EALNIEPDEVSRFASPHSLAQIFNIIEGDVAGFNKTCRACTYENIWRMQEEKVESLLTNQLLSEIHGERKVPLKSAMCTRLSADSTRPFDGQ------MASIIEHIARKIAQHKIAQINDVPKEFSIDIPIIIESNQFSFTAELEEIKR-----------------------------------GRGSAKAKKAKELGEKSKAGWVSKTERIKTSSEGICPYTGAPLGGSGEIDH---IIPRSLTGRTKKTVFNSEANLIYCSSKGNHDKGNRVY-----------VIEQLNDKYLKKQFSTSDVNLIKKKIKTTIQRFTEGG-------------------------------EKLRSFSELSREDQKAFRHALFVPELKS-----EVTSLLAVKNITRVNGTQAWLAK---------------KIASLLAEHLDKQGRDYTLSAHQIDPWSVSKQRKMLASAEPIWAK-------------------------------------------------KDPQPAASHVVDAVCT-----FLEALEQPHTASRLKTISSTSFEKTGWRS-------------------ALIPDLIKVDALDRRPKYRRYNIGSTSLFKDGIYAERFLPILID----------------------------------ENGLMAGYDIDNSLKAKGADVVFESLS------------PFLLFKGEEVGAQSLSDWQERIDGRYLYMSIDKVKAFDYLQEKVGEKDIAAELLNSIHFTQRKTELRAKFS--DDSGKKMKTLDAIRKSLKL-TVTVNEIGKRKEKCGFSGTIGIPAKSAWENLLDEPLLETYWGTKMPPQEIWEKVYRKHFPRNIPNQAHRKVRKDFSLPVVDSVSGGFRVK----------------RKTPNGYNYQLLAIDGYSAVGFKKEG--------------------DNVDFKSPALVPQIAESKSVTPISSELVHLDKNEIVYFDEWR--------------KIDISDSDLKQFVSSLELAPGSQNRFYIRFTVDE----------DQFERHFKSALRVNGIQDLDTVNKTFDWNREIPSLLIPPR---------------SNLFLLETGQKITFEYIANGANAEVKKAYSLRRA-----------------

>gi|754269625|gb|AJI60231.1|_HNH_endonuclease_family_protein_Francisella_tularensis_subsp._novicida_U112

M----------------------------------------------------------------NFKILPIAIDLGVKNTGVFSAFY-------QKGTSLERLDNKNGKVYELSK----------DSYTLLMNNRTARRHQRRGIDRKQLVKRLFKLIWTEQLNLEWDKDTQQAISFLFNRRGFSFITDG--------------------YSPEYLNIVPEQVKAILMDIFDDYNGEDDLDSYLKLATEQESKISEIYN----KLMQKILEFKLMKLCTDIKDDKVSTKTLKEITSYEFELLADYLAN------YSESLKTQKFSYTDKQGNLKELSYYHHDKYNIQEFLKRHATINDRILDTLLTDDLDIWNFN-----------------------------------------------FEKFDFDKNEEKLQNQEDKDHIQAHLHHFVFAVNKIKSEMASGGRHRSQYFQEITNVLDENNHQEGYLKNFCEN-----------LHNKKYSNLSVKNLVNLIGNLSNLELKPLRKYFNDKIHAKADHWDEQKFTETYCHWILGEWRVGVKDQDKKDGAKYSYKDLCNELKQKVTKAGLVDFLLELDPCRTIP---------PYLDNNNRKPPKCQSLILNPKFLDNQYPNWQQYLQELKKLQS--------------------------------------IQNYLDSFETDLKVLKSSKDQPYFVEYKSSNQQIASGQRDYKDLDARILQFIFDRVKASDELLLNEIYFQAKKLKQKASSELEKLESSKKLDEVIANSQLSQILKSQHTNGIFEQGTFLHLVCKYYKQRQRARDSRLYIMPEYRYDKKLHKYNNTGRFDDDNQLLTYCNHKPRQKRYQLLNDLAGVLQVSPNFLKDKIGSDDDLFISKWLVEHIRGFKKACEDSLKIQKDNRGLLNHKINIARNTKGKCEKEIFNLICKIEGSEDKKGNYKHGLAYELGVLLFGEPN--------EASKPEFDRKIKKFNSIYSFAQIQQIAFAERKGNANTCAVCSADNAHRMQQIKITEPVEDNKDKIILSAKAQRLPAIPTRIVDGAVKKMATILAKN--IVDDNWQNIKQVLSAKHQLHIPIITESNAFEFEPALADVKGKSLKDRR-----------------------------------------------KKALERISPENIFKDKNNRIKEFAKGISAYSGANLTDGDFDGAKEELDHIIPRSHKKYGTLNDEANLICVTRGDNKNKGNR-----------IFCLRDLADNYKLKQFETTDDLEIEKKIADTIWDANKKD--------------------------------FKFGNYRSFINLTPQEQKAFRHALFLADENPIKQAVIRAINNRNRTFVNGTQRYFAEVLAN-----NIYLRAKKENLNTDKISFDYFGIPTIGNGRGIAEIRQLYEKVDSDIQAYAK----------------------------------------------GDKPQASYSHLIDAMLA-----FCIAADEHRNDGSIGLEIDKNYSLYPLDKNTGEVFTKDIFS------QIKITDNEFSDKKLVRKKAIEGFNTHRQMTRDGIYAEN-----------------------------------------YLPILIHKELNEVRKGYTWKNSEEIKIFKGKKYDIQQLNNLVYCLKFVDKPISIDIQISTLEELRNILTTNNIAATAEYYYINLKTQKLHEYYIENYNTALG------------------------------------YKKYSKEMEFLRSLAYRSERVKIKSIDDVKQVLDKDSNFIIGKITLPFKKEWQRLYREWQNTTIKDDYEFLKSFFNVKSITKLHKKVR-----KDFSLPISTNEGKFLVKRKTWDNNFIYQILNDSDSR-------------------ADGTKPFIPAFDISKNEIVEAIIDSFTSKNIFWLPKNIELQKVD---------NKNIFAIDTSKWFEVETPSDLRDIGIATIQYKIDN----------NSRPKVRVKLDYVIDDDSKINYFMNHSLLKSRYPDKVLEILK---------------QSTIIEFESSGFNKTIKEMLGMKLAGIYNETSNN---------------

>gi|492568239|ref|WP_005888649.1|_type_II_CRISPR_RNA-guided_endonuclease_Cas9_Fusobacterium_nucleatum

M-------------------------------------------------------------KKQKFSDYYLGFDIGTNSVGWCVTD-----------LDYNVLRFNKKDMWGSR----------LFDEAKTAAERRVQRNSRRRLKRRKWRLNLLEEIFSDEIMKIDSNFFRRLKESSLWLEDKNSKEKFTLFNDDNYKDYDFYKQYPTIFHLRDELIKNPEKKDIRLIYLALHSIFKSRGHFLFEGQNLKEIKNFE------TLYNNLISFLEDNGINKSIDKDNIEKLEKIICDSGKGLKDKE---KEFKGIFNSDKQLVAIFKLSVGSSVSLNDLFDTDEYKKEEVEKEKISFREQIYEDDKPIYYSILG-------------------------------------------------------------------EKIELLDIAKSFYDFMVLNNILSDSNYISEAKVKLYEEHKKDLKNLKYIIRKYNKENYDKLF----KDKNENNYPAYIGLNKEKDKKEVVEKSRLKIDDLIKVIKGYLPKPERIE---------------EKDKTIFNEILNKIELKTILPKQRISDNGTLPYQIHEVELEKILENQSKYYDFLN-YEENGVSTKDKLLKTFKFRIPYYVGPLNSYHKDKGGN-SWIVR-----------------------------KEE---GKILPWNFEQKVDIEKSA---EEFIKRMTNKCTYLNGEDVIPKDSFLYSEYIILNELNKVQVN---DEFLNE--ENKRKIIDELFKENKKVSEKKFKEYLLVNQIANRT--VELK--------------------------------GIKD---SFNSNYVSYIKFKDIFGEKLNLDIYKEISEKSILWKCLY--GDDKKIFEKKIKNEYG--------------DILNKDEIKKINSFKFNTWGR----------------LSEKLLTGIEFINLETGECYSS------------VMEALRRTNYNLMELLSSKFTLQESIDNENK----EMNEVSYRDLIEESYVSP----------------SLKRAILQTLKIYEEIKKITGRV------PKKVFIEMARGGDESMKNKKIPARQEQLKKLYDSCGNDIANFSIDIKEMKN------------------------------------------SLSSYDNNSLRQKKLYLYYLQFG----KCMYTGREIDLDRLLQNNDTYDIDHIYPRSKVIKDDSFDNLVLVLKNENAEKSNE-------------YPVKKEIQEKM-----KSFWRFLKEKNFISDEKYK--------------------------------------RLTGKD---DFELRGFMARQLVN-----VRQTTKEVGKILQQIEPEI-------------------KIVYSKAEIASSFREMFDFIKVR----------------------------------------------------------------------------ELNDTHHAKDA-----YLNIVAGNVYNTKFTEKPYRYLQEIKEN------YDV-------KKIYNYDIKN----AWDKE--------NSLEIVKKNMEKN--------------------------------------------TVNITRFIKEE-------------------------------KGELFNLNPIKKGETSNEIISIKPKLYDGKDNKLNEKYGYYTSLK----------------------------------------------------AAYFIYVEHEKKNKKVKTFERITRIDSTLIKNEKNLIK-YLVSQK-KLLNPKIIKKIYKEQTLIIDSYPYTFTGVDSNKKV----------------ELKNKKQLYLEKKYEQILKNALKFVE----------DNQ-------------GETEENYKFIYLK--KRNNNEKNETIDAVKER------------------YNIEFN---EMYDKFLEKLS----SKDYKNYINN----------KLYTNFLNSKEKFKKLKLWEKSLILREFLKIFNKNTYGKY---------EIKDSQTKEKLFSFPEDTGRIRLGQSSLGNNKELLEESVTGLFVKKIKL--------

>gi|41815893|gb|AAS10822.1|_CRISPR-associated_protein_SAG0894_family_Treponema_denticola_ATCC_35405

M--------------------------------------------------------------KKEIKDYFLGLDVGTGSVGWAVTD-----------TDYKLLKANRKDLWGMR----------CFETAETAEVRRLHRGARRRIERRKKRIKLLQELFSQEIAKTDEGFFQRMKESPFYAEDKTILQENTLFNDKDFADKTYHKAYPTINHLIKAWIENKVKPDPRLLYLACHNIIKKRGHFLFEG-DFDSENQFD------TSIQALFEYLRED-MEVDIDAD-SQKVKEILKDSSLKNSEKQSRLNKILGLKPSDKQKKAITNLISGNKINFADLYDNPDLK--DAEKNSISFSKDDFDALSDDLASILG-------------------------------------------------------------------DSFELLLKAKAVYNCSVLSKVIGDEQYLSFAKVKIYEKHKTDLTKLKNVIKKHFPKDYKKVFGYNKNEKNNNNYSGYVGVCKTKSKKLIINNS-VNQEDFYKFLKTILSAKSEIK---------------EVN-----DILTEIETGTFLPKQISKSNAEIPYQLRKMELEKILSNAEKHFSFLKQKDEKGLSHSEKIIMLLTFKIPYYIGPINDNHKKFFPDRCWVVK-----------------------------KEKSPSGKTTPWNFFDHIDKEKTA---EAFITSRTNFCTYLVGESVLPKSSLLYSEYTVLNEINNLQIIIDGKNICDI--KLKQKIYEDLFKKYKKITQKQISTFIKHEGICNKTDEVIIL--------------------------------GIDK---ECTSSLKSYIELKNIFGKQVDEISTKNMLEEIIRWATIYDEGEGKTILKTKIKAEYG--------------KYCSDEQIKKILNLKFSGWGR----------------LSRKFLETVTSEMPGFSEPVN-------------IITAMRETQNNLMELLSSEFTFTENIKKINSGFEDAEKQFSYDGLVKPLFLSP----------------SVKKMLWQTLKLVKEISHITQAP------PKKIFIEMAKG--AELEPARTKTRLKILQDLYNNCKNDADAFSSEIKDLSG------------------------------------------KIENEDNLRLRSDKLYLYYTQLG----KCMYCGKPIEIGHVFDTSN-YDIDHIYPQSK-IKDDSISNRVLVCSSCNKNKEDK-------------YPLKSEIQSKQ-----RGFWNFLQRNNFISLEKLN--------------------------------------RLTRATPISDDETAKFIARQLVE-----TRQATKVAAKVLEKMFPET-------------------KIVYSKAETVSMFRNKFDIVKCR----------------------------------------------------------------------------EINDFHHAHDA-----YLNIVVGNVYNTKFTNNPWNFIKEKRDNPKIADTYNY-------YKVFDYDVKRNNITAWEKG--------KTIITVKDMLKRN--------------------------------------------TPIYTRQAACK-------------------------------KGELFNQTIMKKG------LGQHPLKKEGPFSNIS-KYGGYNKVS----------------------------------------------------AAYYTLIEYEEKGNKIRSLETIPLYLVKDIQKDQDVLKSYLTDLLGKKEFKILVPKIKINSLLKINGFPCHITGK-TNDSF----------------LLRPAVQFCCSNNEVLYFKKIIRFSE----------IRSQ--------REKIGKTISPYEDLSFRSYIKENLWKKTKNDEIGEKEF----------------YDLLQKKNLEIYDMLLTKHK----DTIYKKRPNS----------ATIDILVKGKEKFKSLIIENQFEVILEILKLFSATRN-------------VSDLQHIGG--SKYSGVAKIGNKISSLDN-CILIYQSITGIFEKRIDLLKV-----

>gi|309749707|gb|ADO84368.1|_CRISPR-associated_protein_Csn1_family_(plasmid)_Ilyobacter_polytropus_DSM_2926

M-------------------------------------------------------------------KYSIGLDIGIASVGWSVIN---KDKER--------IEDMGVRIFQKAE--------NPKDGSSLASSRREKRGSRRRNRRKKHRLDRIKNILCESGLVK-----------------------------------------------------------KNEIEKIYKN-AYLKSPWELRAKSLEAKISNK------EIAQILLHIAKRRGFKSFRKTDRNADD---------------------------------------TGKLLSGIQENKKI----------------------------------------------------------------------------------------------------------MEEKGYLTIGDMVAKD-PKFNT-------HVRNKAG-------------------------SYLFSFSRKLLEDEVRKIQAKQKELGNTHFTDDVLEKYIEVF--N---------------SQRNFDEGPSKPSPYYSEIGQIAKMIGNCTFESSEK--------------------------RTAKNTWSGERFVFLQKLNNFRIVGLS------------------------------------------------GKRPLTEEERD-------IVEKEVYLKKE--------VRYEKLR----KILYLKEEERFGDLNYSK---------DEKQDKKTEKTKFISLIGNYTIKKLNLSEKLKS---E--------------------------------IEE--------DKSKLDKIIEILTFNKSDKTIESNLKKLELS----REDI--EILLSEEFSGTL--------------NLSLKAIKKILPYLE----------------------KGLSYNEACEKAD------------------------YDYKNN--GIKFKRGELLPVVD---------------------KDLIANP----------------VVLRAISQTRKVVNAIIRKYGT-------PHTIHVEVARDLAKSYDDRQTIIKENKKRELENEKTK---KFISE-------------------------------------------------EFGIKNVKGKLLLKYRLYQEQE---GRCAYSRKELSLSEVILDESMTDIDHIIPYSR-SMDDSYSNKVLVLSGENRKKSNL-------------LPKEYFD------RQGRDWDTFVLNVKAMKIHPR-------------------------------------KKSNLLKEKFTREDNKDWKSRALND-----TRYISRFVANYLENALEYRDDSP-------------KKRVFMIPGQLTAQLRARWRLN----------------------------------------------------------------------------KVRENGDLHHALDA-----AVVAVTDQKAINNISNISRYKELKNCKDVIPSIEY-------------HADEETGEVYFEEVKD---TRFPMPWSGFDLELQKRLESE----------NPREEFYNLLSDKRYLGWFNYEEGFIEKLRPVFVSRMPNRG------------------------------VKGQAHQETIRSSKK--ISNQIAVSKKPLNSIKLKDLEKMQGRD-------------------------------------------------------------------TDRKLYEALKNRLEEYDDKPEKAFAEP--FYKPTNSGKRG-PLVRGIKVEEKQNVGVYVNG--GQASN----------------GSMVRIDVFR--KNGKFYTVPIYVHQ------------------------TLLKELPNRAING-KP---YKDWDLIDGSFE----------------------FLYSFYPNDLIEIEFGKSKSIKNDNKLTKTEIP----------EVNLSEVLGYYRGMDTSTGAATIDTQDGKIQMR---------------------------IGIKTVKNIKKYQVDVLGN-VYKVKREKRQTF--------------

>gi|300829971|gb|EFK60619.1|_CRISPR-associated_protein_Csn1_family_Parabacteroides_sp._20_3

M-------------------------------------------------------------------KKIVGLDLGTNSIGWALIN---AYINK---EHLYGIEACGSRIIPMD--AAILGNFDKGNSISQTADRTSYRGIRRLRERHLLRRERLHRILDLLGFLPKHYSDSL--------NRYGKFLNDIECKLPWV--KDETGSYKFIFQESFKEMLANFTEHHPILIANNKKVPYDWTIYYLRKKALTQKISKE------ELAWILLNFNQKRGYYQLRGEEEE-TPNKLVEYYSLKVEKVEDSGERKGKDTWYNVHLENGMIYRRTSNIPL-DWEGKTKEFIVTTDLEADG-SPKKDKEGNIKR------------------------------------------------------------------------SFRAPKDDDWTLIKKKTEADIDKIKMTVGAYIYDTLLQKPDQKIRGKLVRTIERKYYKNELYQILKTQSEFHEELRDKQLYIACLNELYPNNEPRRNSISTRDFCHLFIE------------------------------DIIFYQRPLKSKKSLIDNCPYEENRYIDKESGE------------IKHASIKCIAKSHPLYQEFRLWQFIVNLRIYRKET------------------------------------------DVDVTQELLPTEADYV--------TLFEWLNEK-------KEIDQ----KAFFKYPPFGFKKTTSN-YRWNY-------VED--KPYPCNETHAQIIARLGKAHIPKAFLS-K-----------------------------------------------EK--EETLWHILYSIEDKQEIEKALHSFANKNNLSEEFIEQFKNFPPFKKEYG--------------SYSAKAIKKLLPLMRMGKYWS---------IENIDNGTRIRINKIIDGEYDENIRERVRQKA-------INLTDITHFRALPLWLACYLVYDRHSEVKDIVKWKTPKDIDLYLKSFKQHSLRNP----------------IVEQVITETLRTVRDIWQQVGH-------IDEIHIELGREMKNPADKRARMSQQMIKNENTNLRIK---ALLTEFLNPEFGIENVRPYSPSQQDLLRIYEEGVLNSILELPEDIGIILGKFNQTDTLKRPTRSEILRYKLWLEQK---YRSPYTGEMIPLSKLFTP--AYEIEHIIPQSR-YFDDSLSNKVICESEINKLKDRS-------------LGYEFIKNHHGEKVEL-AFDKPVEVLSVEAYEKLVHE-------------------------------SYSHNRSKMKKLLMEDIPDQFIERQLND-----SRYISKVVKSLLSNIVREENEQEA-----------ISKNVIPCTGGITDRLKKDWGINDVWNKIVLPRFIRLNELTESTRFTSINTNN---TMIPSM-----------------------------PLELQKGFNKKRIDHRHHAMDA-----IIIACANRNIVNYLNNVSASKNTKITRRD--LQTLLC-------HKDKTDNNGNYKWVIDKP---------WET--FTQDTLTA------------------------------------------LQKITVSFKQNLRVINKTTNHYQHYE-NGKKIVSNQSKGDSWAIRKSMHKETVHG--EVNLRMIKTVSFNEALKKPQAIVEMDLKKKILAMLELGYDTKRIKNYFEENKDTWQDINPSKIKVYYFTKETKD-RYFAVRKPIDTSFDKKKIKESITDTGIQQIMLRHLETKDNDPTLAFSPDGIDEMNRNILILNKGKKHQPIYKVRVYEKA-EKFTVG--------------------QKGNKRTKFVEAAKGTNLFFAIYE------------TEEIDKDTKKVIRKRSYSTIPLNVVIERQKQGLSSAPE-DENGNL----------------------PKYILSPNDLVYVPTQEEI---NKGEVVMPIDR----------DRIYKMVDSSGITANFIPASTANLIFALPKATAEIYCNGENCIQNEYGIGSPQSKNQKAITGEMVKEICFPIKVDRLGNIIQVGSCILTN----------------

>gi|60494823|emb|CAH09630.1|_conserved_hypothetical_protein_Bacteroides_fragilis_NCTC_9343

M-------------------------------------------------------------------KRILGLDLGTNSIGWALVN---EAENK---DERSSIVKLGVRVNPLT--VDELTNFEKGKSITTNADRTLKRGMRRNLQRYKLRRETLTEVLKEHKLITEDT------------------------------------------------------------ILSENGNRTTFETYRLRAKAVTEEISLE------EFARVLLMINKKRGYKSSRKAKGVEEGTLIDGMDIARELYNNNLTPGELCLQLLDAGKKFLPDFYRSDLQNELDRIWEKQKEYYPEILTDVLKEELRGKKRDAVWAICAKYFVWKENYTEWNKEKGKTEQQEREHKLEGIYSKRKRDEAKRENLQWRVNGLKEKLSLEQLVIVFQEMNTQINNSSGYLGAISDRSKELYFNKQTVGQYQMEMLDKN---------------------------PNASLRNMVFYRQDYLDEFNMLWEKQAVYHK-ELTEELKKEIRD------------------------------IIIFYQRRLKSQKGLIGFCEFESRQIEVDIDGK----------KKIKTVGNRVISRSSPLFQEFKIWQILNNIEVTVVGKKRKRRKLKENYSALF--------------------EELNDAEQLELNGSRRLCQEEKE--------LLAQELFIR-------DKMTKSEVLKLLFDNP---QELDLNF-----------------KT-IDGNKTGYALFQAYSKMIEMSGHEPVD-----------------------------------------------FKKPVEKVVEYIKAVFDLLNWNTDILGFNS-----NE---ELDNQPYYKLWHL--------------LYSFEGDNTPTGNGR---LIQ---------KMTELYGFEKEYATILANVSFQDDYGSLSAKA-------IHKILPHLKEGNRYDVACVYAGYRHSESSLTREEIANKVLKDRLMLLPKNSLHNP----------------VVEKILNQMVNVINVIIDIYGK-------PDEIRVELARELKKNAKEREELTKSIAQTTKAHEEYK---TLLQ-------------------------------------------------TEFGLTNVSRTDILRYKLYKELESCGYKTLYSNTYISREKLFSK--EFDIEHIIPQAR-LFDDSFSNKTLEARSVNIEKGNK-------------TAYDFVKEKFGESGADNSLEHYLNNIEDLFKSGK-----------------------------------ISKTKYNKLKMAEQDIPDGFIERDLRN-----TQYIAKKALSMLNEISHR---------------------VVATSGSVTDKLREDWQLIDVMKELNWEKYKALGLVEYFE-----DRDGR-------------------------------------QIGRIKDWTK-RNDHRHHAMDA-----LTVAFTKDVFIQYFNNKNASLDPNAN-------------------EHAIKNKYFQNGRAIAP---------MPLREFRAEAKKH------------------------------------------LENTLISIKAKNKVITGNINKTRK------KGGVNKNM--QQTPRGQLHLETIYGSGKQYLTKEEKVNASFDMRKIGTVSKSAYRDALLKRLYENDNDPKKAFAGKN-SLDKQPIWLDKEQMRKVPEKVKI-VTLEAIYTIRKEISPDLKVDKVIDVGVRKILIDRLNEYGNDAKKAFSNLDKNPIWLNKEKGISIKRVTISGISNAQSLHVKKDKDGKPIL----------------DENGRNIPVDFVNTGNNHHVAVYYRPVIDKRGQLVVDEAGNPK-----YELEEVVVSFFEAVTRANLGLPIIDKDYKTTEG--------------------WQFLFSMKQNEYFVFPNEKTG---FNPKEIDLLD-----------VENYGLISPNLFRVQKFSLKNYVFRHHLETTIKDTSSILR-------------GITWIDFRSSKGLDTIVKVRVNHIGQIVSVGEY--------------------

>gi|319419610|gb|ADV46720.1|_CRISPR-associated_protein_Csn1_family_Nitratifractor_salsuginis_DSM_16511

M-------------------------------------------------------------------KKILGVDLGITSFGYAILQET----------GKDLYRCLDNSVVMRNN------PYDEKSGESSQSIRSTQKSMRRLIEKRKKRIRCVAQTMERYGILDYSET----------------------------------------------------------MKINDPKNNPIKNRWQLRAVDAWKRPLSPQ-----ELFAIFAHMAKHRGYKSIATEDLIYELELELG-------------------------------LNDPEKESEKKADERRQ--------------------------------------------------------------------------------------------------------VYNALRHLEELRKKYGGETIAQTIHRAVEAGDLRSYRN-----------------------HDDYEKMIRREDIEEEIEKVLLRQAELGALGLPEEQVSELIDELK---------------------------ACITDQEMPTIDESLFGKCTFYKDEL--------------------------AAPAYSYLYDLYRLYKKLADLNIDGYE----------------------------------------------------VTQEDRE--------KVIEWVEKKIAQGKNLKKITHKDLRKILGLAPEQKIFGVEDER-------------IVKGKKEPRTFVPFFFLADIAKFKELFASIQKHP---------------------------------------------DALQIFRELAEILQRSKTPQEALDRLRALMAGKGIDTDDRELLELFKNKRSGTR--------------ELSHRYILEALPLFLEG------------------------YDEK-----------------------------------------EVQRILGFDDREDYSRYPKSLRHLHLREGNLFEKEENPINN------------HAVKSLASWALGLIADLSWRYGP-------FDEIILETTRDALPEKIRKEIDKAMREREKALDKIIG----------------------------------------------------------KYKKEFPSIDKRLARKIQLWERQKGLDLYSGKVINLSQLLDG--SADIEHIVPQSLGGLSTDYN-TIVTLKSVNAAKGNR-------------LPGDWLAGNPD------YRERIGMLSEKGLIDWK-------------------------------------KRKNLLAQSLDEIYTENTHSKGIRA-----TSYLEALVAQVLKRYYPFPDPELRKN----------GIGVRMIPGKVTSKTRSLLGIK----------------------------------------------------------------------------SKSRETNFHHAEDA-----LILSTLTRGWQNRLHRMLRDNYGKSE---------------------AELKELWKKYMPHIEGLTLADYIDEAFRRFMSKGEES----------------------------------------LFYRDMFDTIRSISYWVDKKP------------------------LSASSHKETVYSSRHEVPTLRKNILEAFDSLNVIKDRHKLT--------------------------------------------------------------TEEFMKRYDKEIRQKLWLHRIGNTNDESYRAVEERATQIAQILTRYQLMDAQNDKEIDEKFQQALKELITSPIEVTG-------------------KLLRKMRFVYDKLNAMQIDRG-------------------------LVETDKNMLGIHISKGPNEKLIFRRMDVNNAHELQK---------------ERSGILCYLNEMLFIFNKKGLIHYGCLRSYLEKGQ----------GSKYIALFNPRFPANPKAQPSKFTSDSKIKQVGIGSATG--------------------IIKAHLDLDGHVRSYEVFGTLPEGSIEWFKEESGYGRVEDDPHH---

>gi|324027241|gb|ADY14000.1|_CRISPR-associated_protein_Csn1_family_Sphaerochaeta_globosa_str._Buddy

M---------------------------------------------SKKVSRRYEEQAQEICQRLGSRPYSIGLDLGVGSIGVAVAAYDPIKKQ------PSDLVFVSSRIFIPS---------------TGAAERRQKRGQRNSLRHRANRLKFLWKLLAERNLMLSYSEQDVP----------------------------------------------------DPARLRFEDAVVRANPYELRLKGLNEQLTLS------ELGYALYHIANHRGSSSVRTFLDEEKSS-------------------------------------DDKKLEEQQAMTEQLAK----------------------------------------------------------------------------------------------------------EKGISTFIEVLTAFNTNGLIGYRNSES-----------------------------VKSKGVPVPTRDIISNEIDVLLQTQKQFYQEILSDEYCDRIVS------------------------------AILFENEKIVPEA---GCCPYFPDEK--------------------------KLPRCHFLNEERRLWEAINNARIKMPMQEG----------------------------------------AAKRYQSASFSDEQRH--------ILFHIARSG-------TDITPKLVQKEFPALKTSIIVLQGKEK---------------------AIQKIAGFRFRRLEEKSFWKRLSEE--------------------------------------------------QKDDFFSAWTNTPDDKRLSKYLMKHLLLT---ENEVVDALKTVSLIGDYG--------------PIGKTATQLLMKHLED----------------------GLTYTEALERGMETGEFQELSVWE-------QQSLLPYYGQILTGSTQALMGKYWHSAFKEKRDSEGFFKPNTNSDEEKYGRIANP----------------VVHQTLNELRKLMNELITILGAK------PQEITVELARELKVGAEKREDIIKQQTKQEKEAVLAYS----------------------------------------------------------KYCEPNNLDKRYIERFRLLEDQAFVCPYCLEHISVADIAAG--RADVDHIFPRDD-TADNSYGNKVVAHRQCNDIKGKR-------------TPYAAFSNTS-------AWGPIMHYLDETPGMWRK----------------------------------RRKFETNEEEYAKYLQSKGFVSRFESD-----NSYIAKAAKEYLRCLFNPNN----------------VTAVGSLKGMETSILRKAWNLQGIDDLLGSRHWSKDADTSP-------------------------------------------------------TMRKNRDDNRHHGLDA-----IVALYCSRSLVQMINTMSEQGKR--------------------------AVEIEAMIPIPGYASEPNLSFEAQRELFRKKILEFMD-----------------------------------------LHAFVSMKTDND------------------------------ANGALLKDTVYSILGADTQGEDLVFVVKKKIKDIGVKIGDYEEVAS--------------------------------------------------------AIRGRITDKQPKWYPMEMKDKIEQLQSKNEAALQKYKESLVQAAAVLEESNRKLIESGKKPIQLSEKTISKKALELVGG---------YYYLISNNKRTKTFVVKEPSNEVKGFAFDTG------------------------SNLCLDFYHDAQGKLCGEIIRKIQAMNPSYKPAYMKQG-------------YSLYVRLYQGDVCELRASDLTEAESNLAKTTHVRLPN--------AKPGRTFVIIITFTEMGSGYQIYFSNLAKSKKGQDTS---------------------FTLTTIKNYDVRKVQLSSAGLVRYVSPLLVDKIEKDEVALCGE-----

>gi|916811691|ref|WP_051418747.1|_type_II_CRISPR_RNA-guided_endonuclease_Cas9_Methylosinus_trichosporium

M--------------------------------------------------------------------------LGPVELAVALGH---------------IARHRGFKSNSKG----------AKTNDPADDTSKMKRAVNETREKLARFGSAAKMLVED------------------------------------------------------------------------------------ESFVLRQTPTKN------GASEIVRRFRNREG-----------------------------------------------------------------------------------------------------------------------------------------------------------------------------------------------------------------------------------------DYSRSLLRDDLAAEMRALFTAQARFQSAIATADLQTAFT-------------------------------KAAFFQRPLQDSEKLVGPCPFEVDEK--------------------------RAPKRGYSFELFRFLSRLNHVTLRDGK------------------------------------------------QERTLTRDELALAAADFGAAAKVSFTALR------KKLKLPETTVFVGVKADEESKLDVVAR----------------SGKAAEGTARLRSVIVDALGELAWGALLCS------------------------------------------------PEKLDKIAEVISFRSDIGRISEGLAQAGCNAPLVDALTAAASDGRFDPFTGAG-------------HISSKAARNILSGLR----------------------QGMTYDKACCAADY-----------------------------------DHTASRERGAFDVGGHGREALKRILQEERISRELVGSP----------------TARKALIESIKQVKAIVERYGV-------PDRIHVELARDVGKSIEEREEITRGIEKRNRQKDKLRGLFEKEVGRP------------------------------------------------PQDGARGKEELLRFELWSEQMG---RCLYTDDYISPSQLVATDDAVQVDHILPWSR-FADDSYANKTLCMAKANQDKKGR-------------TPYEWFKAEKT----DTEWDAFIVRVEALADMKG-------------------------------------FKKRNYKLRNAEEAAAKFRNRNLND-----TRWACRLLAEALKQLYPKGEKDKDGK---------ERRRVFSRPGALTDRLRRAWGLQWMKKS---------------------------------------------------------------------TKGDRIPDDRHHALDA-----IVIAATTESLLQRATREVQEIEDK--------------------------GLHYDLVKNVTP----------PWPGFREQAVEA------------------------------------------VEKVFVARAERRR------------------------------ARGKAHDATIRHIAVREGEQRVYERRKVAELKLADLDRVK--------------------------------------------------------------------DAERNARLIEKLRNWIEAGSPKDDPPLSPKGDPIFKVRLVTKSKVNIALDTGNPKRPGTVDRG-------------------------EMARVDVFRKASKKGKYEYYLVPIYP-----------------------HDIATMKTPPIRAVQAYKPEDEWPEMDSSYE----------------------FCWSLVPMTYLQVISSKGE-----------------------------IFEGYYRGMNRSVGAIQLSAHSNSSDVVQG-------------------------IGARTLTEFKKFNVDRFGRKHEVERELRTWRGETWRGKAYI-----

>gi|496302655|ref|WP_009013303.1|_hypothetical_protein_Prevotella_sp._C561

M------------------------------------------------------------------TQKVLGLDLGTNSIGSAVRN-----------LDLSDDLQWQLEFFSSDIFRSSVNKESNGREYSLAAQRSAHRRSRGLNEVRRRRLWATLNLLIKHGFCPMSSESLMRWCTYD----------------------------KRKGLFREYPIDDKDFNAWILLDFNGDGRPDYSSPYQLRRELVTRQFDFEQPIERYKLGRALYHIAQHRGFKSSKGETLSQQETNSKP--------------------------------SSTDEIPDVAGAMKAS-----------------------------------------------------------------------------------------------------------EEKLSKGLSTYMKEHNLLTVGAAFAQLEDEGVRVR------------------------NNNDYRAIRSQFQHEIETIFKFQQGLSVESELYERLISEKKN----------------------------VGTIFYKRPLRSQRGNVGKCTLERSKP--------------------------RCAIGHPLFEKFRAWTLINNIKVRMSVDTLDEQLPMKLRLDLYNECFLAFVRTEFKFEDIRKYLEKRLGIHFSYNDKTINYKDSTSVAGCPITARFRKMLGEEWESFRVEGQKERQAHSKNNISFHRVSYSIEDIWHFCYDAEEPEAVLAFAQETLRLERKKAEELVRIWSAMPQGYAMLSQKAIRN--------------------------------INKILMLGLKYSDAVILAKVPELVDVSDEELLSIAKDYYLVEAQVNYDKRINSIVNGLIAKYKSVSEEYRFADHNYEYLLDESDEKDIIRQIENSLGARRWSLMDANEQTDILQKVRDRYQDFFRSHERKFVESPKLGES--------FENYLTKKFPMVEREQWKKLYHPSQITIYRPVSVGKDRSVLRLGNPDIGAIKNP----------------TVLRVLNTLRRRVNQLLDDGVISPD----ETRVVVETARELNDANRKWALDTYNRIRHDENEKIKKILEEFYPKRDGISTDDIDKARYVID---------------------------QREVDYFTGSKTYNKDIKKYKFWLEQG---GQCMYTGRTINLSNLFDPN-AFDIEHTIPESL-SFDSSDMNLTLCDAHYNRFIKKN------------HIPTDMPNYDKAITIDGKEYPAITSQLQRWVERVERLNRNVEYWKGQARRAQNKDRKDQCMREMHLWKMELEYWKKKLERFTVTEVTDGFKNSQLVD-----TRVITRHAVLYLKSIFP---------------------HVDVQRGDVTAKFRKILGIQSVDE------------------------------------------------------------------------KKDRSLHSHHAIDATTLTIIPVSAKRDRMLELFAKIEEINKMLSFSG----------------------SEDRTGLIQELEGLKNKLQMEVKVCRIGHNVSEIG--------------------------------------TFINDNIIVNHHIKNQALTPVRRRLRKKGYIVG-GVDNPRWQTGDALRGEIHKASYYGAITQFAKDDEGKVLMKEGRPQVNPTIKFVIRRELKYKKSAADSG--------------------------------------------FASWDDLGKAIVDKELFALMKGQFPAETSFKDACEQGIYMIKKGKNGMPDIKLHHIRHVRCEAPQSGLKIKEQTYKSEK--------------------EYKRYFYAAVGDLYAMCCYTNG----------------------KIREFRIYSLYDVSCHRKSDIEDIPEFITDKKGNR------------------LMLDYKLRTGDMILLYKDNPAELYDLDNVNLSRRLYKIN---RFESQSNLVLMTHHLSTSKERGRSLGKTVDYQNLPESIRS-------------------SVKSLNFLIMGENRDFVIKNGKIIFNHR----------------------

>gi|281306166|gb|EFA98204.1|_CRISPR-associated_protein_Csn1_family_Prevotella_timonensis_CRIS_5C-B1

M------------------------------------------------------------------NKRILGLDTGTNSLGWAVVDWDEHAQS-------YELIKYGDVIFQEG------VKIEKGIESSKAAERSGYKAIRKQYFRRRLRKIQVLKVLVKYHLCPYLSDDDLR----------------------------------------QWHLQKQYPKSDELMLWQRTSDEEGKNPYYDRHRCLHEKLDLTVEADRYTLGRALYHLTQRRGFLSNRLDTSADNK--------------------------------------EDGVVKSGISQLSTE----------------------------------------------------------------------------------------------------------MEEAGCEYLGDYFYKLYDAQG-----------------------------------------NKVRIRQRYTDRNKHYQHEFDAICEKQELSSELIEDLQR-------------------------------AIFFQLPLKSQRHGVGRCTFERGKP--------------------------RCADSHPDYEEFRMLCFVNNIQVKGPHD----------------------------------------------LELRPLTYEERE--------KIEPLFFRKSK-----PNFDFEDIAKALAGKKNYAWIHDKEERAYKFN--------YRMTQGVPGCPTIAQLKSIFGDDWKTGIAETYTLIQ-----------------------------------------KKNGSKSLQEMVDDVWNVLYSFSSVEKLKEFAHHKLQLDEESAEKFAKIKLSHSFA--------------ALSLKAIRKFLPFLRKGMYYTHASFFANIPTIVGKEIWNKEQNRKYIMENVGELVFNYQPKHREVQGTIEMLIKDFLANNFELPAGATDKLYHPSMIETYPNAQRNEFGILQLGSPRTNAIRNP----------------MAMRSLHILRRVVNQLLKESIIDEN-----TEVHVEYARELNDANKRRAIADRQKEQDKQHKKYGDEIRKLYKEETG------------------------------------------------KDIEPTQTDVLKFQLWEEQN---HHCLYTGEQIGITDFIGSNPKFDIEHTIPQSV-GGDSTQMNLTLCDNRFNREVKKAKLPTELANHEEILTRIEPWKNKYEQLVKERDKQRTFAGMDKAVKDIRIQKR-------------------------HKLQMEIDYWRGKYERFTMTEVPEGFSRRQGTG-----IGLISRYAGLYLKSLFHQADSRNK-------------SNVYVVKGVATAEFRKMWGLQSEYEK------------------------------------------------------------------------KCRDNHSHHCMDA-----ITIACIGKREYDLMAEYYRMEETFK-------------------------QGRGSKPKFSKP-----------WATFTEDVLNIY------------------------------------------KNLLVVHDTPNNMPKHTKKYVQTSIG--------KVLAQGDTARGSLHLDTYYGAIERDGEIRYVVRRPLSSFTKPEELEN-----------------------------------------------------------------------IVDETVKRTIKEAIADKNFKQAIAEPIYMNEEKGILIKKVRCFAKSVKQPINIRQHRDLSKKE---------------------------YKQQYHVMNENNYLLAIYEGLVKN------------------KVVREFEIVSYIEAAKYYKRSQDRNIFSSIVPTHSTKYG---------------LPLKTKLLMGQLVLMFEENPDEIQVDNTKDLVKRLYKVVG----IEKDGRIKFKYHQEARKEGLPIFSTPYKNNDDYAPIFR--------------------QSINNINILVDGIDFTIDILGKVTLKE----------------------

>gi|281304649|gb|EFA96738.1|_CRISPR-associated_protein_Csn1_family_Prevotella_timonensis_CRIS_5C-B1

M-------------------------------------------------------------------KNILGLDLGSNSVGWALVK---VEENG---NPIGSIKMG-SRIIPMS--QDILGSFEKGDTVSQTAQRTLYRGKRRLIERHVLRRERLCRVLHIMNFLPAHFDRLLGWDK-TDNKTYGKFIDDSEPKLAWRQNKNEMGKMEFVFMDSFHEMLSDFAKHQPQLIANGKKVPLDWTIYYLRKKALTQLISKE------ELAWILLNFNKKRGYYQLRGEEEEEQPTKKEEYKVLKVISVDADEGQKGNGIWYNIHLEDGGIYKKKSDIPLYDWVGKTLQLIVVTTYEKDGKTPKIFDSGKQNP------------------------------------------------------------------------SYRLPKEDDWGLVKKRTESQLQSSGKTVGAFIYDNILSKPDDKIRGNLVRTIERKYYKKELIEILQQQAKYHDELHNQNLLESCAKELYAHNDVHYNEVIKSNMINLLVN------------------------------DIIFYQRPLKSKKSLIANCKYESYEYVDKETGE------------IKEMPIKCIAKSNPYYQEFRLWQFIHNLRLSDVVT------------------------------------------REDVTAQYLSTPQDYC--------NLFTYLNDR-------KEIKQDILLKDFFKIKKVQIDKEKVFPIEWNY-------IKDATKSYPCNETRYELLSALKRAGMDKQWLD-D-----------------------------------------------TPNMQYRLWHLLYSVEEKEESAKALRKLYD----DDKFVTSFLKIKPFNKCYG--------------AYSEKAIKRLLTLMRMGKAWN---------EQAIDAKTLKCIQQIITDNVDSKLKEKIDDSR-------YVLHHVSDFQGLPTYLASYVIYGRHSEVTDIQCWDSPEDLLKFIRDFKQHSLRNP----------------IVEQVILETLRVVYDIWKAEKH-------IDEIHVEVAREMKLTAQQRNEANLRNLNNEATNLRIK---YLLEELKNDTF-IKDVRPLSPIHQEKMRIYEQAVLGNLNKQDKDYDDII----KISKKEHPTNAELIRYKLWLEQR---YCSPYTGKAISLSKLFTT--AYEIEHIIPQSR-YFDNSFNNKVICEAEVNKAKGNM-------------LGYEFIKKQGGQKIYCTQLGKAVTILKEEAYKQFVNE-------------------------------HYAHNKRKRDNLLAENIPESFSSRQLND-----TRYITKTIMSLLSNVVRQQSEEEA-----------TAKNLLPSSGSVTDRLKNDWGLKDVWNTIVTPRFERLNKLSGTNDFGEMKEENGNRYFQTNV-----------------------------PLEYEKGFKKKRIDHRHHAMDA-----LVIACTTRNMVNYISNANA--NSPKQRED--LRQLLC-------DKNR---------IINKP---------WDT--FTQDALKA------------------------------------------LNDIVVSFKNNVRIINRATNRYQRYDKNGKKIIFSQKGDDMWAIRKSMHKETVFG--RVNLIRKEVLPIAKALDNISAICNAQLRAYVNDLVDKHFNKKQLVAHFKSLNYKWNRQDVSKVEVWISS-DNKT-PMVAVRKPLDTSFDKKKIAS-ITDTGIQTILLNYLESKGGDASVAFAPEGIQEMNSNIELYNNGKAHKPIKNVRLSEPLGAKYQVG--------------------ERGAKTKKYVEADKGTNLFFAIYE------------DEEG---------KRYYYSVPLREAIERQKQKLSPVPEYNEKGVA----------------------LKFYLSPNDLVYVPTEEER---TMG--TCHIDK----------ARIYKMVSSTGCSCYFVPSTFAKVIVDKVELQS-------------------LNKMEKAITDECIKEVCWKLKVDRLGNIINVIK---------------------

>gi|292665686|gb|ADE40787.1|_CRISPR-associated_protein_Csn1_family_Candidatus_Puniceispirillum_marinum_IMCC1322

M--------------------------------------------------------------------RRLGLDLGTNSIGWCLLD---LGDDG----EPVSIFRTGARIFSDG--------RDPKSLGSLKATRREARLTRRRRDRFIQRQKNLINALVKYGLMPADEI-------------------------------------------------------------QRQALA-YKDPYPIRKKALDEAIDPY------EMGRAIFHINQRRGFKSNRKS--ADN---------------------------------------EAGVVKQSIADLEMK----------------------------------------------------------------------------------------------------------LGEAGARTIGEFLADRQATN--------DTVRAR-------RLSGT-------------NALYEFYPDRYMLEQEFDTLWAKQAAFNPSLYIEAARERLK-------------------------------EIVFFQRKLKPQE--VGRCIFL--------------------------SDEDRISKALPSFQRFRIYQELSNLAWIDHDG---------------------------------------------VAHRITASLALRD--------HLFDELEHK-------KKLTFKAMRAILRKQGVVDYPVGFNLE-------------SDNRDHLIGNLTSCIMRDAKKMIGSAWDRLDEE-----------------------------------------------EQDSFILM--LQDDQKGDDEVRSILTQQYG----LSDDVAEDCLDVRLPDGHG--------------SLSKKAIDRILPVLR---------------------DQGLIYYDAVKEAGLGEAN-----------------LYDPYAALS-------DKLDYYGKALAGHVMGASGKFEDS-DEKRYGTISNP----------------TVHIALNQVRAVVNELIRLHGK-------PDEVVIEIGRDLPMGADGKRELERFQKEGRAKNERAR---DELKKLG------------------------------------------------HIDSRES-RQKFQLWEQLAKEPVDRCCPFTGKMMSISDLFSD--KVEIEHLLPFSL-TLDDSMANKTVCFRQANRDKGNR-------------APFDAFGNSP----AGYDWQEILGRSQNLPYAKR-----------------------------------WRFLPDAMKRFEADG---GFLERQLND-----TRYISRYTTEYISTIIPKN-------------------KIWVVTGRLTSLLRGFWGLNSILRG----HNTDDG----------------------------------------------------------TPAKKSRDDHRHHAIDA-----IVVGMTSRGLLQKVSKAARRSE----------------------------DLDLTRLFEGRI---------DPWDGFRDEVKKH------------------------------------------IDAIIVSHRPRKK------------------------------SQGALHNDTAYGIVEH-AE-----NGASTVVHRVPITSLG---------------------------------------------------------------KQSDIEKVRDPLIKSALLNETAGLSGKSFENAVQKW-CADNSIKSLRIVETVSIIPIT----DKEGV--------------------------------AYKGYKGDGNAYMDIYQDP----------TSSK----------WKGEIVSRFDAN---------QKGFIPSWQSQFPTA----------------RLIMRLRINDLLKLQDGEIE---EIYRVQRLS------------GS--KILMAPHTEANVDARDRD--KNDTFKLTS-------------------------KSPGKLQSASARKVHISPTGLIREG-----------------------

>gi|329308217|gb|AEB82632.1|_CRISPR-associated_protein_Csn1_family_Alicycliphilus_denitrificans_K601

M----------------------------------------------------------------RSLRYRLALDLGSTSLGWALFR---LDACN----RPTAVIKAGVRIFSDG--------RNPKDGSSLAVTRRAARAMRRRRDRLLKRKTRMQAKLVEHGFFPADAG-------------------------------------------------------------KRKALE-QLNPYALRAKGLQEALLPG------EFARALFHINQRRGFKSNRKTDKKDN---------------------------------------DSGVLKKAIGQLRQQ----------------------------------------------------------------------------------------------------------MAEQGSRTVGEYLWTRLQQG--------QGVRARYREKPYTTEEGKK----------RIDKSYDLYIDRAMIEQEFDALWAAQAAFNPTLFHEAARADLK-------------------------------DTLLHQRPLRPVK--PGRCTLL--------------------------PEEERAPLALPSTQRFRIHQEVNHLRLLDEN----------------------------------------------LREVA-LTLAQRD--------AVVTALETK-------AKLSFEQIRKLL--KLS--GSVQFNLE-------------DAKRTELKGNATSAAL-ARKELFGAAWSGFDEA-----------------------------------------------LQDEIVWQ--LVTEE-GEGALIAWLQTHTG----VDEARAQAIVDVSLPEGYG--------------NLSRKALARIVPALR---------------------AAVITYDKAVQAAGFDHHSQLGFEYD-------ASEVEDLVHPETGEIRSVFKQLPYYGKALQRHVAFGSGKPEDP-DEKRYGKIANP----------------TVHIGLNQVRMVVNALIRRYGR-------PTEVVIELARDLKQSREQKVEAQRRQADNQRRNARIRRSIAEVLGIG------------------------------------------------EERVRGSDIQKWICWEELSFDAADRRCPYSGVQISAAMLLSD--EVEVEHILPFSK-TLDDSLNNRTVAMRQANRIKRNR-------------TPWDARAEFEA---QGWSYEDILQRAERMPLRKR-----------------------------------YRFAPDGYERWLGDDK--DFLARALND-----TRYLSRVAAEYLRLVCPG---------------------TRVIPGQLTALLRGKFGLNDVLG------LDGE---------------------------------------------------------------KNRNDHRHHAVDA-----CVIGVTDQGLMQRFATASAQAR----------------------------GDGLTRLVDGMP---------MPWPTYRDHVERA------------------------------------------VRHIWVSHRPDHG------------------------------FEGAMMEETSYGIRK---------DGSIKQRR--------------------------------------------------------------------------------------------KADGSAGREISN-----------LIRIHEATQPLRHG-VSADGQPL--------------------------------AYKGYVGGSNYCIEITVN-----------DKGK----------WEGEVISTFRAYGVVR---AGGMGRLRNPHEGQNGR----------------KLIMRLVIGDSVRLEVDGAE---RTMRIVKISGS----------NG--QIFMAPIHEANVDARNTD--KQDAFTYTS-------------------------KYAGSLQKAKTRRVTISPIGEVRDPGFKG-------------------

>gi|344171927|emb|CCA84553.1|_conserved_hypothetical_protein_Ralstonia_syzygii_R24

M----------------------------------------------------------------AEKQHRWGLDIGTNSIGWAVIA---LIEG-----RPAGLVATGSRIFSDG--------RNPKDGSSLAVERRGPRQMRRRRDRYLRRRDRFMQALINVGLMPGDAA-------------------------------------------------------------ARKALV-TENPYVLRQRGLDQALTLP------EFGRALFHLNQRRGFQSNRKTDRATAK--------------------------------------ESGKVKNAIAAFRAG----------------------------------------------------------------------------------------------------------MGN--ARTVGEALARRLEDG--------RPVRAR------MVGQGK-------------DEHYELYIAREWIAQEFDALWASQQRFHAEVLADAARDRLR-------------------------------AILLFQRKLLPVP--VGKCFLE--------------------------PNQPRVAAALPSAQRFRLMQELNHLRVMTLAD---------------------------------------------KRERP-LSFQERN--------DLLAQLVAR-------PKCGFDMLRKIV--FGANKEAYRFTIE-------------SERRKELKGCDTAAKL-AKVNALGTRWQALSLD-----------------------------------------------EQDRLVCL--LLDGE-NDAVLADALREHYG----LTDAQIDTLLGLSFEDGHM--------------RLGRSALLRVLDALESGRDEQ---------------GLPLSYDKAVVAAGYPAH------------------TADLENGER-------DALPYYGELLWRYTQDAP-TAKND-AERKFGKIANP----------------TVHIGLNQLRKLVNALIQRYGK-------PAQIVVELARNLKAGLEEKERIKKQQTANLERNERIR---QKLQDAG------------------------------------------------VPDNRENRLRMRLFEELGQGNGLGTPCIYSGRQISLQRLFSN--DVQVDHILPFSK-TLDDSFANKVLAQHDANRYKGNR-------------GPFEAFGANR----DGYAWDDIRARAAVLPRNKR-----------------------------------NRFAETAMQDWLHNET--DFLARQLTD-----TAYLSRVARQYLTAICSKD-------------------DVYVSPGRLTAMLRAKWGLNRVLDG----VMEEQG----------------------------------------------------------RPAVKNRDDHRHHAIDA-----VVIGATDRAMLQQVATLAARAR----------------------------EQDAERLIGDMP---------TPWPNFLEDVRAA------------------------------------------VARCVVSHKPDHG------------------------------PEGGLHNDTAYGIVAGPFE-----DGRYRVRHRVSLFDLK---------------------------------------------------------------PGDLSNVRCDAPLQAELEPIFEQDDARAREVALTAL-AERYRQRKVWLEELMSVLPIRPRGEDGKTLPDSA----------------------------PYKAYKGDSNYCYELFIN-----------ERGR----------WDGELISTFRANQAAYRRFRNDPARFRRYTAG--GR----------------PLLMRLCINDYIAVGTAAER---TIFRVVKMS-E----------NK--ITLAEHFEGGTLKQRDAD--KDDPFKYLT-------------------------KSPGALRDLGARRIFVDLIGRVLDPGIKGD------------------

>gi|157910716|gb|ABV92149.1|_CRISPR-associated_protein_Dinoroseobacter_shibae_DFL_12___DSM_16493

M---------------------------------------------------------------------RLGLDIGTSSIGWWLYE---TDGAGSDARITGVVDG-GVRIFSDG--------RDPKSGASLAVDRRAARAMRRRRDRYLRRRATLMKVLAETGLMPADPA-------------------------------------------------------------EAKALE-ALDPFALRAAGLDEPLPLP------HLGRALFHLNQRRGFKSNRKTDRGDN---------------------------------------ESGKIKDATARLDME----------------------------------------------------------------------------------------------------------MMANGARTYGEFLHKRRQKATDPRHVPSVRTRLS------IANRGGPD--------GKEEAGYDFYPDRRHLEEEFHKLWAAQGAHHP-ELTETLRDLLF-------------------------------EKIFFQRPLKEPE--VGLCLFSGHHG--------------------VPPKDPRLPKAHPLTQRRVLYETVNQLRVTADG-----------------------------------------------REARPLTREERDQVIH---ALDNKKPTKSLSS----MVLKLPALAKVLK----LRDGERFTLE-------------TGVRDAIAC-DPLRASPAHPDRFGPRWSILDAD-----------------------------------------------AQWEVISRIRRVQSDAEHAALVDWLTEAHG----LDRAHAEATAHAPLPDGYG--------------RLGLTATTRILYQLT---------------------ADVVTYADAVKACG-------------------------WHHSDG-RTGECFDRLPYYGEVLERHVIPGSYHPDDD-DITRFGRITNP----------------TVHIGLNQLRRLVNRIIETHGK-------PHQIVVELARDLKKSEEQKRADIKRIRDTTEAAKKRS---EKLEELE------------------------------------------------IEDNGRNRMLLRLWEDLNPDDAMRRFCPYTGTRISAAMIFDG--SCDVDHILPYSR-TLDDSFPNRTLCLREANRQKRNQ-------------TPWQAWG-DTP------HWHAIAANLKNLPENKR-----------------------------------WRFAPDAMTRFEGEN---GFLDRALKD-----TQYLARISRSYLDTLFTKGG------------------HVWVVPGRFTEMLRRHWGLNSLLS------DAGRG----------------------------------------------------------AVKAKNRTDHRHHAIDA-----AVIAATDPGLLNRISRAAGQGE--------------------------AAGQSAELIARDTP---------PPWEGFRDDLRVR------------------------------------------LDRIIVSHRADHGRIDHAARKQGRDS-----------------TAGQLHQETAYSIVDDIHVA----SR-TDLLSLKPAQLLD---------------------------------------------------------------EPGRSGQVRDPQLR------KALRVATGGKTGKDFENALRYFASKPGPYQAIRRVRIIKPLQAQARVPVP-------------------------AQDPIK-AYQGGSNHLFEIWRLP----------DGE-----------IEAQVITSFEAHTLEGEK---R---PHPAA----------------------KRLLRVHKGDMVALE-RDGR---RVVGHVQKMDI----------AN-GLFIVPHN-EANADTRNND--KSDPFKWIQ-------------------------IGARPAIASGIRRVSVDEIGRLR--DGGTRPI----------------

>gi|83591793|ref|YP_425545.1|_CRISPR-associated_endonuclease_Csn1_family_protein_Rhodospirillum_rubrum_ATCC_11170

M---------------------------------------------------------------RPIEPWILGLDIGTDSLGWAVFS---CEEKGP--PTAKELLGGGVRLFDSGR--------DAKDHTSRQAERGAFRRARRQTRTWPWRRDRLIALFQAAGLTPPAAET-------------------------------------------------------------------RQIALALRREAVSRPLAPD------ALWAALLHLAHHRGFRSNRIDKRERAAAKALAKAKP---------------------------AKATAKATAPAKEADDEAGFWEG--------------------------------------------------------------------------------------------AEAALRQRMAASGAPTVGALLADDLDRGQPVRMRYNQS-----------------------------DRDGVVAPTRALIAEELAEIVARQSSAYPGLDWPAVTR-----------------------------------LVLDQRPLRSKG--AGPCAFLPGED--------------------------RALRALPTVQDFIIRQTLANLRLPSTSAD----------------------------------------------EPRPLTDEEHA--------KALALLSTAR-------FVEWPALRRALGLKRGVKFTAETERN---------------GAKQAARGTAGNLTEAILAPLIPGWSGWDLD-------------------------------------------------RKDRVFSDLWAARQDRSALLALIGDPRGPTRVTEDETAEAVADAIQIVLPTG-----------RASLSAKAARAIAQAMAP----------------------GIGYDEAVTLALGLHHS-------------------HRPRQERLARLPYYAAALPDVGLDGDPVGPPPAEDDGAAAEAYYGRIGNIS----------------VHIALNETRKIVNALLHRHGPI------LRLVMVETTRELKAGADERKRMIAEQAERERENAEIDVELRKSD----------------------------------------------------RWMANARERRQRVRLARRQN---NLCPYTSTPIGHADLLGD--AYDIDHVIPLAR-GGRDSLDNMVLCQSDANKTKGDK-------------TPWEAFHDKPG---WIAQRDDFLARLDPQTAKALAWR-------------------------------FADDAGERVARKSAEDEDQGFLPRQLTD-----TGYIARVALRYLSLVTNEPN------------------AVVATNGRLTGLLRLAWDITPGPAPRDLLPTPRDALRDDTAARRFLDGLTPPPLAKAVEGAVQARLAALGRSRVADAGLADA---LGLTLASLGGGGKNRADHRHHFIDA-----AMIAVTTRGLINQINQASGAGR--------------------------ILDLRKWPRTNFEP----------PYPTFRAEVMKQ------------------------------------------WDHIHPSIRPAHR------------------------------DGGSLHAATVFGVRNRPDARVLVQRKPVEKLFLDANAKPLP---------------------------------------------------------------ADKIAEIIDGFASPRMAKRFKALLARYQAAHPEVPPALAALAVARDPAFGPRGMTANTVIAGRSDGDGEDAGLITP---------------FRANPKAAVRTMGNAVYEVWEIQVKGR---------------------PRWTHRVLTRFDRTQPAPPPPPENAR-----------------------------LVMRLRRGDLVYWPLESGD----RLFLVKKMAV----------DGRLALWPARLATGKATALYAQLSCPNINLNGDQG--------------------YCVQSAEGIRKEKIRTTSCTALGRLRLSKKAT-------------------

>gi|288910049|dbj|BAI71538.1|_CRISPR-associated_protein_Csn1_family_Azospirillum_sp._B510

MARPAFRAPRREHVNGWTPDPHRISKPFFILVSWHLLSRVVIDSSSGCFPGTSRDHTDKFAEWECAVQPYRLSFDLGTNSIGWGLLN---LDR----QGKPREIRALGSRIFSDG--------RDPQDKASLAVARRLARQMRRRRDRYLTRRTRLMGALVRFGLMPADPA-------------------------------------------------------------ARKRLEVAVDPYLARERATRERLEPF------EIGRALFHLNQRRGYKPVRTATKPDE---------------------------------------EAGKVKEAVERLEAA----------------------------------------------------------------------------------------------------------IAAAGAPTLGAWFAWRKTRG--------ETLRAR------LAGKG-------------KEAAYPFYPARRMLEAEFDTLWAEQARHHPDLLTAEAREILR-------------------------------HRIFHQRPLKPPP--VGRCTLY--------------------------PDDGRAPRALPSAQRLRLFQELASLRVIHLD-----------------------------------------------LSERPLTPAERDRIVA---FVQGRPPKAGRKPGKVQKSVPFEKLRGLLE----LPPGTGFSLE-------------SDKRPELLG-DETGARIAP--AFGPGWTALPLE-----------------------------------------------EQDALVE---LLLTEAEPERAIAALTARWA----LDEATAAKLAGATLPDFHG--------------RYGRRAVAELLPVLERETRGD---------------PDGRVRPIRLDEAVKLLRG-------------------GKDHSDFSREGALLDALPYYGAVLERHVAFGTGNPADP-EEKRVGRVANP----------------TVHIALNQLRHLVNAILARHGR-------PEEIVIELARDLKRSAEDRRREDKRQADNQKRNEERK---RLILSLG------------------------------------------------ERPTPRNLLKLRLWEEQGPVEN--RRCPYSGETISMRMLLSE--QVDIDHILPFSV-SLDDSAANKVVCLREANRIKRNR-------------SPWEAFGHDSE------RWAGILARAEALPKNKR-----------------------------------WRFAPDALEKLEGEG---GLRARHLND-----TRHLSRLAVEYLRCVCP---------------------KVRVSPGRLTALLRRRWGIDAILA------EADGPPPEVPAETL-----------------------------------------------DPSPAEKNRADHRHHALDA-----VVIGCIDRSMVQRVQLAAASAEREAA----------------------AREDNIRRVLEGFKE--------EPWDGFRAELERR------------------------------------------ARTIVVSHRPEHG------------------------------IGGALHKETAYGPV---DPP----EEGFNLVVRKPIDGLS---------------------------------------------------------------K-DEINSVRDPRLRRALIDRLAIRRRDANDPATALAKAAEDLAAQP-ASRGIRRVRVLKKESNPIRVEHGGNPS------------------GPRSGGPFHKLLLAGEVHHVDVALRA----------DGRR----------WVGHWVTLFEAHGGRGAD---G---AAAPPRLGDGE----------------RFLMRLHKGDCLKLEHKGRV---RVMQVVKLEPS----------SNSVVVVEPHQVKTDRSKHVKI--SCDQLRARG-------------------------ARRVTVDPLGRVRVHAPGARVGIGGDAGRTAMEPAEDIS---------

>gi|91802344|gb|ABE64718.1|_CRISPR-associated_endonuclease_Csn1_family_(plasmid)_Nitrobacter_hamburgensis_X14

M--------------------------------------HVEIDFPHFSRGDSHLAMNKNEILRGSSVLYRLGLDLGSNSLGWFVTH---LEKRGD-RHEPVALGPGGVRIFPDG--------RDPQSGTSNAVDRRMARGARKRRDRFVERRKELIAALIKYNLLPDDAR-------------------------------------------------------------ERRALE-VLDPYALRKTALTDTLPAH------HVGRALFHLNQRRGFQSNRKTDSKQS---------------------------------------EDGAIKQAASRLATDKGNETLGVFFADMHLRKSYEDRQTA------------------------------------------------------------IRAELVRLGKDHLTGNARKKIWAKVRKRLFGDEVLPRADAP--------HGVRAR------ATITG-------------TKASYDYYPTRDMLRDEFNAIWAGQSAHHA-TITDEARTEIE-------------------------------HIIFYQRPLKPAI--VGKCTLDPATRPF-----------------KEDPEGYRAPWSHPLAQRFRILSEARNLEIRDTG-----------------------------------------------KGSRRLTKEQSD--------LVVAALLAN-------REVKFDKLRTLLK----LPAEARFNLE-------------SDRRAALDG-DQTAARLSDKKGFNKAWRGFPPE-----------------------------------------------RQIAIVA---RLEETEDENELIAWLEKECA----LDGAAAARVANTTLPDGHC--------------RLGLRAIKKIVPIMQDGLDEDG--------------VAGAGYHIAAKRAG------------------------YDHAKLPTG--EQLGRLPYYGQWLQDAVV-GSGDARDQ-KEKQYGQFPNP----------------TVHIGLGQLRRVVNDLIDKYGP-------PTEISIEFTRALKLSEQQKAERQREQRRNQDKNKARA---EELAKFG------------------------------------------------RPANPRNLLKMRLWEE-LAHDPLDRKCVYTGEQISIERLLSD--EVDIDHILPVAM-TLDDSPANKIICMRYANRHKRKQ-------------TPSEAFGSSPTLQGHRYNWDDIAARATGLPRNKR-----------------------------------WRFDANAREEFDKRG---GFLARQLNE-----TGWLARLAKQYLGAVTDPN-------------------QIWVVPGRLTSMLRGKWGLNGLLPSDNYAGVQDKAEEFLASTDD-----------------------------------------------MEFSGVKNRADHRHHAIDG-----LVTALTDRSLLWKMANAY--------------------------------DEEHEKFVIEPP-----------WPTMRDDLKAA------------------------------------------LEKMVVSHKPDHG------------------------------IEGKLHEDSAYGFVKPLDATGLKEEEAGNLVYRKAIESLN---------------------------------------------------------------E-NEVDRIRDIQLRTIVRDHVNVEKTKGVALADALRQLQAPSDDYPQFKHGLRHVRILKKEKGDYLVPIAN-----------------------RASGVAYKAYSAGENFCVEVFETA----------GGK-----------WDGEAVRRFDANKKNAGP---KIAHAPQWRDANEGA----------------KLVMRIHKGDLIRLDHEGRA---RIMVVHRLDAA----------AG-RFKLADHNETGNLDKRHATNNDIDPFRWLM-------------------------ASYNTLKKLAAVPVRVDELGRVWRVMPN--------------------

>gi|908658930|ref|WP_049795381.1|_type_II_CRISPR_RNA-guided_endonuclease_Cas9_Bradyrhizobium_sp._BTAi1

M-------------------------------------------------------------------------DLGANSLGWFVVW---LDDHG----QPEGLGPGGVRIFPDG--------RNPQSKQSNAAGRRLARSARRRRDRYLQRRGKLMGLLVKHGLMPADEP-------------------------------------------------------------ARKRLE-CLDPYGLRAKALDEVLPLH------HVGRALFHLNQRRGLFANRAIEQGDK---------------------------------------DASAIKAAAGRLQTS----------------------------------------------------------------------------------------------------------MQACGARTLGEFLNRRHQLR--------ATVRAR------SPVGGD------------VQARYEFYPTRAMVDAEFEAIWAAQAPHHP-TMTAEAHDTIR-------------------------------EAIFSQRAMKRPS--IGKCSLDPATS-------------------QDDVDGFRCAWSHPLAQRFRIWQDVRNLAVVETG-----------------------------------------------PTSSRLGKEDQD--------KVARALLQT-------DQLSFDEIRGLLG----LPSDARFNLE-------------SDRRDHLKG-DATGAILSARRHFGPAWHDRSLD-----------------------------------------------RQIDIVA---LLESALDEAAIIASLGTTHS----LDEAAAQRALSALLPDGYC--------------RLGLRAIKRVLPLMEAGR----------------------TYAEAASAAG------------------------YDHALLPGGKLSPTGYLPYYGQWLQNDVV-GSDDERDT-NERRWGRLPNP----------------TVHIGIGQLRRVVNELIRWHGP-------PAEITVELTRDLKLSPRRLAELEREQAENQRKNDKRT---SLLRKLG------------------------------------------------LPASTHNLLKLRLWDE-QGD--VASECPYTGEAIGLERLVSD--DVDIDHLIPFSI-SWDDSAANKVVCMRYANREKGNR-------------TPFEAFGHR---QGRPYDWADIAERAARLPRGKR-----------------------------------WRFGPGARAQFEELG---DFQARLLNE-----TSWLARVAKQYLAAVTHPH-------------------RIHVLPGRLTALLRATWELNDLLP-----GSDDR-------------------------------------------------------------AAKSRKDHRHHAIDA-----LVAALTDQALLRRMANAH--------------------------------DDTRRKIEVLLP-----------WPTFRIDLETR------------------------------------------LKAMLVSHKPDHG------------------------------LQARLHEDTAYGTVEHPET-----EDGANLVYRKTFVDIS---------------------------------------------------------------E-KEIDRIRDRRLRDLVRAHVAGERQQGKTLKAAVLSFAQ-RRDIAGHPNGIRHVRLTKSIKPDYLVPIRD-----------------------KAG-RIYKSYNAGENAFVDILQAE----------SGR-----------WIARATTVFQANQANES-------------HDAPAA----------------QPIMRVFKGDMLRIDHAGAE---KFVKIVRLSPS----------NN-LLYLVEHHQAGVFQTRHDD--PEDSFRWLF-------------------------ASFDKLREWNAELVRIDTLGQPWRRKRGLETGSEDATRIGWTRPKKWP

>gi|499451825|ref|WP_011139289.1|_type_II_CRISPR_RNA-guided_endonuclease_Cas9_Wolinella_succinogenes

M------------------------------------------------------------------IERILGVDLGISSLGWAIVEYDKDDEAAN------RIIDCGVRLFTAAE--------TPKKKESPNKARREARGIRRVLNRRRVRMNMIKKLFLRAGLIQDVDLDGE--------------------------------------------------------GGMFYSKANRADVWELRHDGLYRLLKGD------ELARVLIHIAKHRGYKFIGDDEADEE----------------------------------------SGKVKKAGVVLRQN----------------------------------------------------------------------------------------------------------FEAAGCRTVGEWLWRERGANG--------KKRNKHG-------------------------DYEISIHRDLLVEEVEAIFVAQQEMRSTIATDALKAAYR-------------------------------EIAFFVRPMQRIEKMVGHCTYFPEER--------------------------RAPKSAPTAEKFIAISKFFSTVIIDNEG-----------------------------------------------WEQKIIERKTLEELL---DFAVSREKVEFRHLRKFLDLSDNEIFKGLHYKGKPKTAKKREATLFDPNEP----TELEFDKVEAEKKAWISLRGAAKLREALGNEFYGR--------------------------------------------FVALGKHADEATKILTYYKDEGQKRRELTKLPL------EAEMVERLVKIGFSDFL--------------KLSLKAIRDILPAMES----------------------GARYDEAVLMLG--------------------------------VPHKEKSAILPPLN-------------------KTDIDILNP----------------TVIRAFAQFRKVANALVRKYGA-------FDRVHFELAREINTKGEIEDIKESQRKNEKERKEAAD----WIAETS-------------------------------------------------FQVPLTRKNILKKRLYIQQD---GRCAYTGDVIELERLFDEG-YCEIDHILPRSR-SADDSFANKVLCLARANQQKTDR-------------TPYEWFGHDAA------RWNAFETRTSAPSNRVRTG---------------------------------KGKIDRLLKKNFDENSEMAFKDRNLND-----TRYMARAIKTYCEQYWVFKNSHTKAP-------------VQVRSGKLTSVLRYQWGLE----------------------------------------------------------------------------SKDRESHTHHAVDA-----IIIAFSTQGMVQKLSEYYRFKETHR-------------------------EKERPKLAVPLAN-FRDAVEEATRIENTETVKEG----------------------------------------VEVKRLLISRPPRAR------------------------------VTGQAHEQTAKPYPR-----------------------------------------------------------------------------------------IKQVKNKKKWRLAPIDEEKFESFKADRVASANQKNFYETSTIPRVDVYHKKGKFHLVPIYLHEMVLNELPN-----------------------LSLGTNPEAMDENFFKFSIFKDD-----------------------------LISIQTQGTPKKPAKIIMGYFKNMHGAN-------------------MVLSSINNSPCEGFTCTPVS--------MDKKHK----------DKCKLCPEENRIAGRCLQGFLDYWSQEGLRPPRKE---------------------FECDQGVKFALDVKKYQIDPLGYYYEVKQEKRLGTIPQMRSAKKLVKK--

>gi|218563121|ref|YP_002344900.1|_CRISPR-associated_protein_Campylobacter_jejuni_subsp._jejuni_NCTC_11168___ATCC_700819

M-------------------------------------------------------------------ARILAFDIGISSIGWAFSE-------------NDELKDCGVRIFTKVE--------NPKTGESLALPRRLARSARKRLARRKARLNHLKHLIANEFKLNYEDYQSF----------------------------------------------------DESLAKAYKGS--LISPYELRFRALNELLSKQ------DFARVILHIAKRRGYDDIKN-SDDKEK----------------------------------------GAILKAIKQNEEK----------------------------------------------------------------------------------------------------------LAN--YQSVGEYLYKEYFQKFKENSKEFTNVRNKKE-------------------------SYERCIAQSFLKDELKLIFKKQREFGFSFS--------------------------------KKFEEEVLSVAFYKRALKDFSHLVGNCSFFTDEK--------------------------RAPKNSPLAFMFVALTRIINLLNNLKN------------------------------------------------TEGILYTKDDL-------NALLNEVLKNG-------TLTYKQTK----KLLGLSDDYEFKG-EKGTY--------------FIEFKKYKEFIKALGEHNLSQD-------------------------------------------------------DLNEIAKDITLIKDEIKLKKALAKYDLN----QNQIDSLS--KLEFKDHL--------------NISFKALKLVTPLML----------------------EGKKYDEACNELNL-----------------------KVAINE------DKKDFLPAFN--------------ETYYKD---EVTNP----------------VVLRAIKEYRKVLNALLKKYGK-------VHKINIELAREVGKNHSQRAKIEKEQNENYKAKKDAE---LECEKLG----------------------------------------------LKINSK-----NILKLRLFKEQK---EFCAYSGEKIKISD-LQDEKMLEIDHIYPYSR-SFDDSYMNKVLVFTKQNQEKLNQ-------------TPFEAFG------NDSAKWQKIE--VLAKNLPTK-------------------------------------KQKRILDKNYKDKEQKNFKDRNLND-----TRYIARLVLNYTKDYLDFLPLSDDENTKLNDTQKGSKVHVEAKSGMLTSALRHTWGFS----------------------------------------------------------------------------AKDRNNHLHHAIDA-----VIIAYANNSIVKAFSDFKKEQESNSAELYAKKISE-------------LDYKNK-------------RKFFEPFSGFRQKVLDK------------------------------------------IDEIFVSKPERKK------------------------------PSGALHEETFRKEEE-----------------------------------------------------------------------------------------------------------FYQSYGGKEG---------------------------VLKALELGKIRKVNGKIVKN----------------GDMFRVDIFKHKKTNKFYAVPIYTMD------------------------FALKVLPNKAVARSKK-GEIKDWILMDENYE----------------------FCFSLYKDSLILIQTKDMQ--EPEFVYYNAFTS----------STVSLIVSKHDNKFETLSKNQKILFKNANE---KEVI--------------------AKSIGIQNLKVFEKYIVSALGEVTKAEFRQREDFKK-------------

>gi|290963459|emb|CBG39289.1|_Putative_CRISPR_associated_protein_Helicobacter_mustelae_12198

M-------------------------------------------------------------------IRTLGIDIGIASIGWAVIEGEYTDKGLE----NKEIVASGVRVFTKAE--------NPKNKESLALPRTLARSARRRNARKKGRIQQVKHYLSKALGLDLECFVQ-----------------------------------------------------GEKLATLFQTSKDFLSPWELRERALYRVLDKE------ELARVILHIAKRRGYDDITYGVEDNDS----------------------------------------GKIKKAIAENSKR----------------------------------------------------------------------------------------------------------IKEEQCKTIGEMMYKLYFQKS-------LNVRNKKE-------------------------SYNRCVGRSELREELKTIFQIQQELKSPWVNEELIYKLLGN-------------------PDAQSKQEREGLIFYQRPLKGFGDKIGKCSHIKKGEN----------------------SPYRACKHAPSAEEFVALTKSINFLKNLTN------------------------------------------------RHGLCFSQEDM-------CVYLGKILQEAQK--NEKGLTYSKLK----LLLDLPSDFEFLGLDYSGK--------------NPEKAVFLSLPSTFKLNKITQDR-----------------------------------------------------KTQDKIANILGANKDWEAILKELESLQLS----KEQIQTIKDAKLNFSKHI--------------NLSLEALYHLLPLMR----------------------EGKRYDEGVEILQE-----------------------RGIFSKP---QPKNRQLLPPLS--------------ELAKEESYFDIPNP----------------VLRRALSEFRKVVNALLEKYGG-------FHYFHIELTRDVCKAKSARMQLEKINKKNKSENDAAS---QLLEVLG----------------------------------------------LPNTYN-----NRLKCKLWKQQE---EYCLYSGEKITIDH-LKDQRALQIDHAFPLSR-SLDDSQSNKVLCLTSSNQEKSNK-------------TPYEWLG------SDEKKWDMYVGRVYSSNFSPS-------------------------------------KKRKLTQKNFKERNEEDFLARNLVD-----TGYIGRVTKEYIKHSLSFLPLPD-----------GKKEHIRIISGSMTSTMRSFWGVQ----------------------------------------------------------------------------EKNRDHHLHHAQDA-----IIIACIEPSMIQKYTTYLKDKETHRLKSHQKAQIL-------------REGDHK-------------LSLRWPMSNFKDKIQES------------------------------------------IQNIIPSHHVSHK------------------------------VTGELHQETVRTKEF-----------------------------------------------------------------------------------------------------------YYQAFGGEEG---------------------------VKKALKFGKIREINQGIVDN----------------GAMVRVDIFKSKDKGKFYAVPIYTYD------------------------FAIGKLPNKAIVQGKKNGIIKDWLEMDENYE----------------------FCFSLFKNDCIKIQTKEMQ--EAVLAIYKS-TN----------SAKATIELEHLSKYALKNEDEEKMFTDTDKEKNKTMT--------------------RESCGIQGLKVFQKVKLSVLGEVLEHKPRNRQNIALKTTPKHV------

>gi|221728729|gb|ACM31549.1|_CRISPR-associated_protein_Csn1_family_Acidovorax_ebreus_TPSY

M------------------------------------------------------------------AQHVFGLDIGIASVGWAILG---EQR----------IIDLGVRCFDKAE--------TAKEGDPLNLTRRQARLLRRRLYRRAWRLTQLSRLLKRKGLIA-----------------------------------------------------------DAKLFAKAP--SYGDSAWELRRQGLDRLLTPL------EWARVIYHQCKHRGFHWTSKAEEAKADSD-----------------------------------AEGGRVKQGLAHTKAL----------------------------------------------------------------------------------------------------------MQAKNYRSAAEMVLAEFPDAQ----------RNKRG-------------------------QYDKALSRVLLGEELALLFATQRRLGNPHAS-DFFEKLILGD--G---------------DRKSGLFWQQKPALSG---ADLLKMLGKCTFEKGEY--------------------------RAPKASFSVERHVWLTRLNNLRIVVDG------------------------------------------------RSRPLNEAERQ-------AALLLPYQTET--------SKYKTLKNAFIKAGLWGDGVRFGGLAYPSQA-----QIDAEKTKDPEDQFLVKLPAWHELRKAFKAAGHEALWQQ--------------------------------ISTP---ALDGDPTLLDQIATVLSVYKDGAEVVQQLRQLALP----EPAASIAVLEKISFDKFS--------------SLSLKALRRIVPLMQ----------------------SGLRYDEAVAQIPE-----------------------YGHHSQRIEPGAAKHLYLPPFYEAQRKYAGKGDHIGSMQFRDDADIPRNP----------------VVLRALNQARKVVNALIREYGS-------PIAVNIEMARDLSRPLDERNKVKRAQEEFRDRNDRAR---SEFER-------------------------------------------------DFGYK-PKAAAFEKWMLYREQL---GQCAYSQQPLDIQRVLDDHNYAQVDHALPYSR-SYDDSKNNKVLVLTHENQNKGNR-------------TAFEYLTSFPD-GEDGERWRTFVAWVQGNKAYRMA------------------------------------KRNRLLRKNYGVDESKGFIDRNLND-----TRYICKFFKNYVEEHLQLAARADG----------DTARRCVVVNGQLTAFLRARWGLT----------------------------------------------------------------------------KVRGDSDRHHALDA-----AVVAACTHGMVKALADYSRRKEISFLQEGFP-------------------DPETGEILNPAAFDRARQHFPEPWTHFAHELKARLFTD--------------DLAALREDMQRLG--SYTTEDLGRLRTLFVSRAPQRR------------------------------SGGAVHKETIYAQPESLKQQGGVIEKILLTSLKLQDFDKLLNPES----------------------------------------------------------NDHFVEPHRNERLYAAIRQRLEQFGGRADKAFGPDNLFHKPDKNNQPTGPVVRSIKLVRGKQTGIPIRG--GLAKN----------------DSMLRVDIFT--KAGKFHLVPVYVHH------------------------RVTG-LPNRAIVAFKD---EDEWTLIDESFA----------------------FLFSVYPNDYVKVTLKKEQ------------------------------QSGYYSGADRSTGAMNLWAHDRAASVGKDGL--------------------IRGIGVKTALSVEKFNVDVLGR-IYLAPPETRSGLA-------------

>gi|297182908|gb|ADI19058.1|_uncharacterized_protein_conserved_in_bacteria_uncultured_delta_proteobacterium_HF0070_07E19

M--------------------------------------------------SSKAIDSLEQLDLFKPQEYTLGLDLGIKSIGWAILS-------------GERIANAGVYLFETAEELN----STGNKLISKAAERGRKRRIRRMLDRKARRGRHIRYLLEREGLPTDELE-------------------------------------------------------------EVVVHQSNRTLWDVRAEAVERKLTKQ------ELAAVLFHLVRHRGYFPNTKKLPPDDESDSAD--------------------------------EEQGKINRATSRLREE----------------------------------------------------------------------------------------------------------LKASDCKTIGQFLAQNRDRQRN------------------------------------REGDYSNLMARKLVFEEALQILAFQRKQGHELSKDFEKTYLD--------------------------------VLMGQRSGRSPK--LGNCSLIPSEL--------------------------RAPSSAPSTEWFKFLQNLGNLQISNAYR-----------------------------------------------EEWSIDAPRRA--------QIIDACSQR-------STSSYWQIRRDFQIPDEYRFNLVNYERRDP-----------DVDLQEYLQQQERKTLANFRNWKQLEKIIGTG----------------------------------------------HPIQTLDEAARLITLIKDDEKLSDQLADLLP-----EASDKAITQLCELDFTTAA-------------KISLEAMYRILPHMN----------------------QGMGFFDACQQES-----------------------------------LPEIGVPPAGD-----------------RVPPFDEMYNP----------------VVNRVLSQSRKLINAVIDEYGM-------PAKIRVELARDLGKGRELRERIKLDQLDKSKQNDQRAEDFR-----------------------------------------------------AEFQQAPRGDQSLRYRLWKEQN---CTCPYSGRMIPVNSVLSED--TQIDHILPISQ-SFDNSLSNKVLCFTEENAQKSNR-------------TPFEYLD--------AADFQRLEAISGNWPEAKR----------------------------------------NKLLHKSFGKVAEEWKSRALND-----TRYLTSALADHLRHHLPDS-------------------KIQTVNGRITGYLRKQWGLE-----------------------------------------------------------------------------KDRDKHTHHAVDA-----IVVACTTPAIVQQVTLYHQDIR------------------------------RYKKLGEKRP--------TPWPETFRQDVLDV------------------------------------------EEEIFITRQPKKVS------------------------------GGIQTKDTLRKHRSKPDRQRVALTKVKLADLERLVEKDAS-----------------------------------------------------------------------NRNLYEHLKQCLEESGDQPTKAFKAPFYMPSGPEAKQRPILSKVTLLREKPEPPKQLTELSGGRRYDS---------------MAQGRLDIYRYKPGGKRKDEYRVVLQR-----------------------MIDLMRGEENVHVFQKGVPYDQGPEIEQNYT----------------------FLFSLYFDDLVEFQRSADS----------------------------EVIRGYYRTFNIANGQLKISTYLEGRQD-------------------------FDFFGANRLAHFAKVQVNLLGKVIK------------------------

>gi|154154505|gb|ABS61722.1|_CRISPR-associated_protein_Csn1_family_Parvibaculum_lavamentivorans_DS-1

M-------------------------------------------------------------------ERIFGFDIGTTSIGFSVIDYSSTQSAG-------NIQRLGVRIFPEAR---------DPDGTPLNQQRRQKRMMRRQLRRRRIRRKALNETLHEAGFLPAYGS-------------------------------------------------------------ADWPVVMADEPYELRRRGLEEGLSAY------EFGRAIYHLAQHRHFKGRELEESDTPDP-------------------------------------DVDDEKEAANERAATLK--------------------------------------------------------------------------------------------------------ALKNEQTTLGAWLARRPPSDR----------------------------------------KRGIHAHRNVVAEEFERLWEVQSKFHPALKSEEMRARIS-------------------------------DTIFAQRPVFWRKNTLGECRFMPGEP--------------------------LCPKGSWLSQQRRMLEKLNNLAIAGGN-------------------------------------------------ARPLDAEERD--------AILSKLQQQ-------ASMSWPGVRSALKALYKQRGEPGAEKSLKFNLELG-------GESKLLGNALEAKLADMFGPDWPAHPRKQEIRHAV--------------------------------HERL---WAADYGETPDKKRVIILSEKDRKAHREAAANSFVADFGITGEQAAQLQALKLPTGWE--------------PYSIPALNLFLAELE----------------------KGERFGALVNGPD---------------------------------WEGWRRTNFPHRNQPTGEILDKLPSPASKEERERISQLRNP----------------TVVRTQNELRKVVNNLIGLYGK-------PDRIRIEVGRDVGKSKREREEIQSGIRRNEKQRKKAT---EDLIKNG--------------------------------------------------IANPSRDDVEKWILWKEGQ---ERCPYTGDQIGFNALFREG-RYEVEHIWPRSR-SFDNSPRNKTLCRKDVNIEKGNR-------------MPFEAFGHDED------RWSAIQIRLQGMVSAKGG----------------------------------TGMSPGKVKRFLAKTMPEDFAARQLND-----TRYAAKQILAQLKRLWPDMGPEAP-------------VKVEAVTGQVTAQLRKLWTLNNILAD---------------------------------------------------------------------DGEKTRADHRHHAIDA-----LTVACTHPGMTNKLSRYWQLRD----------------------------DPRAEKPALTPP-----------WDTIRADAEKA------------------------------------------VSEIVVSHRVRKK------------------------------VSGPLHKETTYGDTGTDIKTKSGTYRQFVTRKKIESLSKG------------------------------------------------------------------ELDEIRDPRIKEIVAAHVAGRGGDPKKAFPP-------YPCVSPGGPEIRKVRLTSKQQLNLMAQTG------------------------------NGYADLGSNHHIAIYRLPD---------------------GKADFEIVSLFDASRRLAQRNPIVQRTRADGAS----------------------FVMSLAAGEAIMIPEGSKK-------------------------GIWIVQGVWASGQVVLERDTDADHSTTTRPMP----------------------------NPILKDDAKKVSIDPIGRVRPSND---------------------

>gi|121051596|emb|CAM07896.1|_hypothetical_protein_NMA0631_Neisseria_meningitidis_Z2491

M-----------------------------------------------------------AAFKPNPINYILGLDIGIASVGWAMVE---IDEDEN----PICLIDLGVRVFERAE--------VPKTGDSLAMARRLARSVRRLTRRRAHRLLRARRLLKREGVLQ-----------------------------------------------------------AADFDENGLIKSLPNTPWQLRAAALDRKLTPL------EWSAVLLHLIKHRGYLSQRKNEGETAD-------------------------------------KELGALLKGVADNAHA----------------------------------------------------------------------------------------------------------LQTGDFRTPAELALNKFEKESG-------HIRNQRG-------------------------DYSHTFSRKDLQAELILLFEKQKEFGNPHVS-GGLKEGIET------------------------LLMTQRPALSG---DAVQKMLGHCTFEPAEP--------------------------KAAKNTYTAERFIWLTKLNNLRILEQG------------------------------------------------SERPLTDTERA-------TLMDEPYRKSK--------LTYAQAR----KLLGLEDTAFFKGLRYG--------------KDNAEASTLMEMKAYHAISRALEKEGLKDKKSP--------------------------------LNL--------SPELQDEIGTAFSLFKTDEDITGRLKDRIQP----EILE--ALLKHISFDKFV--------------QISLKALRRIVPLME----------------------QGKRYDEACAEI-------------------------YGDHYG--KKNTEEKIYLPPIP---------------------ADEIRNP----------------VVLRALSQARKVINGVVRRYGS-------PARIHIETAREVGKSFKDRKEIEKRQEENRKDREKAA---AKFREYF----------------------------------------------PNFVGE-PKSKDILKLRLYEQQH---GKCLYSGKEINLGR-LNEKGYVEIDHALPFSR-TWDDSFNNKVLVLGSENQNKGNQ-------------TPYEYFN---G-KDNSREWQEFKARVETS-RFPRS------------------------------------KKQRILLQKFDED---GFKERNLND-----TRYVNRFLCQFVADRMRLTGKGK--------------KRVFASNGQITNLLRGFWGLR----------------------------------------------------------------------------KVRAENDRHHALDA-----VVVACSTVAMQQKITRFVRYKEMNAFDGKT-------------------IDKETGEVLHQK------THFPQPWEFFAQEVMIRVFGKPDGKPEFEEADTPEKLRTLLAEKLSSR--PEAVHEY--VTPLFVSRAPNRK------------------------------MSGQGHMETVKSAKR--LDEGVSVLRVPLTQLKLKDLEKMVNRE-------------------------------------------------------------------REPKLYEALKARLEAHKDDPAKAFAEP--FYKYDKAGNRT-QQVKAVRVEQVQKTGVWVRNHNGIADN----------------ATMVRVDVFE--KGDKYYLVPIYSWQ------------------------VAKGILPDRAVVQGKD---EEDWQLIDDSFN----------------------FKFSLHPNDLVEVITKKAR------------------------------MFGYFASCHRGTGNINIRIHDLDHKIGKNGI--------------------LEGIGVKTALSFQKYQIDELGKEIRPCRLKKRPPVR-------------

>gi|12721472|gb|AAK03211.1|_unknown_Pasteurella_multocida_subsp._multocida_str._Pm70

M--------------------------------------------------------------QTTNLSYILGLDLGIASVGWAVVE---INENED----PIGLIDVGVRIFERAE--------VPKTGESLALSRRLARSTRRLIRRRAHRLLLAKRFLKREGILS-----------------------------------------------------------TIDLEKG-----LPNQAWELRVAGLERRLSAI------EWGAVLLHLIKHRGYLSKRKNESQTNN-------------------------------------KELGALLSGVAQNHQL----------------------------------------------------------------------------------------------------------LQSDDYRTPAELALKKFAKEEG-------HIRNQRG-------------------------AYTHTFNRLDLLAELNLLFAQQHQFGNPHCK-EHIQQYMTE------------------------LLMWQKPALSG---EAILKMLGKCTHEKNEF--------------------------KAAKHTYSAERFVWLTKLNNLRILEDG------------------------------------------------AERALNEEERQ-------LLINHPYEKSK--------LTYAQVR----KLLGLSEQAIFKHLRYS--------------KENAESATFMELKAWHAIRKALENQGLKDTWQD--------------------------------LAK--------KPDLLDEIGTAFSLYKTDEDIQQYLTNKVPN----SVIN--ALLVSLNFDKFI--------------ELSLKSLRKILPLME----------------------QGKRYDQACREI-------------------------YGHHYG--EANQKTSQLLPAIP---------------------AQEIRNP----------------VVLRTLSQARKVINAIIRQYGS-------PARVHIETGRELGKSFKERREIQKQQEDNRTKRESAV---QKFKELF----------------------------------------------SDFSSE-PKSKDILKFRLYEQQH---GKCLYSGKEINIHR-LNEKGYVEIDHALPFSR-TWDDSFNNKVLVLASENQNKGNQ-------------TPYEWLQ---G-KINSERWKNFVALVLGS-QCSAA------------------------------------KKQRLLTQVIDDN---KFIDRNLND-----TRYIARFLSNYIQENLLLVGKNK--------------KNVFTPNGQITALLRSRWGLI----------------------------------------------------------------------------KARENNNRHHALDA-----IVVACATPSMQQKITRFIRFKEVHPYKIENRYEM---------------VDQESGEIISP--------HFPEPWAYFRQEVNIRVFDN--------------HPDTVLKEMLPDR--PQANHQF--VQPLFVSRAPTRK------------------------------MSGQGHMETIKSAKR--LAEGISVLRIPLTQLKPNLLENMVNKE-------------------------------------------------------------------REPALYAGLKARLAEFNQDPAKAFATP--FYK--QGG----QQVKAIRVEQVQKSGVLVRENNGVADN----------------ASIVRTDVFI--KNNKFFLVPIYTWQ------------------------VAKGILPNKAIVAHKN---EDEWEEMDEGAK----------------------FKFSLFPNDLVELKTKKEY------------------------------FFGYYIGLDRATGNISLKEHDGEISKGKDGV--------------------YR-VGVKLALSFEKYQVDELGKNRQICRPQQRQPVR-------------

>gi|405581662|gb|EKB55676.1|_CRISPR-associated_protein_cas9/csn1_subtype_II/nmemi_Bergeyella_zoohelcum_ATCC_43767

M-------------------------------------------------------------------KHILGLDLGTNSIGWALIERNIEEKYG-------KIIGMGSRIVPMGAELS---KFEQGQAQTKNADRRTNRGARRLNKRYKQRRNKLIYILQKLDMLPSQIKLKEDFSD-----------------------------------------PNKIDKITILPISKKQEQLTAFDLVSLRVKALTEKVGLE------DLGKIIYKYNQLRGYAGGSLEPEKEDIFDEEQSKDKKNKSFIAFSKIVFLGEPQEEIFKNKKLNRRAIIVETEEGNFEGSTFLENIKVG-------------------------------------------------------------------------------------------DSLELLINISASKSGDTITIKLPNKTNWRKKMENIENQLKEKSKEMGREFYISEFLLELLKENRWAKIRNNTILRARYESEFEAIWNEQVKHYPFLENLDKKTLIEIVSFIFPGEKESQKKYRELGLEKGLKYIIKNQVVFYQRELKDQSHLISDCRYEPNEK--------------------------AIAKSHPVFQEYKVWEQINKLIVNTKIEAG----------------------------------------------TNRKGEKKYKYIDRPIPTALKEWIFEELQN---KKEITFSAIFKKLKAEFDLREGIDFLNG-------------MSPKDKLKGNETKLQLQKSLGELWDVLGLDSIN---------------------------------------R---QIELWNILYNEKGNEYDLTSDRTSKVLEFINKYGNNIVDDNAEETAIRISKIKFARAY-----------S-SLSLKAVERILPLVRAGKYFNN------------DFSQQLQSKILKLLNENVEDPFAKAAQT-------YLDNNQSVLSEGGVGNSIATILVYDKHTAKEYSHDELYKSYKEINLLKQGDLRNP----------------LVEQIINEALVLIRDIWKNYGIK------PNEIRVELARDLKNSAKERATIHKRNKDNQTINNKIK---ETLVKNK---------------------------------------------------KELSLANIEKVKLWEAQR---HLSPYTGQPIPLSDLFDKE-KYDVDHIIPISR-YFDDSFTNKVISEKSVNQEKANR-------------TAMEYFEVG--------SLKYSIFTKEQFIAHVN-----------------------------------EYFSGVKRKNLLATSIPEDPVQRQIKD-----TQYIAIRVKEELNKIVGNE-------------------NVKTTTGSITDYLRNHWGLTDKFKLLLKERYEALLESEKFLEAEYDNYKKDFDSRKKEYEEKEVLFEEQELTREEFIKEYKENYIRYKKNKLIIKGWSKRIDHRHHAIDA-----LIVACTEPAHIKRLNDLNKVLQDWLVEHKSEFMPNFEGSN--SELLEEILSLPENERTEIFTQIEKFRAIEMPWKGFPEQVEQK------------------------------------------LKEIIISHKPKDKLLLQYNKAGDR----------------QIKLRGQLHEGTLYGISQGKEAYRIPLTKFGGSKFATEKNIQKIVSP--------------------------------------------------------------FLSGFIANHLKEYNNKKEEAFSAEGIMDLNNKLAQYRNEKGELKPHTPISTVKIYYKDPSKNKKKKDEEDLSLQ-------------KLDREKAFNEKLYVKTGDNYLFAVLEGEIKTKKTS--------------QIKRLYDIISFFDATNFLKEEFRNAPDKKTFDKDLLFRQYFEERN--------KAKLLFTLKQGDFVYLPNENEEVILDKESPLYNQYW----------GDLKERGKNIYVVQKFSKKQIYFIKHTIADIIKKDVEFGS-------------QNCYETVEGRSIKENCFKLEIDRLGNIVKVIKR--------------------

>gi|402266628|gb|EJU16049.1|_CRISPR-associated_protein_Cas9/Csn1_subtype_II/NMEMI_Porphyromonas_sp._oral_taxon_279_str._F0450

M----------------------------------------------------------------LMSKHVLGLDLGVGSIGWCLIA---LDAQ----GDPAEILGMGSRVVPLNNATKAIEAFNAGAAFTASQERTARRTMRRGFARYQLRRYRLRRELEKVGMLPDAA------------------------------------------------------------LIQLP--LLELWELRERAATAGRRLTLP------ELGRVLCHINQKRGYRH-----------------------------------------------VKSDAAAIVGDEGEKKK----------------------------------------------------------------------------------------------------DSNSAYLAGIRANDEKLQAEHKTVGQYFAEQLRQNQSESPT-------------------GGISYRIKDQIFSRQCYIDEYDQIMAVQRVHYPDILTDEFIRMLRD------------------------------EVIFMQRPLKSCKHLVSLCEFEK-QERVMRVQQDDGKGGWQLVERRVKFGPKVAPKSSPLFQLCCIYEAVNNIRLTRPN-----------------------------------------------GSPCDITPEERA--------KIVAHLQSS-------ASLSFAALKKLLKEKALIADQLTS-------------------KSGLKGNSTRVALASALQPYPQY------------------------------------------------------HHLLDMELETRMMTVQLTDEETGEVTEREV-----AVVTDSYVRKPLYRLWHI--------------LYSIEEREAMRRALITQL------------GMKEEDLDGGLLDQLYRLDFVKPGYGNKSAKF-------ICKLLPQLQQGLGYSEACAAVGYRHSN-SPTSEEITERTLLEKIPLLQRNELRQP----------------LVEKILNQMINLVNALKAEYG--------IDEVRVELARELKMSREERERMARNNKDREERNKGVA---AKIR-------------------------------------------------ECGLYPTKPR--IQKYMLWKEAG---RQCLYCGRSIEEEQCLREG-GMEVEHIIPKSV-LYDDSYGNKTCACRRCNKEKGNR-------------TALEYIRAKGR-------EAEYMKRINDLLKEKK-----------------------------------ISYSKHQRLRWLKEDIPSDFLERQLRL-----TQYISRQAMAILQQGIRR---------------------VSASEGGVTARLRSLWGYGKILHTLNLDRYDSMGETERVSR----EGEAT-------------------------------------EELHITNWSK-RMDHRHHAIDA-----LVVACTRQSYIQRLNRLSSEFGREDKKKEDQEAQEQQ-------ATETGRLSNLERWLTQRP---------HFS---VRTVSDK------------------------------------------VAEILISYRPGQRVVTRGRNIYRKKMADGREVSCVQRG--VLVPRGELMEASFYG----KILSQ---------GRVRIVKRYPLHDL---------------------------------------------------------------KGEVVDPHLRELITTYNQELKSREKGAP----IPPLCLDKDKKQEVRSVRCYAKTLSLDKAIPMCF----------------------DEKGE--PTAFVKSASNHHLALYR------------TPKG---------KLVESIVTFWDAVDRARYGIPLVITHPREVMEQVLQRGDIPEQVLSLLPPSDWVFVDSLQQDEMVVIGLS-------DEELQRALE-----------AQNYRKISEHLYRVQKMSSSYYVFRYHLETSVADDKN----------TSGRIPKFHRVQSLKAYEERNIRKVRVDLLGRISLL-----------------------

>gi|404335273|gb|EJZ61745.1|_CRISPR-associated_protein_cas9/csn1_subtype_II/nmemi_Barnesiella_intestinihominis_YIT_11860

M-------------------------------------------------------------------KNILGLDLGLSSIGWSVIR---ENS------EEQELVAMGSRVVSLT--AAELSSFTQGNGVSINSQRTQKRTQRKGYDRYQLRRTLLRNKLDTLGMLPDDS------------------------------------------------------------LSYLP--KLQLWGLRAKAVTQ--RIELN------ELGRVLLHLNQKRGYKS-----------------------------------------------IKSDFS------GDKK-------------------------------------------------------------------------------------------------------ITDYVKTVKTRYDELKEMRLTIGELFFRRLTEN---------------------------AFFRCKEQVYPRQAYVEEFDCIMNCQRKFYPDILTDETIRCIRD------------------------------EIIYYQRPLKSCKYLVSRCEFEK-RFYLNAAG------------KKTEAGPKVSPRTSPLFQVCRLWESINNIVVKDRR-----------------------------------------------NEIVFISAEQRA--------ALFDFLNTH-------EKLKGSDLLKLLGLSKTYGYRLGEQF-----------------KTGIQGNKTRVEIERALGNYPDK------------------------------------------------------KRLLQFNLQE--ESSSMVNTETGEIIP---------MISLSFEQEPLYRLWHV--------------LYSIDDREQLQSVLRQKF------------GID----DDEVLERLSAIDLVKAGFGNKSSKA-------IRRILPFLQLGMNYAEACEAAGYNHSN-NYTKAENEARALLDRLPAIKKNELRQP----------------VVEKILNQMVNVVNALMEKYGR-------FDEIRVELARELKQSKEERSNTYKSINKNQRENEQIA---KRIV-------------------------------------------------EYG-VPTRSR--IQKYKMWEESK---HCCIYCGQPVDVGDFLRGF-DVEVEHIIPKSL-YFDDSFANKVCSCRSCNKEKNNR-------------TAYDYMKSKGEK-----ALSDYVERVNTMYTNNQ-----------------------------------ISKTKWQNLLTPVDKISIDFIDRQLRE-----SQYIARKAKEILTSICYN---------------------VTATSGSVTSFLRHVWGWDTVLHDLNFDRYKKVGLTEVIEVNH--RGSVI-------------------------------------RREQIKDWSK-RFDHRHHAIDA-----LTIACTKQAYIQRLNNLRAEEGPDFNKMS------------------------LERYIQSQP---------HFS---VAQVREA------------------------------------------VDRILVSFRAGKRAVTPGKRYIRK----NRKRISVQS---VLIPRGALSEESVYG----VIHVWEKDEQGHVIQKQRAVMKYPITSINREMLD---------------------------------------------------------KEKVVDKRIHRILSGRLAQYNDNPKEAF----AKPVYIDKECRIPIRTVRCFAKP-AINTLVPLKK----------------------DDKGN--PVAWVNPGNNHHVAIYR------------DEDG---------KYKERTVTFWEAVDRCRVGIPAIVTQPDTIWDNILQRNDISENVLESLPDVKWQFVLSLQQNEMFILGMN-------EEDYRYAMD-----------QQDYALLNKYLYRVQKLSKSDYSFRYHTETSVEDKYDGKPNLK----LSMQMGKLKRVS-IKSLLGLNPHKVHISVLGEIKEIS----------------------

>gi|373227567|gb|EHP49880.1|_CRISPR-associated_protein_cas9/csn1_subtype_II/nmemi_Odoribacter_laneus_YIT_12061

M-------------------------------------------------------------------ETTLGIDLGTNSIGLALVD-----------QEEHQILYSGVRIFPEGINKDTIG--LGEKEESRNATRRAKRQMRRQYFRKKLRKAKLLELLIAYDMCPLKPEDVRRWKN---------------------------------------WDKQQKSTVRQFPDTPAFREWLKQNPYELRKQAVTEDVTRP------ELGRILYQMIQRRGFLSSRKGKEEGKIFTGKD---------------------------------RMVGIDETRKNLQKQTLG------------------------------------------------------------------------------------------------------AYLYDIAPKNGEKYRFRTERVRARYTLRDMYIREFEIIWQRQAGHLGLAHEQATRKKNIFLEGSATNVRNSKLITHLQAKYGRGHVLIEDTRITVTFQLPLKEVLGG----------KIEIEEEQLKFKSNESVLFWQRPLRSQKSLLSKCVFEGRNFYDPVHQK------------WIIAGPTPAPLSHPEFEEFRAYQFINNIIYGKNE---------------------------------------------------HLTAIQRE--------AVFELMCTES------KDFNFEKIPKHLKLFEKFNFDDTTKVP------------ACTTISQLRKLFPHPVWEEKREEIWHCFYFYDDN------------------------------------------------TLLFEKLQKDYALQTNDLEKIKKIRLSESYG--NVSLKAIRRINPYLKKGYAY-----------STAVLLGGIRNSFGKRFEYFK-----------------EYEPEIEKAVCRILKEKNAEG---------------EVIRKIKDYLVHNRFGFAKNDRAFQKLYHHSQAITTQAQKERLPETGNLRNP----------------IVQQGLNELRRTVNKLLATCREKYGPSFKFDHIHVEMGRELRSSKTEREKQSRQIRENEKKNEAAK---VKLAEYG------------------------------------------------LKAYRDNIQKYLLYKEIEEKGG-TVCCPYTGKTLNISHTLGSDNSVQIEHIIPYSI-SLDDSLANKTLCDATFNREKGEL-------------TPYDFYQKDPS----PEKWGASSWEEIEDRAFRLLP-----------------------------------YAKAQRFIRRKPQESNEFISRQLND-----TRYISKKAVEYLSAICSD---------------------VKAFPGQLTAELRHLWGLNNILQSAPDITFPLPVSATENHREYYVITNEQNEVIRLFPKQGETPRTE--------------------KGELLLTGEVERKVFRCKGMQE-----FQTDVSDGKYWRRIKLSSSVTWSPLFAPKPISADGQIVLKGRIEKGVFVCNQLKQKLKTGLPDGSYWISLPVISQTFKEGESVNNSKLTSQQVQLFGRVREGIFRCHNYQCPASGADGNFWCTLDTDTAQPAFTPIKNAPPGVGGGQIILTGDVDDKGIFHADDDLHYELPASLPKGKYYGIFTVESCDPTLIPIELSAPKTSKGENLIEGNIWVDEHTGEVRFDPKKNREDQRHHAIDAIVIALSSQSLFQRLSTYNARRENKKRGLDSTEHFPSPWPGFAQDVRQSVVPLLVSYKQNPKTLCKISKTLYKDGKKIHSCGNAVRGQLHKETVYGQRTAPGATEKSYHIRKDIRELKTSKHIGKVVDITIRQMLLKHLQENYHIDITQEFNIPSNAFFKEGVYRIFLPNKHGEPVPIKKIRMKEELGNAERLKDNINQYVNPRNNHHVMIYQDADGNLKEEIVSFWSVIERQNQGQPIYQLPREGRNIVSILQINDTFLIGLKEEEPEVYRNDLSTLSKHLYRVQKLSGMYYTFRHHLASTLNNEREEFR-----------------IQSLEAWKRANPVKVQIDEIGRITFLNGPLC------------------

>gi|496396787|ref|WP_009105777.1|_type_II_CRISPR_RNA-guided_endonuclease_Cas9_Treponema_sp._JC4

M--------------------------------------------------------------IMKLEKWRLGLDLGTNSIGWSVFS---LDKDNS----VQDLIDMGVRIFSDGR--------DPKTKEPLAVARRTARSQRKLIYRRKLRRKQVFKFLQEQGLFPKTK--------------------------------------------------------------EECMTLKSLNPYELRIKALDEKLEPY------ELGRALFNLAVRRGFKSNRKDGSREEVSEK----------------------------------KSPDEIKTQADMQTHLEK-------------------------------------------------------------------------------------------------------AIKENGCRTITEFLYKNQGENG--------GIRFAPG-------------------------RMTYYPTRKMYEEEFNLIRSKQEKYYPQVDWDDIYK-----------------------------------AIFYQRPLKPQQ--RGYCIYENDKE--------------------------RTFKAMPCSQKLRILQDIGNLAYYEGGS----------------------------------------------KKRVELNDNQDK--------VLYELLNSK-------DKVTFDQMRKALCLADSNSFNLEENRD-------------------FLIGNPTAVKMRSKNRFGKLWDEIPLE--------------------------------------------------EQDLIIETIITADEDDAVYEVIKKYDLT----QEQRDFIVKNTILQSGTS--------------MLCKEVSEKLVKRLEE--------------------IADLKYHEAVESLG---------------------------YKFADQTVEKYDLLPYYGKVLPGSTMEIDLSAPETNPEKHYGKISNP----------------TVHVALNQTRVVVNALIKEYGK-------PSQIAIELSRDLKNNVEKKAEIARKQNQRAKENIAINDTISALYHTAFP----------------------------------------------GKSFYPNRNDRMKYRLWSELG-LGNKCIYCGKGISGAELFTK--EIEIEHILPFSR-TLLDAESNLTVAHSSCNAFKAER-------------SPFEAFGTNPS----GYSWQEIIQRANQLKNTSKK----------------------------------NKFSPNAMDSFEKDS---SFIARQLSD-----NQYIAKAALRYLKCLVENPS------------------DVWTTNGSMTKLLRDKWEMDSILCRKFTEKEVALLGLKPEQIGN---------------------------------------------------YKKNRFDHRHHAIDA-----VVIGLTDRSMVQKLATKNSHKGN----------------------------------RIEIP----------EFPILRSDLIEK------------------------------------------VKNIVVSFKPDHG------------------------------AEGKLSKETLLGKIK--------LHGKETFVCRENIVSLS----------------------------------------------------------------EKNLDDIVDEKIKSKVKDYVAKHKGQKIEAVLSDFSKENGIKKVRCVNRVQTPIEITSGKISRYLSPED------------------------YFAAVIWEIPGEKKTFKAQYIRR---------------------------NEVEKNSKGLNVVKPAVLENGKPHPAAKQ-----------------------VCLLHKDDYLEFSDKGKMYFCRIAGYAATNNK----------LDIRPVYAVSYCADWINSTNETMLTGYWKPTPTQN---------------------WVSVNVLFDKQKARLVTVSPIGRVFRK-----------------------

>gi|495662404|ref|WP_008386983.1|_type_II_CRISPR_RNA-guided_endonuclease_Cas9_Rhodovulum_sp._PH10

M-------------------------------------------------------------------GIRFAFDLGTNSIGWAVWR---TGPGVFGEDTAASLDGSGVLIFKDG--------RNPKDGQSLATMRRVPRQSRKRRDRFVLRRRDLLAALRKAGLFPVDVE-------------------------------------------------------------EGRRLA-ATDPYHLRAKALDESLTPH------EMGRVIFHLNQRRGFRSNRKADRQDR---------------------------------------EKGKIAEGSKRLAET----------------------------------------------------------------------------------------------------------LAATNCRTLG-EFLWSRHRGTPR---TRSPTRIR------MEGEG-------------AKALYAFYPTREMVRAEFERLWTAQSRFAPDLLTPERHEEIA-------------------------------GILFRQRDLAPPK--IGCCTFE--------------------------PSERRLPRALPSVEARGIYERLAHLRITTGP-----------------------------------------------VSDRGLTRPERD--------VLASALLAG-------KSLTFKAVRKTLK----ILPHALVNFE-------------EAGEKGLDG-ALTAKLLSKPDHYGAAWHGLSFA-----------------------------------------------EKDTFVG---KLLDEADEERLIRRLVTENR----LSEDAARRCASIPLADGYG--------------RLGRTANTEILAALVEETDET---------------GTVVTYAEAVRRAGERTGR-------------------NWHHSDE-RDGVILDRLPYYGEILQRHVVPGSGEPEEKNEAARWGRLANP----------------TVHIGLNQLRKVVNRLIAAHGR-------PDQIVVELARELKLNREQKERLDRENRKNREENERRT---AILAEHG------------------------------------------------QRDTAENKIRLRLFEEQARANAGIALCPYTGRAIGIAELFTS--EVEIDHILPVSL-TLDDSLANRVLCRREANREKRRQ-------------TPFQAFG-ATP------AWNDIVARAAKLPPNKR-----------------------------------WRFDPAALERFEREG---GFLGRQLNE-----TKYLSRLAKIYLGKICDPD-------------------RVYVTPGTLTGLLRARWGLNSILS------DSN---------------------------------------------------------------FKNRSDHRHHAVDA-----VVIGVLTRGMIQRIAHDAARAE----------------------------DQDLDRVFRDVP---------VPFEDFRDHVRER------------------------------------------VSTITVAVKPEHG------------------------------KGGALHEDTSYGLVPDTDPN----AALGNLVVRKPIRSLT---------------------------------------------------------------A-GEVDRVRDRALR-ARLGALAAPFRDESGRVRDAKGLAQALEAFG-AENGIRRVRILKPDASVVTIADR------------------------RTGVPYR-AVAPGENHHVDIVQMR----------DGS-----------WRGFAASVFEVNR-PGWR---P---EWEVKKLGG------------------KLVMRLHKGDMVELSDKDGQ---RRVKVVQQIEI----------SANRVRLSPHNDGGKLQDRHAD--ADDPFRWDL-------------------------ATIPLLKDRGCVAVRVDPIGVVT---LRRSNV----------------

>Riemerella_anatipestifer_RA-CH-2

M-------------------------------------------------------------------KTILGLDLGTNSIGWALVK---ETENS---NEKSEIIKLGVRVNPLT--VDEKTNFEAGRPLSTNADRTAKRSARRNLQRYKLRRKNLIDLLIKHRLIDKDT------------------------------------------------------------PLTEIGKNTTHQTLELRAKAARERIELE------DLARVFLAINKKRGYRSSRKVNNEEEGQVVDGMAVAKKLYDENLTPGQYAYELLSKGKKYVPDFYRSDLQAEFDSIWEYQKQFYADILDDELYNALKGQGQQNSR---KRFLAIKGVYTAENK--------------------GKRDEVKLQHYKWRSEAITQKLSIEEVAYVLVEINNDLNKSSGYLGAISDRSKELYFNKETVGENLWKQIQKN---------------------------PHTSLKNQVFYRQDYLDEFEQIWETQAQFHP-QLTLALKEQIRD------------------------------VVIFYQRKLKSQKGLLSFCQFESWEIERKDENGNVILNKTTQLPKRQTVGRRVAPKSSPLFQEFKIWQNINNLEIAKIS----------------------------------------DGNSKNKKETEALSDDERK--------LLFEELNLR-------GNLSQKEVLQILGLKD---KEYKTNF-----------------PEGLEGNRTNAALFNIYQQIAENEGYG-DW-----------------------------------------------TKKSAQEIKEELKAVFPQIGIQADILDFNA-----ELDGKEFENQASYQLWHL--------------LYSAEEDDKINEEDQ---IIYGNSAVSLKKKLCEKFGFTPEYAKWIANVSLQDDYGNLSTKA-------MRKIIPYLIDGNDYSEACALAGYNHSN-SLTKEENDNRERLNKLELLPKNSLRNP----------------VVEKILNQMVNVVNQVIETYGK-------PDEVRIELARELKKSAEERAEMTKGINEATRRNEDIK---KLIT-------------------------------------------------KNFGIPNPTKNDVVRYRLWEELAPLAYKDVFTGRQIKKEDLFSS--KIDIEHIIPKAL-LFDDSFSNKTLAFRETNLKKADR-------------TAYGFIESDYN-----ATLDDYIQRVETLYNNAKG---------------------------------TISKGKRNKLLMAQKNLPDGFIERDLRN-----SQYIAKKAKSMIEKVFND---------------------VNVTSGSITDRLREDWDLINVMKELNFPKYKALGLTEIEERYDIGQEKTK-------------------------------------KVEVITDWTK-RNDHRHHAMDA-----LTVAFTTKSHVQYLNNLNTIHSLKGDEVDLELSKLYG-------LKNTITEVVNKKRKFIPP---------MPN--FREEAKKH------------------------------------------IETILVSIKNKNKVYTKNINKTKK------ESGYHTKE--QLTPRGQLHKETVYGKSKRPMNKPTKINKNFSLEQAQLIINVQEKELVLRHLAQFDNNPAVAFDTK--TLKKMPLLKNGEPLKEV-------LCFEEIFTIRKDITPDLKIEKVVDEKIKAILEQRKKDFGGNAKEAFSDLDKNPIWLNKEKGICIKRVTITGVSNAEALHHQKDHLGREIL----------------DDNGNPIPADFVSTGNNHHVAIYE------------DEKG---------KLQEKVVSLYEAVARANQGLPVVDKGLNEDLG--------------------WKFLFTMKQNEMFVFPND--D---FDVNDIDLLD-----------ENNAKIISQHLFRVQKLATKNYMFRHHLETNVQEVKELN--------------DIAYKSIRSTEPLKNIKKVRINHIGKIVQVGEY*-------------------

**References**

1. Wang X, Ding C, Wang S, Han X, Yu S: **Whole-Genome Sequence Analysis and Genome-Wide Virulence Gene Identification of *Riemerella anatipestifer* Strain Yb2**. *Applied & Environmental Microbiology* 2015, **81**(15):5093-5102.

2. Yuan J, Liu W, Sun M, Song S, Cai J, Hu S: **Complete genome sequence of the pathogenic bacterium *Riemerella anatipestifer* strain RA-GD**. *Journal of bacteriology* 2011, **193**(11):2896-2897.

3 Wang X, Liu W, Zhu D, Yang L, Liu M, Yin S, Wang M, Jia R, Chen S, Sun K *et al*: **Comparative genomics of *Riemerella anatipestifer* reveals genetic diversity**. *BMC Genomics* 2014, **15**:479.

4. Mavromatis K, Lu M, Misra M, Lapidus A, Nolan M, Lucas S, Hammon N, Deshpande S, Cheng JF, Tapia R: **Complete genome sequence of *Riemerella anatipestifer* type strain (ATCC 11845^T^ )**. *Standards in Genomic Sciences* 2011, **4**(2):144-153.

5. Wang X, Zhu D, Wang M, Cheng A, Jia R, Zhou Y, Chen Z, Luo Q, Liu F, Wang Y: **Complete genome sequence of *Riemerella anatipestifer* reference strain**. *Journal of bacteriology* 2012, **194**(12):3270-3271.

6. Wang X, Ding C, Han X, Wang S, Yue J, Hou W, Cao S, Zou J, Yu S: **Complete Genome Sequence of *Riemerella anatipestifer* Serotype 1 Strain CH3**. *Genome Announcements* 2014, **3**(1):e01594-01514.
